# Supplementary material for: Digital delivery of behavioural activation therapy to overcome depression and facilitate social and economic transitions of adolescents in South Africa (the DoBAt study): protocol for a pilot randomised controlled trial
Source: BMJ Open. 2022 Dec 30;12(12):e065977. doi: 10.1136/bmjopen-2022-065977 (PMC9809228; doi:10.1136/bmjopen-2022-065977)
Supplement: Author's manuscript [file bmjopen-2022-065977.draft_revisions.pdf]

BMJ Open is committed to open peer review. As part of this commitment we make the peer review history of every article we publish publicly available.

When an article is published we post the peer reviewers' comments and the authors' responses online. We also post the versions of the paper that were used during peer review. These are the versions that the peer review comments apply to.

The versions of the paper that follow are the versions that were submitted during the peer review process. They are not the versions of record or the final published versions. They should not be cited or distributed as the published version of this manuscript.

BMJ Open is an open access journal and the full, final, typeset and author-corrected version of record of the manuscript is available on our site with no access controls, subscription charges or pay-per-view fees (http://bmjopen.bmj.com).

If you have any questions on BMJ Open's open peer review process please email info.bmjopen@bmj.com

BMJ Open

Digital delivery of Behavioural Activation therapy to overcome depression and facilitate social and economic transitions of adolescents in South Africa (the DoBAt study): protocol for a pilot randomised controlled trial

Journal:	*BMJ Open*
Manuscript ID	bmjopen-2022-065977
Article Type:	Protocol
Date Submitted by the Author:	22-Jun-2022
Complete List of Authors:	Moffett, Bianca; University of the Witwatersrand, MRC/Wits Rural Public Health and Health Transitions Research Unit (Agincourt), School of Public Health, Faculty of Health Sciences<br>Pozuelo, Julia; University of Oxford, Psychiatry; University of the Witwatersrand, MRC/Wits Rural Public Health and Health Transitions Research Unit (Agincourt), School of Public Health, Faculty of Health Sciences<br>van Heerden , Alastair ; Human Sciences Research Council, Center for Community Based Research<br>O'Mahen, Heather; University of Exeter, Mood Disorders Centre, Department of Psychology<br>Craske, Michelle; University of California Los Angeles, Psychology, Psychiatry and Biobehavioral Sciences<br>Sodi, Tholene; University of Limpopo, Psychology<br>Lund, Crick; University of Cape Town, Alan J Flisher Centre for Public Mental Health, Department of Psychiatry and Mental Health; King's College London, Centre for Global Mental Health, Institute of Psychiatry, Psychology and Neuroscience<br>Orkin, Kate; University of Oxford, Blavatnik School of Government<br>Kilford, Emma J; University College London, Department of Clinical, Educational and Health Psychology<br>Blakemore, Sarah-Jayne; University of Cambridge, Department of Psychology; University of the Witwatersrand, MRC/Wits Rural Public Health and Health Transitions Research Unit (Agincourt), School of Public Health, Faculty of Health Sciences<br>Mahmud, Mahreen; University of Exeter, Department of Economics<br>Musenge, Eustasius; University of the Witwatersrand, MRC/Wits Rural Public Health and Health Transitions Research Unit (Agincourt), School of Public Health, Faculty of Health Sciences; University of the Witwatersrand, Division of Epidemiology and Biostatistics, School of Public Health, Faculty of Health Sciences<br>Davis, Meghan; University of Oxford, Department of Psychiatry<br>Makhanya, Zamakhanya; University of the Witwatersrand, MRC/Wits Rural Public Health and Health Transitions Research Unit (Agincourt), School of Public Health, Faculty of Health Sciences<br>Baloyi, Tlangelani; University of Limpopo, Department of Psychology<br>Mahlangu, Daniel; University of the Witwatersrand, MRC/Wits Rural Public Health and Health Transitions Research Unit (Agincourt), School of

Public Health, Faculty of Health Sciences
Chierchia, Gabriele; University of Cambridge, Department of Psychology
Fielmann, Sophie L.; University of Cambridge, Department of Psychology
Gómez-Olivé, F. Xavier; University of the Witwatersrand, MRC/Wits Rural Public Health and Health Transitions Research Unit (Agincourt), School of Public Health, Faculty of Health Sciences
Valodia, Imraan; University of the Witwatersrand, Southern Centre for Inequality Studies
Tollman, Stephen; University of the Witwatersrand, MRC/Wits Rural Public Health and Health Transitions Research Unit (Agincourt), School of Public Health, Faculty of Health Sciences; Umeå Universitet, Umeå Centre for Global Health Research, Division of Epidemiology and Global Health, Department of Public Health and Clinical Medicine
Kahn, Kathleen; University of the Witwatersrand, MRC/Wits Rural Public Health and Health Transitions Research Unit (Agincourt), School of Public Health, Faculty of Health Sciences; Umeå Universitet, Umeå Centre for Global Health Research, Division of Epidemiology and Global Health, Department of Public Health and Clinical Medicine
Stein, Alan; University of Oxford, Department of Psychiatry; University of the Witwatersrand, MRC/Wits Rural Public Health and Health Transitions Research Unit (Agincourt), School of Public Health, Faculty of Health Sciences

Keywords: Depression & mood disorders < PSYCHIATRY, Child & adolescent psychiatry < PSYCHIATRY, PUBLIC HEALTH

SCHOLARONE™
Manuscripts

Digital delivery of Behavioural Activation therapy to overcome depression and facilitate social and economic transitions of adolescents in South Africa (the DoBAt study): protocol for a pilot randomised controlled trial

Authors
Bianca D. Moffett*[1], Julia R. Pozuelo*[1,2], Alastair van Heerden[3], Heather A. O'Mahen[4], Michelle Craske[5], Tholene Sodi[6], Crick Lund[7,8], Kate Orkin[9], Emma J Kilford[10], Sarah-Jayne Blakemore[1,11], Mahreen Mahmud[12], Eustasius Musenge[1,16], Meghan Davis[2], Zamakhanya Makhanya[1], Tlangelani Baloyi[6], Daniel Mahlangu[1] Gabriele Chierchia[11], Sophie L. Fielmann[11], Xavier Gomez-Olive[1], Imraan Valodia[13], Stephen Tollman[1,14], Kathleen Kahn**[1,14], Alan Stein**[1,2,15]

* denotes joint first author
** denotes joint senior author

Affiliations
[1] MRC/Wits Rural Public Health and Health Transitions Research Unit (Agincourt), School of Public Health, Faculty of Health Sciences, University of the Witwatersrand, Johannesburg, South Africa
[2] Department of Psychiatry, University of Oxford, Oxford, United Kingdom
[3] Center for Community Based Research, Human Sciences Research Council, Pietermaritzburg, South Africa
[4] Mood Disorders Centre, Department of Psychology, University of Exeter, Exeter, United Kingdom
[5] Department of Psychology, Psychiatry and Biobehavioral Sciences at the University of California, Los Angeles, United States of America
[6]Department of Psychology, University of Limpopo, Polokwane, South Africa
[7]Alan J Flisher Centre for Public Mental Health, Department of Psychiatry and Mental Health, University of Cape Town, Cape Town, South Africa
[8] Centre for Global Mental Health, Institute of Psychiatry, Psychology and Neuroscience, King's College London, London, United Kingdom
[9] Blavatnik School of Government, University of Oxford, Oxford, United Kingdom
[10] Department of Clinical, Educational and Health Psychology, University College London, London, United Kingdom
[11] Department of Psychology, University of Cambridge, Cambridge, United Kingdom
[12]Department of Economics, University of Exeter, Exeter, United Kingdom
[13]Southern Centre for Inequality Studies, University of the Witwatersrand, Johannesburg, South Africa
[14] Umeå Centre for Global Health Research, Division of Epidemiology and Global Health, Department of Public Health and Clinical Medicine, Umeå University, Umeå, Sweden
[15]Africa Health Research Institute, KwaZulu Natal, South Africa
[16]Division of Epidemiology and Biostatistics, School of Public Health, Faculty of Health Sciences, University of the Witwatersrand, Johannesburg, South Africa

Corresponding author: Bianca D Moffett, MRC/Wits Rural Public Health and Health Transitions Research Unit (Agincourt), School of Public Health, Faculty of Health Sciences, University of the Witwatersrand, Johannesburg, South Africa, bianca.moffett@wits.ac.za

Word Count
Abstract: 300
Text: 4355 (Excl. References, Tables and Figures)
Tables & Figures: 1 Table, 1 Figure
References: 63

Keywords: depression, adolescents, pilot trial, feasibility, digital, app, Behavioural Activation

For peer review only - http://bmjopen.bmj.com/site/about/guidelines.xhtml

ABSTRACT

Introduction: Scalable psychological treatments to address depression amongst adolescents are urgently needed. This is particularly relevant to low- and middle-income countries where 90% of the world's adolescents live, socioeconomic adversities affecting mental ill-health are prevalent, and mental health service resources remain very limited. Whilst digital delivery of Behavioural Activation presents a promising solution, its feasibility, acceptability, and effectiveness remain to be shown.

Methods and analysis: This study is a two-arm single-blind individual-level randomised controlled pilot trial to assess the feasibility, acceptability, and initial efficacy of digitally delivered Behavioural Activation (BA) therapy amongst adolescents with depression. The intervention has been co-produced with adolescents at the study site. The study is based in the rural north-east of South Africa in the Bushbuckridge sub-district of Mpumalanga Province. A total of 200 adolescents with symptoms of mild to moderately-severe depression on the Patient Health Questionnaire Adolescent Version (PHQ-A) will be recruited (1:1 allocation ratio). The treatment group will receive digitally delivered Behavioural Activation (the Kuamsha app programme) whilst the control group will receive an Enhanced Standard of Care. The feasibility and acceptability of the intervention will be evaluated using a mixed-methods design, and signals of initial efficacy of the intervention in reducing symptoms of depression will be determined on an intention-to-treat basis. Secondary objectives are to pilot a range of cognitive, mental health, risky behaviour, and socioeconomic measures; and to collect descriptive data on the feasibility of trial procedures to inform the development of a further larger trial.

Ethics and dissemination: This study has been approved by the University of the Witwatersrand Human Research Ethics Committee (MED20-05-011) and the Oxford Tropical Research Ethics Committee (OxTREC 34-20). Study findings will be published in scientific open access peer-reviewed journals, presented at scientific conferences, and communicated to participants, their caregivers, public sector officials, and other relevant stakeholders.

Trial registration: This trial was registered on 19 November 2020 with the South African National Clinical Trials Registry (DOH-27-112020-5741) and the Pan African Clinical Trials Registry (PACTR202206574814636).

STRENGTHS AND LIMITATIONS

- This study will address a critical evidence gap on managing adolescent depression in a low-resource African context by determining the feasibility, acceptability, and initial efficacy of digitally delivered Behavioural Activation therapy.
- The intervention has been iteratively co-produced with local adolescents, clinicians and service/policy leads to ensure acceptability, cultural relevance, useability, and face validity of the intervention with the targeted population.
- Novel culturally adapted measures of executive function and social cognition, as well as a range of relevant mental health, risky behaviours, and socioeconomic measures will be piloted.

For peer review only - http://bmjopen.bmj.com/site/about/guidelines.xhtml

- This study is situated in a rural, socioeconomically disadvantaged area of South Africa, a middle-income country. Important contextual differences should be considered when evaluating the implications for other low- and middle-income countries and African populations.

1. INTRODUCTION

Despite growing acknowledgement of the importance of adolescent mental health and the potential of investing in this formative period, it remains severely neglected[1]. This is particularly true in low and middle-income countries (LMICs), where 90% of the world's adolescents live, socioeconomic adversities affecting mental health are prevalent, and mental health resources remain poor[2]. Given that most mental disorders have their onset before 25 years of age, early identification and treatment of emergent mental disorders has the potential to reduce chronicity and sequelae for individuals, and be an efficient strategy for addressing population-level mental health[3]. Investments in mental health have the potential to support socioeconomic transitions and contribute to breaking the cycle of poverty and mental ill-health in LMICs[4].

Adolescence is a critical developmental period during which individuals develop their self-identity, acquire skills and preferences with which they navigate future challenges, and make decisions that can affect their long-term health, education, relationships, and employment prospects[5,6]. Higher-order cognitive functions such as executive function and social cognition, which regulate the ability to strategise, set and maintain goals, and build successful relationships with others, develop significantly during this period[7]. Thus, the physical, cognitive, social, and emotional capabilities acquired during adolescence lay a foundation for the wellbeing of individuals throughout their adult lives[8]. Depression interferes with the acquisition of these capacities, thus limiting young people's ability to fulfil their potential.

Globally, depression is one of the leading causes of disability amongst adolescents, and suicide is the third leading cause of death amongst the 15-19 year age group[2,9]. Left untreated, depression affects interpersonal relationships, interferes with schooling, and disrupts productivity[10]. It also has a marked negative effect on executive function and social cognition, with depressed individuals consistently underperforming on cognitive assessments compared to healthy controls[11,12]. Furthermore, depression in adolescence has been associated with a greater risk for substance use, poor sexual health, delinquency, and a significant reduction in future income[13,14]. Scalable psychological therapies to address depression amongst adolescents, particularly in low-resource contexts, are urgently needed.

Several barriers hinder progress in providing treatment to adolescents with depression in LMICs. Firstly, whilst various treatments for depression have been shown to be effective amongst adolescents in high-income contexts, few have been adapted for or rigorously evaluated in low-resource or culturally diverse settings[15,16]. Secondly, there is a major shortage of mental health professionals in most LMICs[17], and although task-sharing approaches present a promising strategy, they require substantial inputs in training and supervision of non-specialist healthcare workers[18]. Thirdly, there are significant challenges associated with relying on an

For peer review only - http://bmjopen.bmj.com/site/about/guidelines.xhtml

already overburdened healthcare sector, not least that depression is rarely detected in primary care in most LMICs[19]. Finally, even in contexts where adolescent mental health services are available, stigma, lack of mental health awareness, and issues with the acceptability of services prevent adolescents from seeking care[20].

Digitally delivered Behavioural Activation (BA) therapy offers the potential to overcome a number of these barriers. BA is a highly effective psychological therapy that is easier to deliver and less costly than Cognitive Behavioural Therapy (CBT)[21,22]. Importantly, there is also evidence that it can be effectively adapted for use: 1) with adolescents[23,24], 2) in low-resource contexts and diverse cultural settings[25–27] and 3) in a digital format[28]. Delivering BA in a digital format may be particularly helpful in contexts where there is a paucity of mental health specialists and health systems are already juggling multiple competing priorities. It also offers the potential to overcome internalised barriers to care, such as stigma and lack of mental health awareness, and this form of guided self-help may be more acceptable to adolescents[20].

Whilst digital interventions present a promising approach to addressing depression amongst adolescents, few have been specifically developed for use, or rigorously evaluated, in an LMIC context[29,30]. Furthermore, several questions regarding their feasibility and acceptability remain. Studies of online self-help interventions amongst adults in LMICs showed high attrition rates[29], and adherence to mental health apps in all contexts is often low[31]. This study will address an important gap in the literature by providing evidence on the feasibility, acceptability, and initial efficacy of using digitally delivered Behavioural Activation to address depression amongst adolescents in an LMIC context. Furthermore, we will pilot a range of relevant mental health, risky behaviour and socioeconomic measures, as well as novel culturally adapted measures of executive function and social cognition.

Study objectives

The co-primary objectives of this pilot study are: 1) to determine the feasibility and acceptability of a digitally delivered Behavioural Activation therapy intervention for adolescents living with depression in the Bushbuckridge sub-district of Mpumalanga province, South Africa; and 2) to provide preliminary evidence on any signals of initial efficacy (direction and magnitude) of any effects of the intervention on depressive symptoms amongst adolescents in the intervention arm compared to control.

The secondary objectives are: 1) to pilot a range of mental health, social-affective cognition, risky behaviours, and socioeconomic measures; and 2) to collect descriptive data on trial procedures such as recruitment, retention, data collection, randomisation and blinding to inform key parameters in the development of a further large-scale trial.

2. METHODS AND ANALYSIS

This protocol is reported in accordance with the Standard Protocol Items: Recommendations for Intervention Trials (SPIRIT) 2013 statement[32]. The statistical aspects of the pilot study are summarised here with details fully described in a Statistical Analysis Plan (SAP) that will be available before the recruitment of the last participant.

For peer review only - http://bmjopen.bmj.com/site/about/guidelines.xhtml

2.1 Study design

The DoBAt study is a two-arm single-blind individual-level randomised controlled pilot feasibility trial. A total of 200 adolescents will be recruited (1:1 allocation ratio).

2.2 Study Setting

The study will be based in the Bushbuckridge sub-district of Mpumalanga Province, South Africa. Whilst South Africa is classified as a middle-income country, large inequalities exist, and the study setting represents a rural area characterised by socioeconomic disadvantage and high rates of youth unemployment. Since 1992 the MRC/Wits Rural Public Health and Health Transitions Research Unit (MRC/Wits-Agincourt) has collected population data, with vital events (pregnancy outcome, deaths, in- and out-migration) and household composition updated annually through its Health and socio-Demographic Surveillance System (HDSS). The total population under surveillance is currently ~116,000 inhabitants residing in 31 contiguous villages[33]. Recruitment will take place at schools within the study site. Notably, a previous study amongst adolescent females in this setting reported a prevalence of depressive symptoms of 18.2%[34] and yet access to treatment is severely limited. Similar data for males is not available.

2.3 Patient and Public Involvement

Adolescents and members of the public have been involved at several stages of the study. The intervention was developed through extensive formative research and user-centred design with adolescents in the study area. We also conducted participatory workshops with adolescents and met with local educators, healthcare workers, and relevant non-governmental organisations to obtain their input on trial methods, including recruitment and risk management strategies. We will seek the involvement of adolescents and members of the public in developing appropriate methods to disseminate study findings.

2.4 Eligibility criteria

To be eligible for inclusion in the pilot trial participants must: 1) be between 15 and 19 years of age and in grades 9 to 11 at the beginning of the study; 2) have symptoms of mild to moderately-severe depression indicated by a score between 5 and 19 on the Patient Health Questionnaire Adolescent Version (PHQ-A); 3) be able to read sufficiently in the local language (Xitsonga) to use the Kuamsha app; 4) intend to continue living in the study site for 12-weeks after the baseline assessment; and 5) provide written informed assent/consent to participate in the study, as well as parent/guardian consent if younger than 18 years.

Participants will be excluded if they: 1) have symptoms of severe depression as indicated by a score of >19 on the PHQ-A; 2) have current suicidal ideation with specific plans and means identified; 3) are receiving psychological treatment for a mental health condition at the time of enrolment; 4) have been hospitalised for at least five days for a severe psychiatric illness (specifically Bipolar Mood Disorder, Schizophrenia or other Psychotic Disorders) or life-

For peer review only - http://bmjopen.bmj.com/site/about/guidelines.xhtml

threatening or other serious medical illness; 5) have a history of Bipolar Mood Disorder, Schizophrenia or other Psychotic Disorders; 6) lack capacity to consent to treatment or research participation or to use the app. Participants excluded for points 1) and 2) will be assessed by the Risk Management Team and referred to local clinical services as per prior arrangements with local service providers.

2.5 Intervention

All participants in the intervention and control arms will be given the entry-level Samsung Galaxy A2 Core Android smartphone, which they can keep at the end of the study. Furthermore, participants in both groups will receive 200MB of mobile internet data at six different time points (0; 2.5; 5; 7.5; 11 and 24 weeks) to ensure they have data to use the app and complete the online surveys. Participants in both arms will receive active symptom monitoring via text messages sent to the smartphone every 2.5 weeks. Any adolescents who develop severe depression or high-risk suicidal ideation will be assessed by the Risk Management Team (further details provided in Table 2 of the supplementary materials) and referred to local clinical services. Adolescents started on anti-depressant medication or who receive psychological therapy because of these referrals will not be discontinued, but we will take note of concomitant care and will examine this using sensitivity analyses.

Control arm: Enhanced Standard of Care
The non-intervention arm will receive a control app (the *Kuchunguza app*) containing six video clips from *WildEarth-SafariLive*, a locally produced wildlife series. Each video clip takes approximately 15-20 minutes to complete and allows users to explore the African wilderness while listening to calming and atmospheric sounds. In the given context, the control represents an enhancement of standard care since most adolescents with depression would not usually receive any intervention or active symptom monitoring and referral.

Intervention arm: Kuamsha programme
The intervention arm will receive six modules of Behavioural Activation (BA) therapy via a smartphone application (the *Kuamsha app*) supported by trained Peer Mentors, implemented over 10 weeks. The app and phone calls from the Peer Mentors together comprise the Kuamsha programme.

The Kuamsha app is primarily an interactive narrative game consisting of six tailored modules (sessions) containing Behavioural Activation's core principles integrated into the gamified story content format[35]. Each module takes approximately 15-20 mins to complete, and they cover topics such as identifying and engaging in meaningful activities and using strategies to overcome barriers, for example, using problem-solving, effective communication, getting enough sleep, and disengaging from rumination. Each module is followed by a homework activity where the participant is encouraged to reflect on the BA principles outlined in each module and think about ways to apply the principles to their own lives. Participants will be asked to report often they completed the homework activities and their mood as they were doing these activities. The app includes game design elements to stimulate motivation and performance, including character personification, in-app points, and reminders/notifications. Example mock-ups of the Kuamsha app and a summary of each module are shown in Tables 4-6 in the supplemental materials.

For peer review only - http://bmjopen.bmj.com/site/about/guidelines.xhtml

The Kuamsha app will be supplemented by weekly phone calls (15 mins per module) from Trained Peer Mentors. There will be seven calls in total, including one introductory phone call and six calls to cover module content. Peer Mentors will attempt to reach participants by calling up to 5 times per week. The role of the Peer Mentors is mainly to support adherence and compliance with the app, troubleshoot problems related to the use of the app, and assist with the implementation of the homework activities. They will be trained not to provide additional advice or counselling and conduct their calls according to a pre-determined checklist of activities to help ensure fidelity. Peer Mentors will be Xitsonga-speaking students or recent graduates from the department of psychology or social work at an accredited South African university. The Trial Psychologist will train and supervise them according to the training manual developed specifically for the intervention.

2.6 Primary objectives and outcome measures

(1) Feasibility and acceptability of the intervention. A mixed-methods approach will be adopted to establish feasibility and acceptability.

- **Feasibility** will be assessed by collecting data on the following:
  (1) Recruitment (enrolment rate of eligible participants) and retention in the trial at the end of the intervention period (11 weeks).
  (2) Feasibility of testing procedures and data collection methods, including assessment of completion rates.
  (3) Treatment adherence rates, where adherence is defined as having opened at least 4 out of 6 of the app modules and as having completed 3 out of 6 phone calls with the peer mentor (excluding the introductory call). We will complement the treatment adherence rates with engagement metrics collected via the app (number of times participants logged into the app, number of modules opened and completed, total time spent on the app, number of weekly activities set to do, and number of times the participant completed the weekly activities).
- **Acceptability** of the intervention and study procedures will be assessed via:
  (1) An acceptability questionnaire conducted at the end of intervention assessment (week 11) with all participants. The questionnaire consists of three measures: Acceptability of Intervention Measure (AIM), Intervention Appropriateness Measure (IAM), and Feasibility of Intervention Measure (FIM)[36]. Each measure consists of 4 items. The total score ranges from 1 to 5 and is calculated by averaging response scores to the response categories. We will calculate an average score for each of the measures. We will inquire about the acceptability of the app using these three measures (to both the intervention and control groups) and the acceptability of the peer mentor programme (to the intervention group only).
  (2) In-depth interviews with a subsample of participants. We estimate that interviews with 20 participants will provide a sufficient range of experiences and perspectives to reach data saturation. This subsample will contain participants from the treatment and control arms and will be stratified based on high versus low app engagement.
- ***Fidelity of delivery of the intervention*** will be assessed by collecting data on adherence and competence of Trained Peer Mentors.

For peer review only - http://bmjopen.bmj.com/site/about/guidelines.xhtml

(1) Adherence of the peer mentors is defined as the number of sessions that meet at least 90% of the criteria for adherence according to the training protocol. Independent raters will listen to a random sample of recordings of Peer Mentors' phone calls with participants and assess them against the training protocol.
(2) Competence of the peer mentors will be expressed as a percentage based on their Competency Assessment Test. This test will include a written test and observation of skills through role-playing to assess Knowledge, Attitudes and Practices. Tests will be scored by the Trial Psychologist using a pre-determined scoring system.

Trial progression criteria

Feasibility of the intervention and trial procedures and progression criteria for a definitive RCT will be based on a traffic light system of green (continue to RCT), amber (make modifications to trial procedures before embarking on a definitive RCT), and red (a definitive RCT is unlikely to be feasible)[37].

We will evaluate the following:

**Criteria**	**Green**	**Amber**	**Red**
Enrolment (recruitment) rate of eligible participants	≥60%	<60%, ≥40%	<40%
Retention in the trial at 11 weeks	≥90%	<90%, ≥50%	<50%
Proportion of participants that open at least 4 out of 6 of the app modules	≥70%	<70%, ≥50%	<50%
Proportion of participants that have 3 out of 6 phone calls with the peer mentor	≥70%	<70%, ≥50%	<50%

(2) Signals of Initial Efficacy on depressive symptoms.
This will be assessed using the PHQ-A score at the end of intervention assessment (week 11). The PHQ-A is a widely used and well-established measure of adolescent depressive symptoms over the past two weeks[38]. This measure showed good psychometric properties with a sample of adolescents and young adults in South Africa and Kenya [39–41]. The questionnaire consists of 9 items. The PHQ-A total score ranges from 0 to 27 and is calculated by assigning scores to the response categories [0=not at all, 1=several days, 2=more than half the days, 3-nearly every day] and summing the score for each of the items. Higher scores indicate greater severity of depression. We will use this scale to assess depressive symptoms at screening, week 5, week 11, and week 24. Participants will also be asked to complete this scale at week 2.5 and week 7.5 as part of the symptom monitoring. More information on how we will analyse this data and trial progression criteria can be found in the data analysis section.

2.7 Secondary objectives and outcome measures

For peer review only - http://bmjopen.bmj.com/site/about/guidelines.xhtml

As part of our secondary objectives, we will pilot locally adapted measures of mental health, social-affective and cognitive processing, risky behaviours, and socioeconomic outcomes and collect descriptive data to inform the development of a future larger trial.

(i) *Mental health outcomes:* include the following scales Generalised Anxiety Disorder (GAD-7)[42]; Connor-Davidson Resilience Scale (CD-RISC) [43]; Brief Rumination Response Scale (RRS) [44]; Warwick-Edinburgh Mental Wellbeing Scale (WEMWBS)[45]; Behavioural Activation for Depression Scale (BADS)[46]. Participants will be asked to complete these scales at baseline (week 0), mid-intervention (week 5), end of intervention (week 11), and follow-up (week 24). See Table 1 for details.

(ii) Social-affective and cognitive processing: include five computerised cognitive tasks measuring affective set-shifting, affective working memory, risk-seeking preferences, emotion recognition, and abstract reasoning. The tasks include a modified version of the Wisconsin Card Sorting Task[47]; the Backward Digit Span Task[48]; the Balloon Analogue Risk Task (BART)[49]; an Emotion Recognition Task[50]; and the Matrix Reasoning Item Bank (MaRs-IB) Task[51]. Participants will be asked to complete these tasks at baseline (week 0) and end of intervention (week 11).

(iii) Risky behaviours: The questions on sexual behaviours include a subset of items from the Wits Reproductive Health and HIV Institute (Wits RHI) sexual behaviour survey[52]. We will measure substance use using the Alcohol, Smoking and Substance Involvement Screening Test for Youth (ASSIST-Y)[53]. We will also ask about delinquency, gambling, and peer influence in participants' engagement with risky behaviours. Participants will be asked to complete these tasks at baseline (week 0) and end of intervention (week 11).

(iv) Socioeconomics: We will measure economic preferences (time preference, risk preference, and loss aversion) using three incentivised tasks[54,55]. In addition, we will measure a range of socioeconomic outcomes, including measures of time use, human capital investment, and spending. Participants will be asked to complete these tasks at baseline (week 0) and end of intervention (week 11).

2.8 Recruitment and participant timeline

Participants will be recruited through a two-stage recruitment process. The first stage of recruitment (Phase 1) will consist of a screening survey conducted in schools to identify adolescents with symptoms of mild to moderately-severe depression. The second stage of recruitment (Phase 2) will be done with adolescents who score between 5 and 19 on the PHQ-A in the school survey and who also meet the other eligibility criteria.

The pilot trial consists of an 11-week intervention treatment phase, and participants will be followed up for a further 13 weeks after completing the intervention (i.e., a total of 24 weeks). Primary outcomes will be assessed at the end of intervention assessment (week 11) by blinded outcomes assessors. Table 1 indicates the time schedule of enrolment, interventions and assessments for participants, and Figure 1 shows the flow of participants.

For peer review only - http://bmjopen.bmj.com/site/about/guidelines.xhtml

Table 1. Schedule of enrolment, interventions, and assessments for participants

	STUDY PERIOD							
Assessment	Enrolment	Allocation	Post-allocation					Close-out
TIME POINT (in weeks)	$-t_1$	0	1	2.5	5	7.5	11	24
ENROLMENT								
Eligibility screen (school survey)	X							
Informed consent	X							
Allocation		X						
Phone delivery		X						
INTERVENTION								
Intervention arm: Kuamsha app & Peer Mentor calls			●	—	—	—	●	
Control arm: Kuchunguza app			●	—	—	—	●	
ASSESSMENTS								
School survey: PHQ-A, sociodemographic questions & eligibility checklist	X							
Baseline assessment: demographic questionnaire, GAD-7, CD-RISC, RRS, WEMWBS, BADS, five social-affective and cognition tasks, risky behaviours, and socioeconomic outcomes	X							
Symptom monitoring: PHQ-A				X		X		
Mid-intervention assessment: PHQ-A, GAD-7, RRS, WEMWBS short form, BADS (activation & social impairment subscales)					X			
End of intervention assessment: same as baseline + PHQ-A + acceptability questionnaire & IDIs with subsample							X	
Follow-up assessment: PHQ-A + GAD-7, RRS, BADS (activation subscale), WEMWBS short form								X

2.9 Allocation and blinding

Participants will be randomly assigned to the intervention or control arm with a 1:1 allocation using a computerised minimisation algorithm, balanced by sex (male or female) and severity of depressive symptoms (<10 or ≥ 10 on the PHQ-A). The minimisation algorithm was generated by the Centre for Healthcare Randomised Trials (CHaRT) at the University of Aberdeen. Participants will be allocated using CHaRT's online software and the Trial Manager will oversee the enrolment of participants as per assignment.

For peer review only

For peer review only - http://bmjopen.bmj.com/site/about/guidelines.xhtml

For practical and risk management reasons the Trial Manager, Trial Psychologist, Peer Mentors, and Fieldworkers delivering the phones to participants and receiving calls to troubleshoot problems with the app/ phone (i.e., "Phones Team") will not be blinded. However, all fieldworkers conducting outcomes assessments (i.e., the "Assessments Team") will be blinded to participants' allocation status. Any breaches in blinding will be documented, and we will ensure to change the fieldworker conducting subsequent assessments on a particular adolescent where unblinding has occurred.

2.10 Analysis and statistical methods

Quantitative data will be analysed using *Stata* Version 14.0[56], R[57] and other appropriate statistical analysis packages under the direction of the trial statistician.

Participants will be analysed in the groups to which they are randomly assigned, regardless of deviation from the protocol or treatment received (intention to treat population), and the data analyst will be blind to arm allocation. We will use descriptives to explore patterns in the data, followed by inferential statistics involving univariate and multivariable models. A two-tailed p-value <0.05 will be considered statistically significant in the inferential analyses.

Primary objectives

Our first co-primary objective (feasibility and acceptability) will be assessed using a mixed-methods approach. We will compute appropriate summary statistics (e.g., proportions, means, SDs, etc.) for each quantitative outcome and evaluate the feasibility of the trial based on the pre-defined progression criteria discussed earlier.

Qualitative interviews will be audio-recorded, transcribed verbatim and translated into English. Transcripts will be analysed using thematic analysis. We will follow Braun and Clarke's six phases of analysis (i.e. becoming familiar with the data, generating initial codes, searching for themes, reviewing themes, defining and naming themes, and producing the report)[58]. Coding will be done by two independent researchers. NVivo10, a computer program that aids in the sorting and management of qualitative data, will be used to facilitate the analysis.

Our second co-primary objective (initial efficacy on depressive symptoms) will be based on the PHQ-A score (continuous) at 11 weeks. Outcomes will be compared between intervention and control groups using a linear regression model and adjusting for covariates (PHQ score measured at baseline, sex, age, depression severity, and household asset index). As a secondary analysis, we will make use of the repeated measurements of the PHQ-A throughout the trial (up to 6 times per individual) to evaluate the treatment effects over time. The details of this analysis will be documented in the SAP.

Secondary objectives

The descriptive data of the secondary outcomes will include possible floor and ceiling effects, accuracy, duration, latency, and efficiency. We will perform standard psychometric tests to examine whether the instruments perform well in the study context (e.g., reliability, validity,

For peer review only - http://bmjopen.bmj.com/site/about/guidelines.xhtml

acceptability) and calculate bivariate correlations across variables of interest. We anticipate that some secondary analyses will be included in companion papers rather than the main paper. The details of this analysis will be documented in a separate analysis plan.

2.11 Sample size considerations

Statistical power was calculated to detect differences between two independent groups, in a two-sided test with an α of 0.05 and a power of 1–β=0.80, for an effect size of 0.45. This effect size is based on findings from previous studies investigating digital psychological interventions, with effect size (Cohen's d) ranging from 0.24 to 0.57[59–61]. Given these findings, we chose a small-to-medium effect size of 0.45 to account for the limited number of studies conducted with our target population and in our study setting. We allowed for 25% attrition, based on a previous study with adolescents in the Agincourt setting[62]. Given these assumptions, we aimed to recruit 200 participants at baseline. We calculated the required sample size using G*Power software, version 3.1.9.3[63].

3. ETHICS AND DISSEMINATION

3.1 Research Ethics Approval

Ethical approval for this study has been obtained from the University of the Witwatersrand Human Research Ethics Committee (MED20-05-011), Ehlanzeni District and Mpumalanga Provincial Departments of Health and Education, and the Oxford Tropical Research Ethics Committee (OxTREC 34-20). This trial was registered with the South African National Clinical Trials Registry (DOH-27-112020-5741) and the Pan African Clinical Trials Registry (PACTR202206574814636) in November 2020.

3.2 Informed Consent

At both stages of recruitment, we will obtain informed consent from participants aged 18 or over and informed assent and parental/guardian consent from participants younger than 18. All information sheets will be available in Xitsonga and English, and assent/consent will be obtained by trained and supervised bilingual fieldworkers.

3.3 Confidentiality and management of participant data

The confidentiality of participants' data and information will be respected and maintained by all study staff. Staff members will be trained accordingly and required to sign a non-disclosure agreement. A unique participant identification number will be used to link study data and information. All electronic data will be stored in a secure, protected and access-controlled database at the Agincourt data centre. Paper-based documentation will be stored in a locked cabinet and only accessible to authorised staff. Confidentiality may be broken in instances of immediate harm to self or others, as detailed in consent and assent forms.

3.4 Harms/ Anticipated risks

A comprehensive Risk Management Protocol has been developed to ensure the safety of participants in the trial. Participants with severe depression, suicidal ideation and other risks will receive a telephonic and/or in-person assessment by a member of the Risk Management Team.

For peer review only - http://bmjopen.bmj.com/site/about/guidelines.xhtml
For peer review only

Subsequently, appropriate referrals and linkage to care will be made using established networks with local providers. Details of the independent Data and Safety Monitoring Board (DSMB) and Trial Steering Committee (TSC) are provided in Table 2 of the supplementary materials. Serious Adverse Events will be reported to the DSMB chair within 48 hours and to both Ethics Committees within seven days. Summary tables of all Adverse Events will be sent to the DSMB on a quarterly basis and to the Ethics Committees on an annual basis.

3.5 Publication and dissemination

Results of the trial will be communicated to participants, the public, researchers, healthcare professionals, and policymakers. We will seek the involvement of adolescents and members of the public in the development of appropriate methods to disseminate study findings. Policymakers and other key stakeholders within provincial and national departments of health, education, and social development, as well as relevant local NGOs, will be engaged in dialogue and supplied with a technical brief to convey the results of the trial and their implications for policy and practice. Trial results will be published as soon as possible after completion, and ensuing publications will be made open access. Results will also be presented at relevant national and international conferences. Authorship will be determined in accordance with the ICMJE guidelines, and other contributors will be acknowledged.

Acknowledgements

We would like to acknowledge the Field Supervisors Meriam Meritze, Princess Makhubela and Nokthula Mayindi, as well as all the Fieldworkers, Registered Counsellor, Tinyiko Mafumo, and Peer Mentors for their hard work and dedication. We would also like to thank all the adolescents who were involved in the formative work and participatory workshops that were integral to the design and development of the intervention, as well as the educators, healthcare workers and non-governmental organisations that provided valuable input into the trial design, particularly regarding recruitment and risk management strategies.

Funding Statement

Research reported in this publication was supported by the South African Medical Research Council with funds received from the South African National Department of Health and the UK Medical Research Council, with funds received from the UK Government's Newton Fund (grant number MR/S008748/1). It also received financial support from the NIHR Oxford Health Biomedical Research Centre (grant number: N/A). The study was conducted within the MRC/Wits Rural Public Health and Health Transitions Research Unit and Agincourt Health and socio-Demographic Surveillance System, a node of the South African Population Research Infrastructure Network (SAPRIN), supported by the Department of Science and Innovation, the University of the Witwatersrand, and the Medical Research Council, South Africa (grant number: N/A). SJB is funded by Wellcome (grant number WT107496/Z/15/Z), the MRC-UK, the Jacobs Foundation, the Wellspring Foundation, and the University of Cambridge (grant number: N/A). JRP is funded by Fundación Rafael del Pino and received funding support from MRC UK and the Wellspring Philanthropic Fund (grant number: N/A). EJK received funding support from a UKRI-GCRF Impact Acceleration Award (grant number: N/A). Study funders played no role in the study design, collection, management, analysis or interpretation of data, the writing of this report, or the decision to submit it for publication.

For peer review only - http://bmjopen.bmj.com/site/about/guidelines.xhtml

Competing Interests

The authors declare no competing interests.

Authors' contributions

The first draft of the manuscript was written by BM and JRP. All authors provided substantial contributions to the conception and design of the study and gave the final approval of the version to be published. Affiliations and contact details for all authors are provided in Table 1 of the Supplementary Materials.

REFERENCES

1 Kieling C, Baker-Henningham H, Belfer M, *et al.* Child and adolescent mental health worldwide: evidence for action. *Lancet* 2011;**378**:1515–25. doi:10.1016/S0140-6736(11)60827-1

2 Erskine HE, Moffitt TE, Copeland WE, *et al.* A heavy burden on young minds: the global burden of mental and substance use disorders in children and youth. *Psychol Med* 2015;**45**:1551. doi:10.1017/S0033291714002888

3 Herrman H, Patel V, Kieling C, *et al.* Time for united action on depression: a Lancet–World Psychiatric Association Commission. *Lancet* 2022;**399**:957–1022. doi:10.1016/S0140-6736(21)02141-3

4 Lund C, De Silva M, Plagerson S, *et al.* Poverty and mental disorders: breaking the cycle in low-income and middle-income countries. *Lancet* 2011;**378**:1502–14. doi:10.1016/S0140-6736(11)60754-X

5 Sawyer SM, Afifi RA, Bearinger LH, *et al.* Adolescence: A foundation for future health. Lancet. 2012;**379**:1630–40. doi:10.1016/S0140-6736(12)60072-5

6 Almlund M, Duckworth AL, Heckman J, *et al.* Personality Psychology and Economics. In: Hanushek EA, Machin SJ, Woessman L, eds. *Handbook of the Economics of Education*. Elsevier 2011. 1–181. doi:10.1016/B978-0-444-53444-6.00001-8

7 Blakemore SJ, Choudhury S. Development of the adolescent brain: implications for executive function and social cognition. *J Child Psychol Psychiatry* 2006;**47**:296–312. doi:10.1111/J.1469-7610.2006.01611.X

8 Sheehan P, Sweeny K, Rasmussen B, *et al.* Building the foundations for sustainable development: a case for global investment in the capabilities of adolescents. *Lancet* 2017;**390**:1792–806. doi:10.1016/S0140-6736(17)30872-3

9 Kieling C, Adewuya A, Fisher HL, *et al.* Identifying depression early in adolescence. *Lancet Child Adolesc Heal* 2019;**3**:211. doi:10.1016/S2352-4642(19)30059-8

10 Thapar A, Collishaw S, Pine DS, *et al.* Depression in adolescence. Lancet. 2012;**379**:1056–67. doi:10.1016/S0140-6736(11)60871-4

11 Snyder HR. Major depressive disorder is associated with broad impairments on neuropsychological measures of executive function: A meta-analysis and review. *Psychol Bull* 2013;**139**:81–132. doi:10.1037/a0028727

12 Weightman MJ, Air TM, Baune BT. A review of the role of social cognition in major depressive disorder. Front. Psychiatry. 2014;**5**:179. doi:10.3389/fpsyt.2014.00179

13 Pozuelo JR, Desborough L, Stein A, *et al.* Systematic Review and Meta-Analysis: Depressive Symptoms and Risky Behaviours among Adolescents in Low- and Middle-

For peer review only - http://bmjopen.bmj.com/site/about/guidelines.xhtml

Income Countries. *J Am Acad Child Adolesc Psychiatry* Published Online First: 2021. doi:10.1016/j.jaac.2021.05.005
14 Lund C, Myer L, Stein DJ, *et al.* Mental illness and lost income among adult South Africans. *Soc Psychiatry Psychiatr Epidemiol* 2013;**48**:845–51. doi:10.1007/s00127-012-0587-5
15 Das JK, Salam RA, Lassi ZS, *et al.* Interventions for Adolescent Mental Health: An Overview of Systematic Reviews. *J Adolesc Heal* 2016;**59**:S49. doi:10.1016/J.JADOHEALTH.2016.06.020
16 Klasen H, Crombag AC. What works where? A systematic review of child and adolescent mental health interventions for low and middle income countries. *Soc Psychiatry Psychiatr Epidemiol* 2013;**48**:595–611. doi:10.1007/S00127-012-0566-X/TABLES/3
17 Kakuma R, Minas H, Van Ginneken N, *et al.* Human resources for mental health care: current situation and strategies for action. *Lancet* 2011;**378**:1654–63. doi:10.1016/S0140-6736(11)61093-3
18 Kemp CG, Petersen I, Bhana A, *et al.* Supervision of Task-Shared Mental Health Care in Low-Resource Settings: A Commentary on Programmatic Experience. *Glob Heal Sci Pract* 2019;**7**:150–9. doi:10.9745/GHSP-D-18-00337
19 Fekadu A, Demissie M, Birhane R, *et al.* Under detection of depression in primary care settings in low and middle-income countries: a systematic review and meta-analysis. *Syst Rev* 2022;**11**:1–10. doi:10.1186/S13643-022-01893-9/FIGURES/3
20 Stunden C, Zasada J, VanHeerwaarden N, *et al.* Help-Seeking Behaviors of Transition-Aged Youth for Mental Health Concerns: Qualitative Study. *J Med Internet Res* 2020;**22**:e18514. doi:10.2196/18514
21 Richards DA, Ekers D, McMillan D, *et al.* Cost and Outcome of Behavioural Activation versus Cognitive Behavioural Therapy for Depression (COBRA): a randomised, controlled, non-inferiority trial. *Lancet* 2016;**388**:871–80. doi:10.1016/S0140-6736(16)31140-0
22 Ekers D, Webster L, Van Straten A, *et al.* Behavioural activation for depression; An update of meta-analysis of effectiveness and sub group analysis. *PLoS One* 2014;**9**:e100100. doi:10.1371/journal.pone.0100100
23 Tindall L, Mikocka-Walus A, McMillan D, *et al.* Is behavioural activation effective in the treatment of depression in young people? A systematic review and meta-analysis. *Psychol Psychother Theory, Res Pract* 2017;**90**:770–96. doi:10.1111/PAPT.12121
24 Martin F, Oliver T. Behavioral activation for children and adolescents: a systematic review of progress and promise. *Eur Child Adolesc Psychiatry* 2019;**28**:427–41. doi:10.1007/S00787-018-1126-Z/FIGURES/2
25 Jacob M, L. Keeley M, Ritschel L, *et al.* Behavioural activation for the treatment of low-income, African American adolescents with major depressive disorder: a case series. *Clin Psychol Psychother* 2013;**20**:87–96. doi:10.1002/CPP.774
26 Patel V, Weobong B, Weiss HA, *et al.* The Healthy Activity Program (HAP), a lay counsellor-delivered brief psychological treatment for severe depression, in primary care in India: a randomised controlled trial. *Lancet* 2017;**389**:176–85. doi:10.1016/S0140-6736(16)31589-6
27 Lehmann DC, Bördlein C. A Systematic Review of Culturally Adapted Behavioral Activation Treatments for Depression: *Res Soc Work Pract* 2020;**30**:688–702. doi:10.1177/1049731520915635

For peer review only

28 Huguet A, Miller A, Kisely S, *et al.* A systematic review and meta-analysis on the efficacy of Internet-delivered behavioral activation. *J Affect Disord* 2018;**1**:27–38.
29 Naslund JA, Aschbrenner KA, Araya R, *et al.* Digital technology for treating and preventing mental disorders in low-income and middle-income countries: a narrative review of the literature. *The Lancet Psychiatry* 2017;**4**:486–500. doi:10.1016/S2215-0366(17)30096-2
30 Lehtimaki S, Martic J, Wahl B, *et al.* Evidence on Digital Mental Health Interventions for Adolescents and Young People: Systematic Overview. *JMIR Ment Heal* 2021;**8**. doi:10.2196/25847
31 Linardon J, Fuller-Tyszkiewicz M. Attrition and adherence in smartphone-delivered interventions for mental health problems: A systematic and meta-analytic review. *J Consult Clin Psychol* 2020;**88**:1–13. doi:10.1037/CCP0000459
32 Chan AW, Tetzlaff JM, Altman DG, *et al.* SPIRIT 2013 statement: Defining standard protocol items for clinical trials. *Ann Intern Med* 2013;**158**:200–7. doi:10.7326/0003-4819-158-3-201302050-00583
33 Kahn K, Collinson MA, Gómez-Olivé FX, *et al.* Profile: Agincourt health and socio-demographic surveillance system. *Int J Epidemiol* 2012;**41**:988–1001. doi:10.1093/ije/dys115
34 Goin DE, Pearson RM, Craske MG, *et al.* Depression and incident HIV in adolescent girls and young women in HPTN 068: Targets for prevention and mediating factors. *Am J Epidemiol* Published Online First: 29 October 2019. doi:10.1093/aje/kwz238
35 Jacobson NS, Martell CR, Dimidjian S. Behavioral Activation Treatment for Depression: Returning to Contextual Roots. *Clin Psychol Sci Pract* 2006;**8**:255–70. doi:10.1093/clipsy.8.3.255
36 Weiner BJ, Lewis CC, Stanick C, *et al.* Psychometric assessment of three newly developed implementation outcome measures. *Implement Sci* 2017;**12**:1–12. doi:10.1186/S13012-017-0635-3/TABLES/3
37 Avery KNL, Williamson PR, Gamble C, *et al.* Informing efficient randomised controlled trials: exploration of challenges in developing progression criteria for internal pilot studies. *BMJ Open* 2017;**7**:e013537. doi:10.1136/BMJOPEN-2016-013537
38 Kroenke K, Spitzer RL, Williams JBW. The PHQ-9: Validity of a brief depression severity measure. *J Gen Intern Med* Published Online First: 2001. doi:10.1046/j.1525-1497.2001.016009606.x
39 Bhana A, Rathod SD, Selohilwe O, *et al.* The validity of the Patient Health Questionnaire for screening depression in chronic care patients in primary health care in South Africa. *BMC Psychiatry* 2015;**15**:118. doi:10.1186/s12888-015-0503-0
40 Cholera R, Gaynes BN, Pence BW, *et al.* Validity of the patient health questionnaire-9 to screen for depression in a high-HIV burden primary healthcare clinic in Johannesburg, South Africa. *J Affect Disord* 2014;**167**:160–6. doi:10.1016/j.jad.2014.06.003
41 Osborn TL, Venturo-Conerly KE, Wasil AR, *et al.* Depression and Anxiety Symptoms, Social Support, and Demographic Factors Among Kenyan High School Students. *J Child Fam Stud* 2019;:1–12. doi:10.1007/s10826-019-01646-8
42 Spitzer RL, Kroenke K, Williams JBW, *et al.* A brief measure for assessing generalized anxiety disorder: the GAD-7. *Arch Intern Med* 2006;**166**:1092–7. doi:10.1001/ARCHINTE.166.10.1092
43 Connor KM, Davidson JRT. Development of a new resilience scale: the Connor-Davidson

For peer review only

For peer review only - http://bmjopen.bmj.com/site/about/guidelines.xhtml

Resilience Scale (CD-RISC). *Depress Anxiety* 2003;**18**:76–82. doi:10.1002/DA.10113
44 Treynor W, Gonzalez R, Nolen-Hoeksema S. Rumination reconsidered: A psychometric analysis. *Cognit Ther Res* 2003;**27**:247–59. doi:10.1023/A:1023910315561
45 Tennant R, Hiller L, Fishwick R, *et al.* The Warwick-Dinburgh mental well-being scale (WEMWBS): Development and UK validation. *Health Qual Life Outcomes* 2007;**5**:1–13. doi:10.1186/1477-7525-5-63/TABLES/4
46 Kanter JW, Mulick PS, Busch AM, *et al.* The Behavioral Activation for Depression Scale (BADS): Psychometric properties and factor structure. *J Psychopathol Behav Assess* 2007;**29**:191–202. doi:10.1007/S10862-006-9038-5
47 Schweizer S, Parker J, Leung JT, *et al.* Age-related differences in affective control and its association with mental health difficulties. *cambridge.org* Published Online First: 2019. doi:10.1017/S0954579419000099
48 Jahanshahi M, Saleem T, Ho AK, *et al.* A preliminary investigation of the running digit span as a test of working memory. *Behav Neurol* 2008;**20**:17–25. doi:10.3233/BEN-2008-0212
49 White TL, Lejuez CW, de Wit H. Test-Retest Characteristics of the Balloon Analogue Risk Task (BART). *Exp Clin Psychopharmacol* 2008;**16**:565–70. doi:10.1037/a0014083
50 Fuhrmann D, Knoll LJ, Sakhardande AL, *et al.* Perception and recognition of faces in adolescence. *Sci Rep* 2016;**6**:33497. doi:10.1038/srep33497
51 Chierchia G, Fuhrmann D, Knoll LJ, *et al.* The matrix reasoning item bank (MaRs-IB): novel, open-access abstract reasoning items for adolescents and adults. *royalsocietypublishing.org* 2019;**6**. doi:10.1098/rsos.190232
52 Pettifor A, Rees H, Steffenson A, *et al.* HIV and sexual behaviour among young South Africans: a national survey of 15-24 year olds. *Reprod Heal Res Unit, Univ Witswatersrand* 2004.
53 Humeniuk R, Holmwood C, Beshara M, *et al.* ASSIST-Y V1.0: First-Stage Development of the WHO Alcohol, Smoking and Substance Involvement Screening Test (ASSIST) and Linked Brief Intervention for Young People. *J Child Adolesc Subst Abuse* 2016;**25**:384–90. doi:10.1080/1067828X.2015.1049395
54 Andersen S, Harrison GW, Lau MI, *et al.* Elicitation using multiple price list formats. *Exp Econ* 2006;**9**:383–405. doi:10.1007/s10683-006-7055-6
55 Kahneman D, Tversky A. Prospect theory: An analysis of decision under risk. In: *Experiments in Environmental Economics*. Taylor and Francis Inc. 2018. 143–72. doi:10.2307/1914185
56 StataCorp. Stata Statistical Software: Release 14. 2015.
57 Team RC. R: A Language and Environment for Statistical Computing. 2021.
58 Braun V, Clarke V. Using thematic analysis in psychology. *Qual Res Psychol* 2006;**3**:77–101. doi:10.1191/1478088706qp063oa
59 Arjadi R, Nauta MH, Scholte WF, *et al.* Internet-based behavioural activation with lay counsellor support versus online minimal psychoeducation without support for treatment of depression: a randomised controlled trial in Indonesia. *The Lancet Psychiatry* 2018;**5**:707–16. doi:10.1016/S2215-0366(18)30223-2
60 Lehtimaki S, Martic J, Wahl B, *et al.* Evidence on Digital Mental Health Interventions for Adolescents and Young People: Systematic Overview. 2021;**8**:e25847. doi:10.2196/25847
61 Fu Z, Burger H, Arjadi R, *et al.* Effectiveness of digital psychological interventions for mental health problems in low-income and middle-income countries: a systematic review

For peer review only - http://bmjopen.bmj.com/site/about/guidelines.xhtml

and meta-analysis. *The Lancet Psychiatry* 2020;**7**:851–64. doi:10.1016/S2215-0366(20)30256-X

62 Pettifor A, MacPhail C, Selin A, *et al.* HPTN 068: A Randomized Control Trial of a Conditional Cash Transfer to Reduce HIV Infection in Young Women in South Africa—Study Design and Baseline Results. *AIDS Behav* 2016;**20**:1863–82. doi:10.1007/S10461-015-1270-0/FIGURES/3

63 Faul F, Erdfelder E, Lang AG, *et al.* G*Power 3: A flexible statistical power analysis program for the social, behavioral, and biomedical sciences. *Behav Res Methods 2007 392* 2007;**39**:175–91. doi:10.3758/BF03193146

Figure 1
CONSORT Flow Diagram for the DoBAt Pilot Trial

For peer review only

For peer review only - http://bmjopen.bmj.com/site/about/guidelines.xhtml

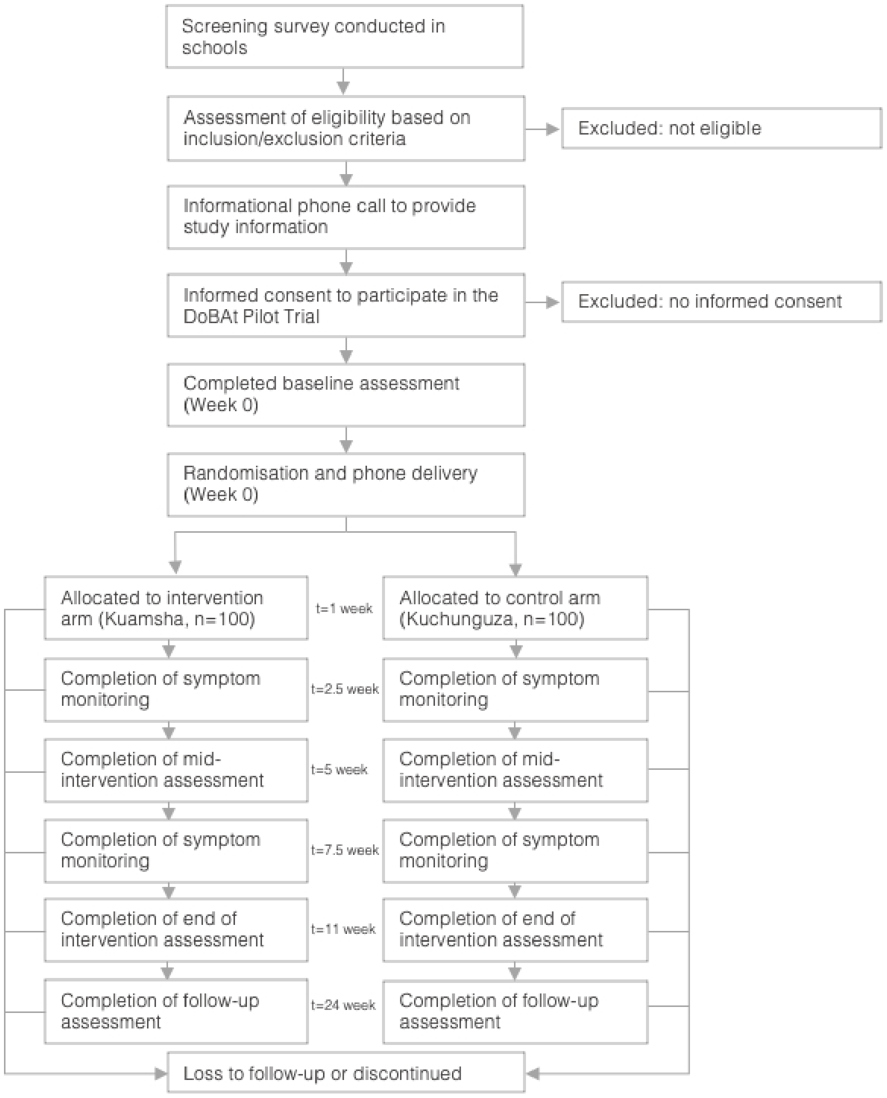

Figure 1. CONSORT Flow Diagram for the DoBAt Pilot Trial

232x263mm (72 x 72 DPI)

SUPPLEMENTARY MATERIALS

Table 1. Investigators' affiliations and contact details

Name	Role	Affiliation	Email
**Prof. Kathleen Kahn**	Co-Principal Investigator	Professor of Public Health and Principal Scientist, MRC/Wits Rural Public Health and Heath Transitions Research Unit (Agincourt), School of Public Health, University of the Witwatersrand, Johannesburg, South Africa	Kathleen.Kahn@wits.ac.za
**Prof. Alan Stein**	Co-Principal Investigator	Professor and Head of Section, Child and Adolescent Psychiatry, Department of Child and Adolescent Psychiatry, University of Oxford, Oxford, United Kingdom	Alan.Stein@psych.ox.ac.uk
**Prof. Sarah-Jayne Blakemore**	Investigator	Professor of Psychology and Cognitive Neuroscience, University of Cambridge, Cambridge, United Kingdom	sjblakemore@psychol.cam.ac.uk
**Dr. Gabriele Chierchia**	Investigator	Research and Teaching Associate at the University of Cambridge Department of Psychology, Cambridge, United Kingdom	Gsc34@cam.ac.uk
**Prof. Michelle Craske**	Investigator	Professor of Psychology, Psychiatry and Biobehavioral Sciences at the University of California, Los Angeles (UCLA), United States of America	MCraske@mednet.ucla.edu
**Ms Sophie Luise Fielmann**	Investigator	Doctoral Student, Department of Psychology, University of Cambridge, Cambridge, United Kingdom	slf56@cam.ac.uk
**Dr. Xavier Gómez-Olivé**	Investigator/ Agincourt Research Manager	Research Manager, MRC/Wits Rural Public Health and Heath Transitions Research Unit (Agincourt), School of Public Health, University of the Witwatersrand, Johannesburg, South Africa	f.gomez-olivecasas@wits.ac.za
**Prof. Alastair van Heerden**	Investigator	Associate Professor University of Witwatersrand, and Research Director at Human Sciences Research Council (HSRC), Pietermaritzburg, South Africa	alastair.vanheerden@wits.ac.za
**Dr. Emma Kilford**	Investigator	Postdoctoral research fellow at University College London (UCL) Department of Clinical, Educational and Health Psychology, London, United Kingdom	e.kilford@ucl.ac.uk
**Prof. Crick Lund**	Investigator	Professor of Global Mental Health and Development, Centre for Global Mental Health, Institute of Psychiatry, Psychology and Neuroscience, King's College London, United Kingdom; Honorary Professor of	crick.lund@uct.ac.za

For peer review only - http://bmjopen.bmj.com/site/about/guidelines.xhtml

		Public Mental Health, Alan J Flisher Centre for Public Mental Health, Department of Psychiatry and Mental Health, University of Cape Town, South Africa	
**Prof. Heather O'Mahen**	Investigator	Associate Professor in Perinatal Clinical Psychology, University of Exeter, Exeter, United Kingdom	H.OMahen@exeter.ac.uk
**Mr Daniel Mahlangu**	Investigator	Data Specialist DoBAt Study, MRC/Wits Rural Public Health and Heath Transitions Research Unit (Agincourt), School of Public Health, University of the Witwatersrand, Johannesburg, South Africa	Daniel.Mahlangu@wits.ac.za
**Dr. Mahreen Mahmud**	Investigator	Lecturer (Assistant Professor) in Economics at the University of Exeter, Exeter, United Kingdom	M.Mahmud@exeter.ac.uk
**Ms Zamakhanya Makhanya**	Investigator/ Trial Psychologist	Trial Psychologist DoBAt Study, MRC/Wits Rural Public Health and Heath Transitions Research Unit (Agincourt), School of Public Health, University of the Witwatersrand, Johannesburg, South Africa	Zamakhanya.makhanya@wits.ac.za
**Dr. Bianca Moffett**	Investigator/ Trial Manager	Trial Manager DoBAt Study, MRC/Wits Rural Public Health and Heath Transitions Research Unit (Agincourt), School of Public Health, University of the Witwatersrand, Johannesburg, South Africa	Bianca.moffett@wits.ac.za
**Prof. Eustasius Musenge**	Investigator/ Trial Statistician	Associate Professor of Biostatistics, School of Public Health, University of the Witwatersrand, Johannesburg, South Africa	Eustasius.Musenge@wits.ac.za
**Dr. Kate Orkin**	Investigator	Senior Research Fellow in Behavioural Economics at the Centre for the Study of African Economies, University of Oxford, Oxford, United Kingdom	kate.orkin@bsg.ox.ac.uk

For peer review only

**Ms Julia R Pozuelo**	Investigator	Doctoral student, Department of Psychiatry, and Research Assistant in the Centre for Studies of African Economies, University of Oxford, Oxford, United Kingdom	julia.ruizpozuelo@psych.ox.ac.uk
**Prof. Tholene Sodi**	Investigator	Professor, Department of Psychology at the University of Limpopo, Polokwane, South Africa	Tholene.sodi@ul.ac.za
**Prof. Stephen Tollman**	Investigator	Research Professor and Director, MRC/Wits Rural Public Health and Health Transitions Research Unit (Agincourt), School of Public Health, University of the Witwatersrand, Johannesburg, South Africa	Stephen.Tollman@wits.ac.za
**Prof. Imraan Valodia**	Investigator	Pro Vice-chancellor for Climate, Sustainability and Inequality, and Director of Southern Centre for Inequalities Studies, University of the Witwatersrand, Johannesburg, South Africa	Imraan.valodia@wits.ac.za

For peer review only - http://bmjopen.bmj.com/site/about/guidelines.xhtml

Table 2. Trial Personnel and Committees

Committee	Members and responsibilities
**Trial Management Team (TMT)**	Comprised of the co-Principal Investigators, the Trial Manager, the Trial Psychologist, and other investigators. The TMT is responsible for the day to day running of the trial and meets on a weekly basis.
**Risk Management Team (RMT)**	The RMT consists of the Trial Manager (a Medical Doctor), the Trial Clinical Psychologist, the Trial Registered Counsellor, and a senior Field Supervisor. Supervision and oversight are provided by Prof. Alan Stein (Child and Adolescent Psychiatrist), Prof. Kathleen Kahn (Medical Doctor), Prof. Crick Lund (Clinical Psychologist) and Prof. Tholene Sodi (Clinical Psychologist). The RMT is responsible for assessing and managing all risks amongst screened and enrolled participants.
**Trial Steering Committee (TSC)**	Consists of senior academic clinicians and researchers including Prof. Roz Shafran (chair of the TSC, Professor of Translational Psychology, UCL), Prof. Jonathan Roiser (Professor of Cognitive Neuroscience, UCL), Prof. Soraya Seedat (Professor of Psychiatry, Stellenbosch University), and Prof. Jonathan Levin (Professor of Biostatistics, University of Witwatersrand). The TSC oversees the scientific conduct of the study and meets on a quarterly basis throughout the trial.
**Independent Data and Safety Monitoring Board (DSMB)**	Comprised of Prof. Bonginkosi Chiliza (chair of the DSMB, Chief Specialist and Head of the Department of Psychiatry at the University of KwaZulu-Natal), Dr. Elizabeth George (Statistician, MRC Clinical Trials Unit at University College London), Prof. John Joska (HIV Mental Health Research Unit, Division of Neuropsychiatry, University of Cape Town), and Prof. Marguerite Schneider (Department of Psychiatry and Mental Health, University of Cape Town). The DSMB oversees the conduct and safety of the trial. Meetings will be held before recruitment begins, midway through recruitment, and at the end of the 11-week assessment period. A formal interim analysis will not be conducted.

For peer review only - http://bmjopen.bmj.com/site/about/guidelines.xhtml

For peer review only

Table 3. Trial registration data

Data category	Information
**Primary registry and trial identifying number**	South African National Clinical Trials Registry (DOH-27-112020-5741); Pan African Clinical Trials Registry(PACTR202206574814636)
**Date of registration in primary registry**	19th November 2020
**Ethics reference**	MED20-05-011 / OxTREC 34-20
**Protocol version**	v1.2 19/04/2021
**Protocol amendments**	Important protocol amendments such as changes to eligibility criteria, outcomes or analysis will be reported to investigators, the TSC, and both ethics committees in writing, and relayed to study participants at the soonest availability.
**Funder**	MRC Newton Fund UK-South Africa Joint Initiative on Mental Health (MR/S008748/1)
**Sponsor**	Wits Health Consortium (Pty) Limited, University of Witwatersrand<br>31 Princess of Wales Terrace, Parktown, Johannesburg, 2193<br>011 274 9200
**Trial Title**	Digital delivery of Behavioural Activation therapy to overcome depression and facilitate socio-economic transitions of adolescents in South Africa: pilot Randomised Control Trial
**Short Title**	DoBAt Study
**Countries of recruitment**	South Africa
**Health condition(s) or problem(s) studied**	Depression
**Intervention**	All participants in the intervention and control arms will be given a Samsung Galaxy A2 Core Android smartphone and receive active symptom monitoring via text messages sent to the smartphone every 2.5 weeks.<br>• The intervention arm will receive Behavioural Activation (BA) therapy via a smartphone application (the *Kuamsha* app) and supported by weekly phone calls from Trained Peer Mentors, implemented over 10 weeks.<br>• The control arm will receive a smartphone application (the *Kuchunguza* app) containing six module video clips from WildEarth-SafariLive, a locally produced wildlife series.
**Trial Participants**	Adolescents aged 15-19 with mild to moderately-severe depression based on scores between 5 and 19 on the 9-item Patient Health Questionnaire-Adolescent version (PHQ-A), that live in the MRC/Wits-Agincourt study area, Bushbuckridge, and from whom we obtain written informed assent and consent (including parental consent if <18 years).
**Study type**	Two-arm single-blind individually randomised controlled pilot trial
**Date of first enrolment**	25th November 2021
**Trial Status**	Enrolment of the first participant occurred on the 25th November 2021. We expect to finish recruitment during August 2022 and data collection in January 2023.
**Target sample size**	200
**Recruitment status**	Recruiting
**Primary outcomes**	1) to determine the feasibility and acceptability of the intervention<br>2) to provide preliminary evidence on the initial efficacy (direction and magnitude) of any effects of the intervention on depressive symptoms
**Key secondary outcomes**	1) to pilot a range of mental health, social-affective cognition, risky behaviours, and socioeconomic measures

For peer review only - http://bmjopen.bmj.com/site/about/guidelines.xhtml

	2) to collect descriptive data on trial procedures such as recruitment, retention, data collection, randomisation and blinding to inform the development of a further larger trial.
**Data sharing statement**	Individual participant quantitative data that underlie the results reported in each publication arising from the trial will be made available after deidentification. Any video, audio or qualitative data will not be available. Data will be available beginning 9 months and ending 36 months following main article publication to researchers who provide a methodologically sound proposal that purposes to achieve aims in the approved proposal and /or for individual participant data meta-analysis. Data is documented and stored on the MRC/Wits-Agincourt Data Repository with a digital object identifier (doi) and can be accessed with permission and in line with with MRC/Wits-Agincourt policies and procedures. Data requestors will need to sign a data access agreement before any data can be shared. In addition, Study Protocol and Statistical Analysis Plan documents will be available.

Table 4. The Song Contest story

Learning module	Description of each module	BA Learning principles
**Episode 1 (“Pick a team”)**	Students find out about a school Song Contest. The winning prize is a voucher to shop for a new outfit at the mall. User chooses the main character’s name and picks two teammates to join the Song Contest.<br>Main character gets anxious about the idea of performing in front of everyone else. Christine (teacher) talks about the benefits of stepping outside one’s comfort zone and offers her support.<br>At the end of the module, the user will be asked to set a goal to work on over the 10 weeks of intervention.	Absorption<br>TRAP-TRAC<br>Sleep<br>Self-confidence<br>Relapse prevention
**Episode 2 (“Find a cool tune”)**	First practice session with group which didn’t go well because one teammate fell asleep. Main character is frustrated and decides to ask Christine for advice. After hearing that teammate’s grandmother ill, main character showed leadership and compassion in how they handled the situation.	
**Episode 3 (“The Lure”)**	The team worked well together and made progress on the song. At the end of the practice, Prince (desirable character) invites the main character to go to the tavern. Main character is asked to think about the consequences of their actions but decides to go anyway.	
**Episode 4 (“The Fallout”)**	The next day the main character is exhausted, hungover, and unable to concentrate. They arrive late to practice session without homework done. Teammates get annoyed. Main character decides to apologise instead of avoiding the problem and their teammates are forgiving. Main character learns about the importance of sleep and goes to sleep early.	
**Episode 5 (“The Return”)**	Main character sleeps and feels recharged. Team is happy with the practice. Song is completed. Main character learns that they have to give a presentation if they win and asks Christine for advice. Main character learns ways to deal with being nervous and how to feel more self-confident.	
**Episode 6 (“The Big Day”)**	Team feels nervous before the show. They perform and win the prize. Group reflects on journey.	

For peer review only - http://bmjopen.bmj.com/site/about/guidelines.xhtml

Table 5. The Football Match story

Learning module	Description of each module	BA Learning principles
**Episode 1 ("Pick a team")**	Main character is the striker for the local team. User chooses the main character's name and picks a name for the football team. User gets introduced to the football task. At the end of the module, the user will be asked to set a goal to work on over the 10 weeks of intervention.	Absorption<br>Rumination<br>TRAP-TRAC<br>Problem-solving<br>Negotiation
**Episode 2 ("Game Over")**	Main character misses some important shots. Shane (antagonistic character) is a discouraging main character because of their poor performance. Team loses the game. Main character leaves the match alone feeling upset and discouraged, ruminating over her performance during the match. Bird offers different, more positive perspective on performance but the main character not in a frame of mind to believe it.	
**Episode 3 ("Hide away")**	Main character is frustrated, misses school and gets a detention. Main character feels a bit better after friends visit her/him at her house, but apprehensive about going to school. Main character plays the football task which makes them feel energised enough to do their homework.	
**Episode 4 ("That's okay")**	Main character returns to school feeling more positive after a good night's sleep. Main character handles Shane well, but their mood takes a dip. Main character decides to speak with Coach Bayer after school about their performance in the match. Main character learns that mistakes can be useful opportunities to learn, instead of something to avoid. Main character practises what they've learnt and starts to feel more confident.	
**Episode 5 ("Keep practising")**	Main character speaks with friends before practice about what they learnt from Coach Bayer and asks for help in dealing with Shane. With their supportive presence, main character gets Shane to agree to stay away from them so that they can focus on football and winning. Main character feels confident, keeps practising, and feels ready for the re-match.	
**Episode 6 ("The Big Match")**	It all came together for the main character, stepping out onto the pitch with new confidence. Main character keeps calm and meets their mistakes with curiosity instead of frustration. Main character scores the winning goal.	

For peer review only - http://bmjopen.bmj.com/site/about/guidelines.xhtml

Table 6. Example mock-ups of the Kuamsha app

Component	Description	Example
Home screen	This is the first screen that users will see as they open the Kuamsha app. Users will have the option to play through the stories, monitor their mood, play absorbing activities to improve focus, or report on their weekly activities (see below for further details on each of these components).	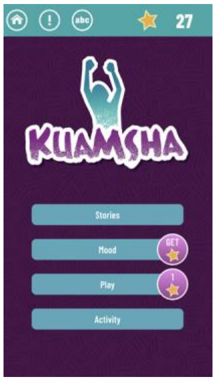
Log-in unlock code	Kuamsha is password-protected. Users will be asked to enter a password every time they access the app.	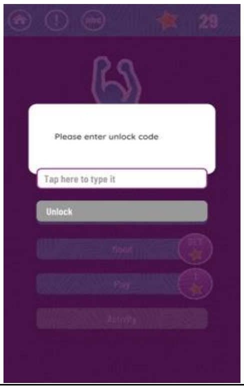
Language selector	Users will be able to select their preferred language. All the text in the app underwent two rounds of translation and has been checked by a clinical psychologist for accuracy.	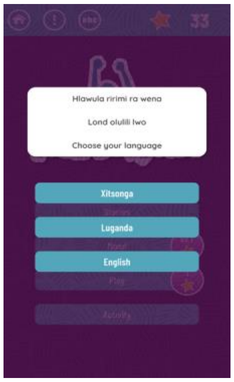
Story Selection	The core of the game consists of a choice between two narrative stories. Each consists of 6 modules that are played in sequential order. It is possible to begin one story and then switch to the other. During gameplay points are earned for the choices made and by completing other core game elements described below.	

For peer review only

<table>
<tr><td></td><td></td><td>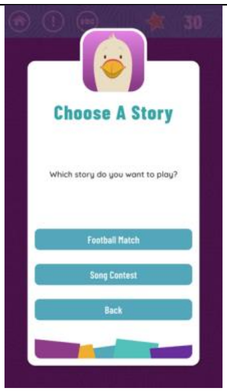
</td></tr>
<tr><td>Mood monitoring</td><td>Mood monitoring is an important part of BA and can be used as a tool for understanding how your mood is impacted and changes depending on the activities you are engaged in. Every time a user complete mood monitoring, they receive in-app points. Participants are asked to monitor their mood at various times (before and after each module, when they report a homework activity, and when they complete an absorbing activity).</td><td>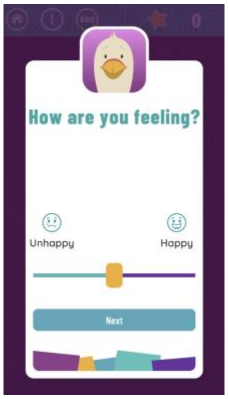
</td></tr>
</table>

For peer review only

SPIRIT
STANDARD PROTOCOL ITEMS: RECOMMENDATIONS FOR INTERVENTIONAL TRIALS

SPIRIT 2013 Checklist: Recommended items to address in a clinical trial protocol and related documents*

Section/item	Item No	Description	Page
**Administrative information**			
Title	1	Descriptive title identifying the study design, population, interventions, and, if applicable, trial acronym	1
Trial registration	2a	Trial identifier and registry name. If not yet registered, name of intended registry	1
	2b	All items from the World Health Organization Trial Registration Data Set	Sup. Material (Table 3)
Protocol version	3	Date and version identifier	Sup. Material (Table 3)
Funding	4	Sources and types of financial, material, and other support	12
Roles and responsibilities	5a	Names, affiliations, and roles of protocol contributors	Sup. Material (Table 1)
	5b	Name and contact information for the trial sponsor	Sup. Material (Table 3)
	5c	Role of study sponsor and funders, if any, in study design; collection, management, analysis, and interpretation of data; writing of the report; and the decision to submit the report for publication, including whether they will have ultimate authority over any of these activities	12
	5d	Composition, roles, and responsibilities of the coordinating centre, steering committee, endpoint adjudication committee, data management team, and other individuals or groups overseeing the trial, if applicable (see Item 21a for data monitoring committee)	Sup. Material (Table 2)

For peer review only

Introduction			
Background and rationale	6a	Description of research question and justification for undertaking the trial, including summary of relevant studies (published and unpublished) examining benefits and harms for each intervention	3-4
	6b	Explanation for choice of comparators	6
Objectives	7	Specific objectives or hypotheses	4
Trial design	8	Description of trial design including type of trial (eg, parallel group, crossover, factorial, single group), allocation ratio, and framework (eg, superiority, equivalence, noninferiority, exploratory)	5
**Methods: Participants, interventions, and outcomes**			
Study setting	9	Description of study settings (eg, community clinic, academic hospital) and list of countries where data will be collected. Reference to where list of study sites can be obtained	5
Eligibility criteria	10	Inclusion and exclusion criteria for participants. If applicable, eligibility criteria for study centres and individuals who will perform the interventions (eg, surgeons, psychotherapists)	5-6
Interventions	11a	Interventions for each group with sufficient detail to allow replication, including how and when they will be administered	6-7 (& Sup Material Tab 4-6)
	11b	Criteria for discontinuing or modifying allocated interventions for a given trial participant (eg, drug dose change in response to harms, participant request, or improving/worsening disease)	n/a
	11c	Strategies to improve adherence to intervention protocols, and any procedures for monitoring adherence (eg, drug tablet return, laboratory tests)	5-6
	11d	Relevant concomitant care and interventions that are permitted or prohibited during the trial	5
Outcomes	12	Primary, secondary, and other outcomes, including the specific measurement variable (eg, systolic blood pressure), analysis metric (eg, change from baseline, final value, time to event), method of aggregation (eg, median, proportion), and time point for each outcome. Explanation of the clinical relevance of chosen efficacy and harm outcomes is strongly recommended	7-9

For peer review only - http://bmjopen.bmj.com/site/about/guidelines.xhtml

Participant timeline	13	Time schedule of enrolment, interventions (including any run-ins and washouts), assessments, and visits for participants. A schematic diagram is highly recommended (see Figure)	Table 1 Figure 1
Sample size	14	Estimated number of participants needed to achieve study objectives and how it was determined, including clinical and statistical assumptions supporting any sample size calculations	11
Recruitment	15	Strategies for achieving adequate participant enrolment to reach target sample size	9
**Methods: Assignment of interventions (for controlled trials)**			
Allocation:			
Sequence generation	16a	Method of generating the allocation sequence (eg, computer-generated random numbers), and list of any factors for stratification. To reduce predictability of a random sequence, details of any planned restriction (eg, blocking) should be provided in a separate document that is unavailable to those who enrol participants or assign interventions	10
Allocation concealment mechanism	16b	Mechanism of implementing the allocation sequence (eg, central telephone; sequentially numbered, opaque, sealed envelopes), describing any steps to conceal the sequence until interventions are assigned	10
Implementation	16c	Who will generate the allocation sequence, who will enrol participants, and who will assign participants to interventions	10
Blinding (masking)	17a	Who will be blinded after assignment to interventions (eg, trial participants, care providers, outcome assessors, data analysts), and how	10
	17b	If blinded, circumstances under which unblinding is permissible, and procedure for revealing a participant's allocated intervention during the trial	10
**Methods: Data collection, management, and analysis**			
Data collection methods	18a	Plans for assessment and collection of outcome, baseline, and other trial data, including any related processes to promote data quality (eg, duplicate measurements, training of assessors) and a description of study instruments (eg, questionnaires, laboratory tests) along with their reliability and validity, if known. Reference to where data collection forms can be found, if not in the protocol	7-9

	18b	Plans to promote participant retention and complete follow-up, including list of any outcome data to be collected for participants who discontinue or deviate from intervention protocols	In SAP
Data management	19	Plans for data entry, coding, security, and storage, including any related processes to promote data quality (eg, double data entry; range checks for data values). Reference to where details of data management procedures can be found, if not in the protocol	In SAP
Statistical methods	20a	Statistical methods for analysing primary and secondary outcomes. Reference to where other details of the statistical analysis plan can be found, if not in the protocol	10-11
	20b	Methods for any additional analyses (eg, subgroup and adjusted analyses)	10-11
	20c	Definition of analysis population relating to protocol non-adherence (eg, as randomised analysis), and any statistical methods to handle missing data (eg, multiple imputation)	10-11
**Methods: Monitoring**			
Data monitoring	21a	Composition of data monitoring committee (DMC); summary of its role and reporting structure; statement of whether it is independent from the sponsor and competing interests; and reference to where further details about its charter can be found, if not in the protocol. Alternatively, an explanation of why a DMC is not needed	Sup. Material (Table 2)
	21b	Description of any interim analyses and stopping guidelines, including who will have access to these interim results and make the final decision to terminate the trial	In SAP
Harms	22	Plans for collecting, assessing, reporting, and managing solicited and spontaneously reported adverse events and other unintended effects of trial interventions or trial conduct	12
Auditing	23	Frequency and procedures for auditing trial conduct, if any, and whether the process will be independent from investigators and the sponsor	N/A
**Ethics and dissemination**			
Research ethics approval	24	Plans for seeking research ethics committee/institutional review board (REC/IRB) approval	11

Protocol amendments	25	Plans for communicating important protocol modifications (eg, changes to eligibility criteria, outcomes, analyses) to relevant parties (eg, investigators, REC/IRBs, trial participants, trial registries, journals, regulators)	Sup. Material (Table 3)
Consent or assent	26a	Who will obtain informed consent or assent from potential trial participants or authorised surrogates, and how (see Item 32)	11
	26b	Additional consent provisions for collection and use of participant data and biological specimens in ancillary studies, if applicable	N/A
Confidentiality	27	How personal information about potential and enrolled participants will be collected, shared, and maintained in order to protect confidentiality before, during, and after the trial	11-12
Declaration of interests	28	Financial and other competing interests for principal investigators for the overall trial and each study site	12
Access to data	29	Statement of who will have access to the final trial dataset, and disclosure of contractual agreements that limit such access for investigators	In SAP
Ancillary and post-trial care	30	Provisions, if any, for ancillary and post-trial care, and for compensation to those who suffer harm from trial participation	N/A
Dissemination policy	31a	Plans for investigators and sponsor to communicate trial results to participants, healthcare professionals, the public, and other relevant groups (eg, via publication, reporting in results databases, or other data sharing arrangements), including any publication restrictions	12
	31b	Authorship eligibility guidelines and any intended use of professional writers	12
	31c	Plans, if any, for granting public access to the full protocol, participant-level dataset, and statistical code	Sup. Material (Table 3)
Appendices			
Informed consent materials	32	Model consent form and other related documentation given to participants and authorised surrogates	Available on request
Biological specimens	33	Plans for collection, laboratory evaluation, and storage of biological specimens for genetic or molecular analysis in the current trial and for future use in ancillary studies, if applicable	N/A

For peer review only

*It is strongly recommended that this checklist be read in conjunction with the SPIRIT 2013 Explanation & Elaboration for important clarification on the items. Amendments to the protocol should be tracked and dated. The SPIRIT checklist is copyrighted by the SPIRIT Group under the Creative Commons "Attribution-NonCommercial-NoDerivs 3.0 Unported" license.

For peer review only

For peer review only - http://bmjopen.bmj.com/site/about/guidelines.xhtml

BMJ Open

Digital delivery of Behavioural Activation therapy to overcome depression and facilitate social and economic transitions of adolescents in South Africa (the DoBAt study): protocol for a pilot randomised controlled trial

Journal:	*BMJ Open*
Manuscript ID	bmjopen-2022-065977.R1
Article Type:	Protocol
Date Submitted by the Author:	15-Nov-2022

Complete List of Authors:	Moffett, Bianca; University of the Witwatersrand, MRC/Wits Rural Public Health and Health Transitions Research Unit (Agincourt), School of Public Health, Faculty of Health Sciences<br>Pozuelo, Julia; University of Oxford, Psychiatry; University of the Witwatersrand, MRC/Wits Rural Public Health and Health Transitions Research Unit (Agincourt), School of Public Health, Faculty of Health Sciences<br>van Heerden , Alastair ; Human Sciences Research Council, Center for Community Based Research<br>O'Mahen, Heather; University of Exeter, Mood Disorders Centre, Department of Psychology<br>Craske, Michelle; University of California Los Angeles, Psychology, Psychiatry and Biobehavioral Sciences<br>Sodi, Tholene; University of Limpopo, Psychology<br>Lund, Crick; University of Cape Town, Alan J Flisher Centre for Public Mental Health, Department of Psychiatry and Mental Health; King's College London, Centre for Global Mental Health, Institute of Psychiatry, Psychology and Neuroscience<br>Orkin, Kate; University of Oxford, Blavatnik School of Government<br>Kilford, Emma J; University College London, Department of Clinical, Educational and Health Psychology<br>Blakemore, Sarah-Jayne; University of Cambridge, Department of Psychology; University of the Witwatersrand, MRC/Wits Rural Public Health and Health Transitions Research Unit (Agincourt), School of Public Health, Faculty of Health Sciences<br>Mahmud, Mahreen; University of Exeter, Department of Economics<br>Musenge, Eustasius; University of the Witwatersrand, MRC/Wits Rural Public Health and Health Transitions Research Unit (Agincourt), School of Public Health, Faculty of Health Sciences; University of the Witwatersrand, Division of Epidemiology and Biostatistics, School of Public Health, Faculty of Health Sciences<br>Davis, Meghan; University of Oxford, Department of Psychiatry<br>Makhanya, Zamakhanya; University of the Witwatersrand, MRC/Wits Rural Public Health and Health Transitions Research Unit (Agincourt), School of Public Health, Faculty of Health Sciences<br>Baloyi, Tlangelani; University of Limpopo, Department of Psychology<br>Mahlangu, Daniel; University of the Witwatersrand, MRC/Wits Rural Public Health and Health Transitions Research Unit (Agincourt), School of

	Public Health, Faculty of Health Sciences<br>Chierchia, Gabriele; University of Cambridge, Department of Psychology<br>Fielmann, Sophie L.; University of Cambridge, Department of Psychology<br>Gómez-Olivé, F. Xavier; University of the Witwatersrand, MRC/Wits Rural Public Health and Health Transitions Research Unit (Agincourt), School of Public Health, Faculty of Health Sciences<br>Valodia, Imraan; University of the Witwatersrand, Southern Centre for Inequality Studies<br>Tollman, Stephen; University of the Witwatersrand, MRC/Wits Rural Public Health and Health Transitions Research Unit (Agincourt), School of Public Health, Faculty of Health Sciences; Umeå Universitet, Umeå Centre for Global Health Research, Division of Epidemiology and Global Health, Department of Public Health and Clinical Medicine<br>Kahn, Kathleen; University of the Witwatersrand, MRC/Wits Rural Public Health and Health Transitions Research Unit (Agincourt), School of Public Health, Faculty of Health Sciences; Umeå Universitet, Umeå Centre for Global Health Research, Division of Epidemiology and Global Health, Department of Public Health and Clinical Medicine<br>Stein, Alan; University of Oxford, Department of Psychiatry; University of the Witwatersrand, MRC/Wits Rural Public Health and Health Transitions Research Unit (Agincourt), School of Public Health, Faculty of Health Sciences

**Primary Subject Heading**:	Mental health
Secondary Subject Heading:	Mental health, Global health, Public health
Keywords:	Depression & mood disorders < PSYCHIATRY, Child & adolescent psychiatry < PSYCHIATRY, PUBLIC HEALTH

SCHOLARONE™
Manuscripts

Digital delivery of Behavioural Activation therapy to overcome depression and facilitate social and economic transitions of adolescents in South Africa (the DoBAt study): protocol for a pilot randomised controlled trial

Authors
Bianca D. Moffett*[1], Julia R. Pozuelo*[1,2], Alastair van Heerden[3], Heather A. O'Mahen[4], Michelle Craske[5], Tholene Sodi[6], Crick Lund[7,8], Kate Orkin[9], Emma J Kilford[10], Sarah-Jayne Blakemore[1,11], Mahreen Mahmud[12], Eustasius Musenge[1,16], Meghan Davis[2], Zamakhanya Makhanya[1], Tlangelani Baloyi[6], Daniel Mahlangu[1] Gabriele Chierchia[11], Sophie L. Fielmann[11], Xavier Gomez-Olive[1], Imraan Valodia[13], Stephen Tollman[1,14], Kathleen Kahn**[1,14], Alan Stein**[1,2,15]

* denotes joint first author
** denotes joint senior author

Affiliations
[1] MRC/Wits Rural Public Health and Health Transitions Research Unit (Agincourt), School of Public Health, Faculty of Health Sciences, University of the Witwatersrand, Johannesburg, South Africa
[2] Department of Psychiatry, University of Oxford, Oxford, United Kingdom
[3] Center for Community Based Research, Human Sciences Research Council, Pietermaritzburg, South Africa
[4] Mood Disorders Centre, Department of Psychology, University of Exeter, Exeter, United Kingdom
[5] Department of Psychology, Psychiatry and Biobehavioral Sciences at the University of California, Los Angeles, United States of America
[6]Department of Psychology, University of Limpopo, Polokwane, South Africa
[7]Alan J Flisher Centre for Public Mental Health, Department of Psychiatry and Mental Health, University of Cape Town, Cape Town, South Africa
[8] Centre for Global Mental Health, Institute of Psychiatry, Psychology and Neuroscience, King's College London, London, United Kingdom
[9] Blavatnik School of Government, University of Oxford, Oxford, United Kingdom
[10] Department of Clinical, Educational and Health Psychology, University College London, London, United Kingdom
[11] Department of Psychology, University of Cambridge, Cambridge, United Kingdom
[12]Department of Economics, University of Exeter, Exeter, United Kingdom
[13]Southern Centre for Inequality Studies, University of the Witwatersrand, Johannesburg, South Africa
[14] Umeå Centre for Global Health Research, Division of Epidemiology and Global Health, Department of Public Health and Clinical Medicine, Umeå University, Umeå, Sweden
[15]Africa Health Research Institute, KwaZulu Natal, South Africa
[16]Division of Epidemiology and Biostatistics, School of Public Health, Faculty of Health Sciences, University of the Witwatersrand, Johannesburg, South Africa

Corresponding author: Bianca D Moffett, MRC/Wits Rural Public Health and Health Transitions Research Unit (Agincourt), School of Public Health, Faculty of Health Sciences, University of the Witwatersrand, Johannesburg, South Africa, bianca.moffett@wits.ac.za

Word Count
Abstract: 298
Text: 4665(Excl. References, Tables and Figures)
Tables & Figures: 2 Tables, 1 Figure
References: 67

Keywords: depression, adolescents, pilot trial, feasibility, digital, app, Behavioural Activation

For peer review only - http://bmjopen.bmj.com/site/about/guidelines.xhtml

ABSTRACT

Introduction: Scalable psychological treatments to address depression among adolescents are urgently needed. This is particularly relevant to low- and middle-income countries where 90% of the world's adolescents live. Whilst digital delivery of Behavioural Activation presents a promising solution, its feasibility, acceptability, and effectiveness among adolescents in an African context remains to be shown.

Methods and analysis: This study is a two-arm single-blind individual-level randomised controlled pilot trial to assess the feasibility, acceptability, and initial efficacy of digitally delivered Behavioural Activation (BA) therapy amongst adolescents with depression. The intervention has been co-produced with adolescents at the study site. The study is based in the rural northeast of South Africa in the Bushbuckridge sub-district of Mpumalanga Province. A total of 200 adolescents with symptoms of mild to moderately-severe depression on the Patient Health Questionnaire Adolescent Version (PHQ-A) will be recruited (1:1 allocation ratio). The treatment group will receive Behavioural Activation therapy via a smartphone application (the Kuamsha app) supported by trained peer mentors. The control group will receive an Enhanced Standard of Care. The feasibility and acceptability of the intervention will be evaluated using a mixed-methods design, and signals of the initial efficacy of the intervention in reducing symptoms of depression will be determined on an intention-to-treat basis. Secondary objectives are to pilot a range of cognitive, mental health, risky behaviour, and socioeconomic measures; and to collect descriptive data on the feasibility of trial procedures to inform the development of a further larger trial.

Ethics and dissemination: This study has been approved by the University of the Witwatersrand Human Research Ethics Committee (MED20-05-011) and the Oxford Tropical Research Ethics Committee (OxTREC 34-20). Study findings will be published in scientific open access peer-reviewed journals, presented at scientific conferences, and communicated to participants, their caregivers, public sector officials, and other relevant stakeholders.

Trial registration: This trial was registered on 19 November 2020 with the South African National Clinical Trials Registry (DOH-27-112020-5741) and the Pan African Clinical Trials Registry (PACTR202206574814636).

STRENGTHS AND LIMITATIONS

- The intervention has been iteratively co-produced with local adolescents using multiple user-centred design methods to ensure that it was engaging, culturally relevant, and usable for the targeted population.
- We will use a rigorous mixed-methods design to assess the feasibility, acceptability, and initial efficacy of the digital intervention.
- Culturally adapted measures of social-affective cognition, as well as a range of relevant mental health, risky behaviours, and socioeconomic measures, will be piloted.
- We have excluded participants with severe depression and high risk suicidal ideation.
- This study uses the PHQ-A, a validated screening tool to screen for depression, but does not provide a clinical diagnosis of depression.

For peer review only - http://bmjopen.bmj.com/site/about/guidelines.xhtml

1. INTRODUCTION

Despite growing acknowledgement of the importance of adolescent mental health and the potential of investing in this formative period, it remains severely neglected[1]. This is particularly true in low and middle-income countries (LMICs), where 90% of the world's adolescents live, socioeconomic adversities affecting mental health are prevalent, and mental health resources remain poor[2]. Given that most mental disorders have their onset before 25 years of age, early identification and treatment of emergent mental disorders has the potential to reduce chronicity and sequelae for individuals, and be an efficient strategy for addressing population-level mental health[3]. Investments in mental health have the potential to support socioeconomic transitions and contribute to breaking the cycle of poverty and mental ill-health in LMICs[4].

Adolescence is a critical developmental period during which individuals develop their self-identity, acquire skills and preferences with which they navigate future challenges, and make decisions that can affect their long-term health, education, relationships, and employment prospects[5,6]. Higher-order cognitive functions such as executive function and social cognition, which regulate the ability to strategise, set and maintain goals, and build successful relationships with others, develop significantly during this period[7]. Thus, the physical, cognitive, social, and emotional capabilities acquired during adolescence lay a foundation for the wellbeing of individuals throughout their adult lives[8]. Depression interferes with the acquisition of these capacities, thus limiting young people's ability to fulfil their potential.

Globally, depression is one of the leading causes of disability amongst adolescents, and suicide is the third leading cause of death amongst the 15-19 year age group[2,9]. Left untreated, depression affects interpersonal relationships, interferes with schooling, and disrupts productivity[10]. It also has a marked negative effect on executive function and social cognition, with depressed individuals consistently underperforming on cognitive assessments compared to healthy controls[11,12]. Furthermore, depression in adolescence has been associated with a greater risk for substance use, poor sexual health, delinquency, and a significant reduction in future income[13,14]. Scalable psychological therapies to address depression amongst adolescents, particularly in low-resource contexts, are urgently needed.

Several barriers hinder progress in providing treatment to adolescents with depression in LMICs. Firstly, whilst various treatments for depression have been shown to be effective amongst adolescents in high-income contexts, few have been adapted for or rigorously evaluated in low-resource or culturally diverse settings[15,16]. Secondly, there is a major shortage of mental health professionals in most LMICs[17], and although task-sharing approaches present a promising strategy, they require substantial inputs in training and supervision of non-specialist healthcare workers[18]. Thirdly, there are significant challenges associated with relying on an already overburdened healthcare sector, not least that depression is rarely detected in primary care in most LMICs[19]. Finally, even in contexts where adolescent mental health services are available, stigma, lack of mental health awareness, and issues with the acceptability of services prevent adolescents from seeking care[20].

For peer review only

Digitally delivered Behavioural Activation (BA) therapy offers the potential to overcome a number of these barriers. BA is a highly effective psychological therapy that is easier to deliver and less costly than Cognitive Behavioural Therapy (CBT)[21,22]. Importantly, there is also evidence that it can be effectively adapted for use: 1) with adolescents[23,24], 2) in low-resource contexts and diverse cultural settings[25–27] and 3) in a digital format[28]. Delivering BA in a digital format may be particularly helpful in contexts where there is a paucity of mental health specialists and health systems are already juggling multiple competing priorities. It also offers the potential to overcome internalised barriers to care, such as stigma and lack of mental health awareness, and this form of guided self-help may be more acceptable to adolescents[20].

Despite the many potential advantages of digital mental health interventions, studies that have evaluated their effectiveness amongst adolescents have yielded mixed results [29,30]. Most of the evidence on the effectiveness of digitally-delivered BA comes from studies conducted in high-income countries[28], and it is, therefore, an open question whether it is possible to generalise these findings to an African context, where conditions and resources differ vastly [31,32]. Furthermore, studies of online self-help interventions amongst adults in LMICs showed high attrition rates[31], and adherence to mental health apps in all contexts is often low[33]. While some commercial smartphone apps attract more users, many have not been rigorously evaluated and show little fidelity to evidence-based treatments [34,35].

This study will address an important gap in the literature by providing evidence on the feasibility, acceptability, and initial efficacy of using digitally delivered Behavioural Activation to address depression amongst adolescents in an LMIC context. Furthermore, we will pilot a range of relevant mental health, risky behaviour and socioeconomic measures, as well as novel culturally adapted measures of executive function and social cognition.

Study objectives

The co-primary objectives of this pilot study are: 1) to determine the feasibility and acceptability of a digitally delivered Behavioural Activation therapy intervention for adolescents living with depression in the Bushbuckridge sub-district of Mpumalanga province, South Africa; and 2) to provide preliminary evidence on any signals of initial efficacy (direction and magnitude) of any effects of the intervention on depressive symptoms amongst adolescents in the intervention arm compared to control.

The secondary objectives are: 1) to pilot a range of mental health, social-affective cognition, risky behaviours, and socioeconomic measures; and 2) to collect descriptive data on trial procedures such as recruitment, retention, data collection, randomisation and blinding to inform key parameters in the development of a further large-scale trial.

2. METHODS AND ANALYSIS

This protocol is reported in accordance with the Standard Protocol Items: Recommendations for Intervention Trials (SPIRIT) 2013 statement[36]. The statistical aspects of the pilot study are summarised here with details fully described in a Statistical Analysis Plan (SAP) that will be available before the recruitment of the last participant.

For peer review only - http://bmjopen.bmj.com/site/about/guidelines.xhtml

2.1 Study design

The DoBAt study is a two-arm single-blind individual-level randomised controlled pilot feasibility trial. A total of 200 adolescents will be recruited (1:1 allocation ratio).

2.2 Study Setting

The study will be based in the Bushbuckridge sub-district of Mpumalanga Province, South Africa. Whilst South Africa is classified as a middle-income country, large inequalities exist, and the study setting represents a rural area characterised by socioeconomic disadvantage and high rates of youth unemployment. Since 1992 the MRC/Wits Rural Public Health and Health Transitions Research Unit (MRC/Wits-Agincourt) has collected population data, with vital events (pregnancy outcome, deaths, in- and out-migration) and household composition updated annually through its Health and socio-Demographic Surveillance System (HDSS). The total population under surveillance is currently ~116,000 inhabitants residing in 31 contiguous villages[37]. Recruitment will take place at schools within the study site. Notably, a previous study amongst adolescent females in this setting reported a prevalence of depressive symptoms of 18.2%[38] and yet access to treatment is severely limited. Similar data for males is not available.

2.3 Patient and Public Involvement

Adolescents and members of the public have been involved at several stages of the study. The intervention was developed through extensive formative research and user-centred design with adolescents in the study area. We also conducted participatory workshops with adolescents and met with local educators, healthcare workers, and relevant non-governmental organisations to obtain their input on trial methods, including recruitment and risk management strategies. We will seek the involvement of adolescents and members of the public in developing appropriate methods to disseminate study findings.

2.4 Eligibility criteria

To be eligible for inclusion in the pilot trial participants must: 1) be between 15 and 19 years of age and in grades 9 to 11 at the beginning of the study; 2) have symptoms of mild to moderately-severe depression indicated by a score between 5 and 19 on the Patient Health Questionnaire Adolescent Version (PHQ-A); 3) be able to read sufficiently in the local language (Xitsonga) to use the Kuamsha app; 4) intend to continue living in the study site for 12-weeks after the baseline assessment; and 5) provide written informed assent/consent to participate in the study, as well as parent/guardian consent if younger than 18 years.

Participants will be excluded if they: 1) have symptoms of severe depression as indicated by a score of >19 on the PHQ-A; 2) have current suicidal ideation with specific plans and means identified; 3) are receiving psychological treatment for a mental health condition at the time of enrolment; 4) have been hospitalised for at least five days for a severe psychiatric illness (specifically Bipolar Mood Disorder, Schizophrenia or other Psychotic Disorders) or life-

For peer review only - http://bmjopen.bmj.com/site/about/guidelines.xhtml

threatening or other serious medical illness; 5) have a history of Bipolar Mood Disorder, Schizophrenia or other Psychotic Disorders; 6) lack capacity to consent to treatment or research participation or to use the app. Participants excluded for points 1) and 2) will be assessed by the Risk Management Team and referred to local clinical services as per prior arrangements with local service providers.

2.5 Intervention

All participants in the intervention and control arms will be given the entry-level Samsung Galaxy A2 Core Android smartphone, which they can keep at the end of the study. Furthermore, participants in both groups will receive 200MB of mobile internet data at six different time points (0; 2.5; 5; 7.5; 11 and 24 weeks) to ensure they have data to use the app and complete the online surveys. Participants in both arms will receive active symptom monitoring via text messages sent to the smartphone every 2.5 weeks. Any adolescents who develop severe depression or high-risk suicidal ideation will be assessed by the Risk Management Team (further details provided in Table 1 of the Supplemental Materials) and referred to local clinical services. Adolescents started on anti-depressant medication or who receive psychological therapy because of these referrals will not be discontinued, but we will take note of concomitant care and will examine this using sensitivity analyses.

Control arm: Enhanced Standard of Care

The non-intervention arm will receive a control app (the *Kuchunguza app*) containing six video clips from *WildEarth-SafariLive*, a locally produced wildlife series. Each video clip takes approximately 15-20 minutes to complete and allows users to explore the African wilderness while listening to calming and atmospheric sounds. In the given context, the control represents an enhancement of standard care since most adolescents with depression would not usually receive any intervention or active symptom monitoring and referral.

Intervention arm: Kuamsha programme

The intervention arm will receive six modules of Behavioural Activation (BA) therapy via a smartphone application (the *Kuamsha app*) supported by trained Peer Mentors, implemented over 10 weeks. The app and phone calls from the Peer Mentors together comprise the Kuamsha programme.

The Kuamsha app is primarily an interactive narrative game consisting of six tailored modules (sessions) containing Behavioural Activation's core principles integrated into the gamified story content format[39]. Each module takes approximately 15-20 mins to complete, and they cover topics such as identifying and engaging in meaningful activities and using strategies to overcome barriers, for example, using problem-solving, effective communication, getting enough sleep, and disengaging from rumination. Each module is followed by a homework activity where the participant is encouraged to reflect on the BA principles outlined in each module and think about ways to apply the principles to their own lives. Participants will be asked to report often they completed the homework activities and their mood as they were doing these activities. The app includes game design elements to stimulate motivation and performance, including character personification, in-app points, and reminders/notifications. Example mock-ups of the Kuamsha app and a summary of each module are shown in Tables 2-4 in the Supplemental Materials.

For peer review only - http://bmjopen.bmj.com/site/about/guidelines.xhtml

The Kuamsha app will be supplemented by weekly phone calls (15 mins per module) from Trained Peer Mentors. There will be seven calls in total, including one introductory phone call and six calls to cover module content. Peer Mentors will attempt to reach participants by calling up to 5 times per week. The role of the Peer Mentors is mainly to support adherence and compliance with the app, troubleshoot problems related to the use of the app, and assist with the implementation of the homework activities. They will be trained not to provide additional advice or counselling and conduct their calls according to a pre-determined checklist of activities to help ensure fidelity. Peer Mentors will be Xitsonga-speaking students or recent graduates from the department of psychology or social work at an accredited South African university. The Trial Psychologist will train and supervise them according to the training manual developed specifically for the intervention.

2.6 Primary objectives and outcome measures

(1) Feasibility and acceptability of the intervention. A mixed-methods approach will be adopted to establish feasibility and acceptability.

- **Feasibility** will be assessed by collecting data on the following:
  (1) Recruitment (enrolment rate of eligible participants) and retention in the trial at the end of the intervention period (11 weeks).
  (2) Feasibility of testing procedures and data collection methods, including assessment of completion rates.
  (3) Treatment adherence rates, where adherence is defined as having opened at least 4 out of 6 of the app modules and as having completed 3 out of 6 phone calls with the peer mentor (excluding the introductory call). We will complement the treatment adherence rates with engagement metrics collected via the app (number of times participants logged into the app, number of modules opened and completed, total time spent on the app, number of weekly activities set to do, and number of times the participant completed the weekly activities).
- **Acceptability** of the intervention and study procedures will be assessed via:
  (1) An acceptability questionnaire conducted at the end of intervention assessment (week 11) with all participants. The questionnaire consists of three measures: Acceptability of Intervention Measure (AIM), Intervention Appropriateness Measure (IAM), and Feasibility of Intervention Measure (FIM)[40]. Each measure consists of 4 items. The total score ranges from 1 to 5 and is calculated by averaging response scores to the response categories. We will calculate an average score for each of the measures. We will inquire about the acceptability of the app using these three measures (to both the intervention and control groups) and the acceptability of the peer mentor programme (to the intervention group only).
  (2) In-depth interviews with a subsample of participants. We estimate that interviews with 20 participants will provide a sufficient range of experiences and perspectives to reach data saturation. This subsample will contain participants from the treatment and control arms and will be stratified based on high versus low app engagement.
- ***Fidelity of delivery of the intervention*** will be assessed by collecting data on adherence and competence of Trained Peer Mentors.

For peer review only - http://bmjopen.bmj.com/site/about/guidelines.xhtml

(1) Adherence of the peer mentors is defined as the number of sessions that meet at least 90% of the criteria for adherence according to the training protocol. Independent raters will listen to a random sample (10%) of recordings of Peer Mentors' phone calls with participants and assess them against the training protocol.
(2) Competence of the peer mentors will be expressed as a percentage based on their Competency Assessment Test. This test will include a written test and observation of skills through role-playing to assess Knowledge, Attitudes and Practices. Tests will be scored by the Trial Psychologist using a pre-determined scoring system.

Trial progression criteria

Feasibility of the intervention and trial procedures and progression criteria for a definitive RCT are given in Table 1. These will be based on a traffic light system of green (continue to RCT), amber (make modifications to trial procedures before embarking on a definitive RCT), and red (a definitive RCT is unlikely to be feasible)[41].

Table 1. Trial progression criteria

Criteria	Green	Amber	Red
Enrolment (recruitment) rate of eligible participants	≥60%	<60%, ≥40%	<40%
Retention in the trial at 11 weeks	≥90%	<90%, ≥50%	<50%
Proportion of participants that open at least 4 out of 6 of the app modules	≥70%	<70%, ≥50%	<50%
Proportion of participants that have 3 out of 6 phone calls with the peer mentor	≥70%	<70%, ≥50%	<50%

(2) Signals of Initial Efficacy on depressive symptoms.
This will be assessed using the PHQ-A score at the end of intervention assessment (week 11). The PHQ-A is a widely used and well-established measure of adolescent depressive symptoms over the past two weeks[42]. The PHQ-A will be administered in Xitsonga. Whilst this measure has not been validated in Xitsonga, it showed good psychometric properties with a sample of adolescents and young adults in South Africa and Kenya [43–45] and has been validated in various other South African languages which also form part of the 'Nguni' language group, including isiXhosa [46], seTswana [43], isiZulu and seSotho[44]. The questionnaire consists of 9 items. The PHQ-A total score ranges from 0 to 27 and is calculated by assigning scores to the response categories [0=not at all, 1=several days, 2=more than half the days, 3-nearly every day] and summing the score for each of the items. Higher scores indicate greater severity of depression. PHQ-A scores of 5, 10, 15, and 20 represent mild, moderate, moderately severe and severe depression, respectively [42]. We will use this scale to assess depressive symptoms at screening, week 5, week 11, and week 24. Participants will also be asked to complete this scale

For peer review only - http://bmjopen.bmj.com/site/about/guidelines.xhtml

at week 2.5 and week 7.5 as part of the symptom monitoring. More information on how we will analyse this data and trial progression criteria can be found in the data analysis section.

2.7 Secondary objectives and outcome measures

As part of our secondary objectives, we will pilot locally adapted measures of mental health, social-affective and cognitive processing, risky behaviours, and socioeconomic outcomes. The overall aim of implementing these measures is to assess their acceptability, feasibility, and variation, and use these descriptive data to inform the development of a future larger trial. More details on how we will analyse these secondary outcomes can be found in the Statistical Analysis Plan in the Supplemental Materials.

(i) *Mental health outcomes:* include the following scales Generalised Anxiety Disorder (GAD-7)[47]; Connor-Davidson Resilience Scale (CD-RISC) [48]; Brief Rumination Response Scale (RRS) [49]; Warwick-Edinburgh Mental Wellbeing Scale (WEMWBS)[50]; Behavioural Activation for Depression Scale (BADS)[51]. Participants will be asked to complete these scales at baseline (week 0), mid-intervention (week 5), end of intervention (week 11), and follow-up (week 24). See Table 2 for details.

(ii) Social-affective and cognitive processing: include five computerised cognitive tasks measuring affective set-shifting, affective working memory, risk-seeking preferences, emotion recognition, and abstract reasoning. The tasks include a modified version of the Wisconsin Card Sorting Task[52]; the Backward Digit Span Task[53]; the Balloon Analogue Risk Task (BART)[54]; an Emotion Recognition Task[55]; and the Matrix Reasoning Item Bank (MaRs-IB) Task[56]. Participants will be asked to complete these tasks at baseline (week 0) and end of intervention (week 11).

(iii) Risky behaviours: The questions on sexual behaviours include a subset of items from the Wits Reproductive Health and HIV Institute (Wits RHI) sexual behaviour survey[57]. We will measure substance use using the Alcohol, Smoking and Substance Involvement Screening Test for Youth (ASSIST-Y)[58]. We will also ask about delinquency, gambling, and peer influence in participants' engagement with risky behaviours. Participants will be asked to complete these tasks at baseline (week 0) and end of intervention (week 11).

(iv) Socioeconomics: We will measure economic preferences (time preference, risk preference, and loss aversion) using three incentivised tasks[59,60]. In addition, we will measure a range of socioeconomic outcomes, including measures of time use, human capital investment, and spending. Participants will be asked to complete these tasks at baseline (week 0) and end of intervention (week 11).

2.8 Recruitment and participant timeline

Participants will be recruited through a two-stage recruitment process. The first stage of recruitment (Phase 1) will consist of a screening survey conducted in schools to identify adolescents with symptoms of mild to moderately-severe depression. The second stage of recruitment (Phase 2) will be done with adolescents who score between 5 and 19 on the PHQ-A in the school survey and who also meet the other eligibility criteria.

For peer review only - http://bmjopen.bmj.com/site/about/guidelines.xhtml

The pilot trial consists of an 11-week intervention treatment phase, and participants will be followed up for a further 13 weeks after completing the intervention (i.e., a total of 24 weeks). Primary and secondary outcomes will be assessed at the end of intervention assessment (week 11). We will use different methods to assess these outcomes. Specifically, we will ask about mental health (including PHQ-A) and risky behaviours using Audio Computer-Assisted Self-Interviewing (ACASI) software. Under this approach, participants will listen to pre-recorded survey questions through headphones and select their responses on a tablet computer. The behavioural tasks will be performed individually by participants on a tablet computer, in a quiet location under the instruction of a trained fieldworker. All app engagement metrics (including treatment adherence) will be captured on an online database automatically as participants engage with the app. The acceptability questionnaire will be sent via text message to the study phones. The socioeconomic outcomes will be assessed by blinded outcomes assessors. Table 2 indicates the time schedule of enrolment, interventions and assessments for participants, and Figure 1 shows the flow of participants.

Table 2. Schedule of enrolment, interventions, and assessments for participants

	STUDY PERIOD							
Assessment	Enrolment	Allocation	Post-allocation					Close-out
TIME POINT (in weeks)	$-t_1$	0	1	2.5	5	7.5	11	24
ENROLMENT								
Eligibility screen (school survey)	X							
Informed consent	X							
Allocation		X						
Phone delivery		X						
INTERVENTION								
Intervention arm: Kuamsha app & Peer Mentor calls			●	—	—	—	●	
Control arm: Kuchunguza app			●	—	—	—	●	
ASSESSMENTS								
School survey: PHQ-A, sociodemographic questions & eligibility checklist	X							
Baseline assessment: demographic questionnaire, GAD-7, CD-RISC, RRS, WEMWBS, BADS, five social-affective and cognition tasks, risky behaviours, and socioeconomic outcomes	X							
Symptom monitoring: PHQ-A				X		X		
Mid-intervention assessment: PHQ-A, GAD-7, RRS, WEMWBS short form, BADS (activation & social impairment subscales)					X			

For peer review only - http://bmjopen.bmj.com/site/about/guidelines.xhtml

End of intervention assessment: same as baseline + PHQ-A + acceptability questionnaire & in-depth interviews with subsample							X	
Follow-up assessment: PHQ-A + GAD-7, RRS, BADS (activation subscale), WEMWBS short form								X

Note: BADS: Behavioural Activation for Depression Scale; CD-RISC: Connor-Davidson Resilience Scale; GAD-7: Generalised Anxiety Disorder; PHQ-A: Patient Health Questionnaire Adolescent Version; RRS: Brief Rumination Response Scale; WEMWBS: Warwick-Edinburgh Mental Wellbeing Scale.

2.9 Allocation and blinding

Participants will be randomly assigned to the intervention or control arm with a 1:1 allocation using a computerised minimisation algorithm, balanced by sex (male or female) and severity of depressive symptoms (<10 or ≥ 10 on the PHQ-A). The minimisation algorithm was generated by the Centre for Healthcare Randomised Trials (CHaRT) at the University of Aberdeen. Participants will be allocated using CHaRT's online software and the Trial Manager will oversee the enrolment of participants as per assignment.

For practical and risk management reasons the Trial Manager, Trial Psychologist, Peer Mentors, and Fieldworkers delivering the phones to participants and receiving calls to troubleshoot problems with the app/ phone (i.e., "Phones Team") will not be blinded. However, all fieldworkers conducting outcomes assessments (i.e., the "Assessments Team") will be blinded to participants' allocation status. Any breaches in blinding will be documented, and we will ensure to change the fieldworker conducting subsequent assessments on a particular adolescent where unblinding has occurred.

2.10 Analysis and statistical methods

Quantitative data will be analysed using *Stata* Version 14.0[61], R[62] and other appropriate statistical analysis packages under the direction of the trial statistician.

Participants will be analysed in the groups to which they are randomly assigned, regardless of deviation from the protocol or treatment received (intention to treat population), and the data analyst will be blind to arm allocation. We will use descriptives to explore patterns in the data, followed by inferential statistics involving univariate and multivariable models. A two-tailed p-value <0.05 will be considered statistically significant in the inferential analyses.

Primary objectives

Our first co-primary objective (feasibility and acceptability) will be assessed using a mixed-methods approach. We will compute appropriate summary statistics (e.g., proportions, means, SDs, etc.) for each quantitative outcome and evaluate the feasibility of the trial based on the pre-defined progression criteria discussed earlier.

For peer review only - http://bmjopen.bmj.com/site/about/guidelines.xhtml

Qualitative interviews will be audio-recorded, transcribed verbatim and translated into English. Transcripts will be analysed using thematic analysis. We will follow Braun and Clarke's six phases of analysis (i.e. becoming familiar with the data, generating initial codes, searching for themes, reviewing themes, defining and naming themes, and producing the report)[63]. Coding will be done by two independent researchers. NVivo10, a computer program that aids in the sorting and management of qualitative data, will be used to facilitate the analysis.

Our second co-primary objective (initial efficacy on depressive symptoms) will be based on the PHQ-A score (continuous) at 11 weeks. Outcomes will be compared between intervention and control groups using a linear regression model and adjusting for covariates (PHQ score measured at baseline, sex, age, depression severity, and household asset index). As a secondary analysis, we will make use of the repeated measurements of the PHQ-A throughout the trial (up to 6 times per individual) to evaluate the treatment effects over time. The details of this analysis will be documented in the SAP.

Secondary objectives

The descriptive data of the secondary outcomes will include possible floor and ceiling effects, accuracy, duration, latency, and efficiency. We will perform standard psychometric tests to examine whether the instruments perform well in the study context (e.g., reliability, validity, acceptability) and calculate bivariate correlations across variables of interest. We anticipate that some secondary analyses will be included in companion papers rather than the main paper. The details of this analysis will be documented in a separate analysis plan.

2.11 Sample size considerations

The sample size was calculated on the basis of one of our co-primary objectives which aims to provide preliminary evidence of signals of efficacy (direction and magnitude) of any effects of the intervention on depressive symptoms (measured by the PHQ-A) amongst adolescents in the intervention arm compared to the control group.

Statistical power was calculated to detect differences between two independent groups, in a two-sided test with an α of 0.05 and a power of $1-\beta=0.80$, for an effect size of 0.45. This effect size is based on findings from previous studies investigating digital psychological interventions, with effect size (Cohen's d) ranging from 0.24 to 0.57[29,64,65]. Given these findings, we chose a small-to-medium effect size of 0.45 to account for the limited number of studies conducted with our target population and in our study setting. We allowed for 25% attrition, based on a previous study with adolescents in the Agincourt setting[66]. Given these assumptions, we aimed to recruit 200 participants at baseline. We calculated the required sample size using G*Power software, version 3.1.9.3[67].

2.12 Trial status

Enrolment of the first participant occurred on the 25th of November 2021. The trial is currently ongoing, and we expect to finish data collection in January 2023.

For peer review only - http://bmjopen.bmj.com/site/about/guidelines.xhtml

3. ETHICS AND DISSEMINATION

3.1 Research Ethics Approval

Ethical approval for this study has been obtained from the University of the Witwatersrand Human Research Ethics Committee (MED20-05-011), Ehlanzeni District and Mpumalanga Provincial Departments of Health and Education, and the Oxford Tropical Research Ethics Committee (OxTREC 34-20). This trial was registered with the South African National Clinical Trials Registry (DOH-27-112020-5741) and the Pan African Clinical Trials Registry (PACTR202206574814636) in November 2020. Further details on Trial Registration can be found in Table 5 of the Supplemental Material.

3.2 Informed Consent

At both stages of recruitment, we will obtain informed consent from participants aged 18 or over and informed assent and parental/guardian consent from participants younger than 18. All information sheets will be available in Xitsonga and English, and assent/consent will be obtained by trained and supervised bilingual fieldworkers.

3.3 Confidentiality and management of participant data

The confidentiality of participants' data and information will be respected and maintained by all study staff. Staff members will be trained accordingly and required to sign a non-disclosure agreement. A unique participant identification number will be used to link study data and information. All electronic data will be stored in a secure, protected and access-controlled database at the Agincourt data centre. Paper-based documentation will be stored in a locked cabinet and only accessible to authorised staff. Confidentiality may be broken in instances of immediate harm to self or others, as detailed in consent and assent forms.

3.4 Harms/ Anticipated risks

A comprehensive Risk Management Protocol has been developed to ensure the safety of participants in the trial. Participants with severe depression, suicidal ideation and other risks will receive a telephonic and/or in-person assessment by a member of the Risk Management Team. Subsequently, appropriate referrals and linkage to care will be made using established networks with local providers. Details of the independent Data and Safety Monitoring Board (DSMB) and Trial Steering Committee (TSC) are provided in Table 1 of the Supplemental Materials. Serious Adverse Events will be reported to the DSMB chair within 48 hours and to both Ethics Committees within seven days. Summary tables of all Adverse Events will be sent to the DSMB on a quarterly basis and to the Ethics Committees on an annual basis.

3.5 Publication and dissemination

Results of the trial will be communicated to participants, the public, researchers, healthcare professionals, and policymakers. We will seek the involvement of adolescents and members of the public in the development of appropriate methods to disseminate study findings. Policymakers and other key stakeholders within provincial and national departments of health, education, and social development, as well as relevant local NGOs, will be engaged in dialogue and supplied with a technical brief to convey the results of the trial and their implications for policy and practice. Trial results will be published as soon as possible after completion, and ensuing publications will be made open access. Results will also be presented at relevant national

For peer review only - http://bmjopen.bmj.com/site/about/guidelines.xhtml

and international conferences. Authorship will be determined in accordance with the ICMJE guidelines, and other contributors will be acknowledged.

Acknowledgements

We would like to acknowledge the Field Supervisors Meriam Meritze, Princess Makhubela and Nokthula Mayindi, as well as all the Fieldworkers, Registered Counsellor, Tinyiko Mafumo, and Peer Mentors for their hard work and dedication. We would also like to thank all the adolescents who were involved in the formative work and participatory workshops that were integral to the design and development of the intervention, as well as the educators, healthcare workers and non-governmental organisations that provided valuable input into the trial design, particularly regarding recruitment and risk management strategies.

Funding Statement

Research reported in this publication was supported by the South African Medical Research Council with funds received from the South African National Department of Health and the UK Medical Research Council, with funds received from the UK Government's Newton Fund (grant number MR/S008748/1). It also received financial support from the NIHR Oxford Health Biomedical Research Centre (grant number: N/A). The study was conducted within the MRC/Wits Rural Public Health and Health Transitions Research Unit and Agincourt Health and socio-Demographic Surveillance System, a node of the South African Population Research Infrastructure Network (SAPRIN), supported by the Department of Science and Innovation, the University of the Witwatersrand, and the Medical Research Council, South Africa (grant number: N/A). SJB is funded by Wellcome (grant number WT107496/Z/15/Z), the MRC-UK, the Jacobs Foundation, the Wellspring Foundation, and the University of Cambridge (grant number: N/A). JRP is funded by Fundación Rafael del Pino and received funding support from MRC UK and the Wellspring Philanthropic Fund (grant number: N/A). EJK received funding support from a UKRI-GCRF Impact Acceleration Award (grant number: N/A). Study funders played no role in the study design, collection, management, analysis or interpretation of data, the writing of this report, or the decision to submit it for publication.

Competing Interests

The authors declare no competing interests.

Authors' contributions

AS, KK, ST, MC, HO, SJB, CL, TS, KO, JRP, EJK, BM, MM, XGO, IV and AvH made substantial contributions to the conception and design of the study. BM, JRP, MD, SLF, GC and DM were responsible for programming study measures and training field teams. ZM and TB trained and supervised peer mentors. JRP, KO and EM drafted the Statistical Analysis Plan. The first draft of the manuscript was written by BM and JRP. All authors reviewed the manuscript, and gave the final approval of the version to be published. BM submitted the manuscript.

For peer review only - http://bmjopen.bmj.com/site/about/guidelines.xhtml

REFERENCES

1 Kieling C, Baker-Henningham H, Belfer M, *et al.* Child and adolescent mental health worldwide: evidence for action. *Lancet* 2011;**378**:1515–25. doi:10.1016/S0140-6736(11)60827-1

2 Erskine HE, Moffitt TE, Copeland WE, *et al.* A heavy burden on young minds: the global burden of mental and substance use disorders in children and youth. *Psychol Med* 2015;**45**:1551. doi:10.1017/S0033291714002888

3 Herrman H, Patel V, Kieling C, *et al.* Time for united action on depression: a Lancet–World Psychiatric Association Commission. *Lancet* 2022;**399**:957–1022. doi:10.1016/S0140-6736(21)02141-3

4 Lund C, De Silva M, Plagerson S, *et al.* Poverty and mental disorders: breaking the cycle in low-income and middle-income countries. *Lancet* 2011;**378**:1502–14. doi:10.1016/S0140-6736(11)60754-X

5 Sawyer SM, Afifi RA, Bearinger LH, *et al.* Adolescence: A foundation for future health. Lancet. 2012;**379**:1630–40. doi:10.1016/S0140-6736(12)60072-5

6 Almlund M, Duckworth AL, Heckman J, *et al.* Personality Psychology and Economics. In: Hanushek EA, Machin SJ, Woessman L, eds. *Handbook of the Economics of Education*. Elsevier 2011. 1–181. doi:10.1016/B978-0-444-53444-6.00001-8

7 Blakemore SJ, Choudhury S. Development of the adolescent brain: implications for executive function and social cognition. *J Child Psychol Psychiatry* 2006;**47**:296–312. doi:10.1111/J.1469-7610.2006.01611.X

8 Sheehan P, Sweeny K, Rasmussen B, *et al.* Building the foundations for sustainable development: a case for global investment in the capabilities of adolescents. *Lancet* 2017;**390**:1792–806. doi:10.1016/S0140-6736(17)30872-3

9 Kieling C, Adewuya A, Fisher HL, *et al.* Identifying depression early in adolescence. *Lancet Child Adolesc Heal* 2019;**3**:211. doi:10.1016/S2352-4642(19)30059-8

10 Thapar A, Collishaw S, Pine DS, *et al.* Depression in adolescence. Lancet. 2012;**379**:1056–67. doi:10.1016/S0140-6736(11)60871-4

11 Snyder HR. Major depressive disorder is associated with broad impairments on neuropsychological measures of executive function: A meta-analysis and review. *Psychol Bull* 2013;**139**:81–132. doi:10.1037/a0028727

12 Weightman MJ, Air TM, Baune BT. A review of the role of social cognition in major depressive disorder. Front. Psychiatry. 2014;**5**:179. doi:10.3389/fpsyt.2014.00179

13 Pozuelo JR, Desborough L, Stein A, *et al.* Systematic Review and Meta-Analysis: Depressive Symptoms and Risky Behaviours among Adolescents in Low- and Middle-Income Countries. *J Am Acad Child Adolesc Psychiatry* Published Online First: 2021. doi:10.1016/j.jaac.2021.05.005

14 Lund C, Myer L, Stein DJ, *et al.* Mental illness and lost income among adult South Africans. *Soc Psychiatry Psychiatr Epidemiol* 2013;**48**:845–51. doi:10.1007/s00127-012-0587-5

15 Das JK, Salam RA, Lassi ZS, *et al.* Interventions for Adolescent Mental Health: An Overview of Systematic Reviews. *J Adolesc Heal* 2016;**59**:S49. doi:10.1016/J.JADOHEALTH.2016.06.020

16 Klasen H, Crombag AC. What works where? A systematic review of child and adolescent mental health interventions for low and middle income countries. *Soc Psychiatry*

For peer review only - http://bmjopen.bmj.com/site/about/guidelines.xhtml

Psychiatr Epidemiol 2013;**48**:595–611. doi:10.1007/S00127-012-0566-X/TABLES/3
17 Kakuma R, Minas H, Van Ginneken N, *et al.* Human resources for mental health care: current situation and strategies for action. *Lancet* 2011;**378**:1654–63. doi:10.1016/S0140-6736(11)61093-3
18 Kemp CG, Petersen I, Bhana A, *et al.* Supervision of Task-Shared Mental Health Care in Low-Resource Settings: A Commentary on Programmatic Experience. *Glob Heal Sci Pract* 2019;**7**:150–9. doi:10.9745/GHSP-D-18-00337
19 Fekadu A, Demissie M, Birhane R, *et al.* Under detection of depression in primary care settings in low and middle-income countries: a systematic review and meta-analysis. *Syst Rev* 2022;**11**:1–10. doi:10.1186/S13643-022-01893-9/FIGURES/3
20 Stunden C, Zasada J, VanHeerwaarden N, *et al.* Help-Seeking Behaviors of Transition-Aged Youth for Mental Health Concerns: Qualitative Study. *J Med Internet Res* 2020;**22**:e18514. doi:10.2196/18514
21 Richards DA, Ekers D, McMillan D, *et al.* Cost and Outcome of Behavioural Activation versus Cognitive Behavioural Therapy for Depression (COBRA): a randomised, controlled, non-inferiority trial. *Lancet* 2016;**388**:871–80. doi:10.1016/S0140-6736(16)31140-0
22 Ekers D, Webster L, Van Straten A, *et al.* Behavioural activation for depression; An update of meta-analysis of effectiveness and sub group analysis. *PLoS One* 2014;**9**:e100100. doi:10.1371/journal.pone.0100100
23 Tindall L, Mikocka-Walus A, McMillan D, *et al.* Is behavioural activation effective in the treatment of depression in young people? A systematic review and meta-analysis. *Psychol Psychother Theory, Res Pract* 2017;**90**:770–96. doi:10.1111/PAPT.12121
24 Martin F, Oliver T. Behavioral activation for children and adolescents: a systematic review of progress and promise. *Eur Child Adolesc Psychiatry* 2019;**28**:427–41. doi:10.1007/S00787-018-1126-Z/FIGURES/2
25 Jacob M, L. Keeley M, Ritschel L, *et al.* Behavioural activation for the treatment of low-income, African American adolescents with major depressive disorder: a case series. *Clin Psychol Psychother* 2013;**20**:87–96. doi:10.1002/CPP.774
26 Patel V, Weobong B, Weiss HA, *et al.* The Healthy Activity Program (HAP), a lay counsellor-delivered brief psychological treatment for severe depression, in primary care in India: a randomised controlled trial. *Lancet* 2017;**389**:176–85. doi:10.1016/S0140-6736(16)31589-6
27 Lehmann DC, Bördlein C. A Systematic Review of Culturally Adapted Behavioral Activation Treatments for Depression: *Res Soc Work Pract* 2020;**30**:688–702. doi:10.1177/1049731520915635
28 Huguet A, Miller A, Kisely S, *et al.* A systematic review and meta-analysis on the efficacy of Internet-delivered behavioral activation. *J Affect Disord* 2018;**1**:27–38.
29 Lehtimaki S, Martic J, Wahl B, *et al.* Evidence on Digital Mental Health Interventions for Adolescents and Young People: Systematic Overview. *JMIR Ment Heal* 2021;**8**:e25847. doi:10.2196/25847
30 Eilert N, Wogan R, Leen A, *et al.* Internet-Delivered Interventions for Depression and Anxiety Symptoms in Children and Young People: Systematic Review and Meta-analysis. *JMIR Pediatr Parent 2022;5(2)e33551 https//pediatrics.jmir.org/2022/2/e33551* 2022;**5**:e33551. doi:10.2196/33551
31 Naslund JA, Aschbrenner KA, Araya R, *et al.* Digital technology for treating and

For peer review only - http://bmjopen.bmj.com/site/about/guidelines.xhtml

preventing mental disorders in low-income and middle-income countries: a narrative review of the literature. *The Lancet Psychiatry* 2017;**4**:486–500. doi:10.1016/S2215-0366(17)30096-2
32 Lehtimaki S, Martic J, Wahl B, *et al.* Evidence on Digital Mental Health Interventions for Adolescents and Young People: Systematic Overview. *JMIR Ment Heal* 2021;**8**. doi:10.2196/25847
33 Linardon J, Fuller-Tyszkiewicz M. Attrition and adherence in smartphone-delivered interventions for mental health problems: A systematic and meta-analytic review. *J Consult Clin Psychol* 2020;**88**:1–13. doi:10.1037/CCP0000459
34 Schueller SM, Torous J. Scaling evidence-based treatments through digital mental health. *Am Psychol* 2020;**75**:1093–104. doi:10.1037/AMP0000654
35 Huguet A, Rao S, McGrath PJ, *et al.* A Systematic Review of Cognitive Behavioral Therapy and Behavioral Activation Apps for Depression. *PLoS One* 2016;**11**:e0154248. doi:10.1371/JOURNAL.PONE.0154248
36 Chan AW, Tetzlaff JM, Altman DG, *et al.* SPIRIT 2013 statement: Defining standard protocol items for clinical trials. *Ann Intern Med* 2013;**158**:200–7. doi:10.7326/0003-4819-158-3-201302050-00583
37 Kahn K, Collinson MA, Gómez-Olivé FX, *et al.* Profile: Agincourt health and socio-demographic surveillance system. *Int J Epidemiol* 2012;**41**:988–1001. doi:10.1093/ije/dys115
38 Goin DE, Pearson RM, Craske MG, *et al.* Depression and incident HIV in adolescent girls and young women in HPTN 068: Targets for prevention and mediating factors. *Am J Epidemiol* Published Online First: 29 October 2019. doi:10.1093/aje/kwz238
39 Jacobson NS, Martell CR, Dimidjian S. Behavioral Activation Treatment for Depression: Returning to Contextual Roots. *Clin Psychol Sci Pract* 2006;**8**:255–70. doi:10.1093/clipsy.8.3.255
40 Weiner BJ, Lewis CC, Stanick C, *et al.* Psychometric assessment of three newly developed implementation outcome measures. *Implement Sci* 2017;**12**:1–12. doi:10.1186/S13012-017-0635-3/TABLES/3
41 Avery KNL, Williamson PR, Gamble C, *et al.* Informing efficient randomised controlled trials: exploration of challenges in developing progression criteria for internal pilot studies. *BMJ Open* 2017;**7**:e013537. doi:10.1136/BMJOPEN-2016-013537
42 Kroenke K, Spitzer RL, Williams JBW. The PHQ-9: Validity of a brief depression severity measure. *J Gen Intern Med* Published Online First: 2001. doi:10.1046/j.1525-1497.2001.016009606.x
43 Bhana A, Rathod SD, Selohilwe O, *et al.* The validity of the Patient Health Questionnaire for screening depression in chronic care patients in primary health care in South Africa. *BMC Psychiatry* 2015;**15**:118. doi:10.1186/s12888-015-0503-0
44 Cholera R, Gaynes BN, Pence BW, *et al.* Validity of the patient health questionnaire-9 to screen for depression in a high-HIV burden primary healthcare clinic in Johannesburg, South Africa. *J Affect Disord* 2014;**167**:160–6. doi:10.1016/j.jad.2014.06.003
45 Osborn TL, Venturo-Conerly KE, Wasil AR, *et al.* Depression and Anxiety Symptoms, Social Support, and Demographic Factors Among Kenyan High School Students. *J Child Fam Stud* 2019;:1–12. doi:10.1007/s10826-019-01646-8
46 Marlow M, Skeen S, Grieve CM, *et al.* Detecting Depression and Anxiety Among Adolescents in South Africa: Validity of the isiXhosa Patient Health Questionnaire-9 and

For peer review only - http://bmjopen.bmj.com/site/about/guidelines.xhtml

Generalized Anxiety Disorder-7. *J Adolesc Heal* 2022;**0**. doi:10.1016/J.JADOHEALTH.2022.09.013
47 Spitzer RL, Kroenke K, Williams JBW, *et al.* A brief measure for assessing generalized anxiety disorder: the GAD-7. *Arch Intern Med* 2006;**166**:1092–7. doi:10.1001/ARCHINTE.166.10.1092
48 Connor KM, Davidson JRT. Development of a new resilience scale: the Connor-Davidson Resilience Scale (CD-RISC). *Depress Anxiety* 2003;**18**:76–82. doi:10.1002/DA.10113
49 Treynor W, Gonzalez R, Nolen-Hoeksema S. Rumination reconsidered: A psychometric analysis. *Cognit Ther Res* 2003;**27**:247–59. doi:10.1023/A:1023910315561
50 Tennant R, Hiller L, Fishwick R, *et al.* The Warwick-Dinburgh mental well-being scale (WEMWBS): Development and UK validation. *Health Qual Life Outcomes* 2007;**5**:1–13. doi:10.1186/1477-7525-5-63/TABLES/4
51 Kanter JW, Mulick PS, Busch AM, *et al.* The Behavioral Activation for Depression Scale (BADS): Psychometric properties and factor structure. *J Psychopathol Behav Assess* 2007;**29**:191–202. doi:10.1007/S10862-006-9038-5
52 Schweizer S, Parker J, Leung JT, *et al.* Age-related differences in affective control and its association with mental health difficulties. *cambridge.org* Published Online First: 2019. doi:10.1017/S0954579419000099
53 Jahanshahi M, Saleem T, Ho AK, *et al.* A preliminary investigation of the running digit span as a test of working memory. *Behav Neurol* 2008;**20**:17–25. doi:10.3233/BEN-2008-0212
54 White TL, Lejuez CW, de Wit H. Test-Retest Characteristics of the Balloon Analogue Risk Task (BART). *Exp Clin Psychopharmacol* 2008;**16**:565–70. doi:10.1037/a0014083
55 Fuhrmann D, Knoll LJ, Sakhardande AL, *et al.* Perception and recognition of faces in adolescence. *Sci Rep* 2016;**6**:33497. doi:10.1038/srep33497
56 Chierchia G, Fuhrmann D, Knoll LJ, *et al.* The matrix reasoning item bank (MaRs-IB): novel, open-access abstract reasoning items for adolescents and adults. *royalsocietypublishing.org* 2019;**6**. doi:10.1098/rsos.190232
57 Pettifor A, Rees H, Steffenson A, *et al.* HIV and sexual behaviour among young South Africans: a national survey of 15-24 year olds. *Reprod Heal Res Unit, Univ Witswatersrand* 2004.
58 Humeniuk R, Holmwood C, Beshara M, *et al.* ASSIST-Y V1.0: First-Stage Development of the WHO Alcohol, Smoking and Substance Involvement Screening Test (ASSIST) and Linked Brief Intervention for Young People. *J Child Adolesc Subst Abuse* 2016;**25**:384–90. doi:10.1080/1067828X.2015.1049395
59 Andersen S, Harrison GW, Lau MI, *et al.* Elicitation using multiple price list formats. *Exp Econ* 2006;**9**:383–405. doi:10.1007/s10683-006-7055-6
60 Kahneman D, Tversky A. Prospect theory: An analysis of decision under risk. In: *Experiments in Environmental Economics*. Taylor and Francis Inc. 2018. 143–72. doi:10.2307/1914185
61 StataCorp. Stata Statistical Software: Release 14. 2015.
62 Team RC. R: A Language and Environment for Statistical Computing. 2021.
63 Braun V, Clarke V. Using thematic analysis in psychology. *Qual Res Psychol* 2006;**3**:77–101. doi:10.1191/1478088706qp063oa
64 Arjadi R, Nauta MH, Scholte WF, *et al.* Internet-based behavioural activation with lay counsellor support versus online minimal psychoeducation without support for treatment

For peer review only - http://bmjopen.bmj.com/site/about/guidelines.xhtml

of depression: a randomised controlled trial in Indonesia. *The Lancet Psychiatry* 2018;**5**:707–16. doi:10.1016/S2215-0366(18)30223-2
65 Fu Z, Burger H, Arjadi R, *et al.* Effectiveness of digital psychological interventions for mental health problems in low-income and middle-income countries: a systematic review and meta-analysis. *The Lancet Psychiatry* 2020;**7**:851–64. doi:10.1016/S2215-0366(20)30256-X
66 Pettifor A, MacPhail C, Selin A, *et al.* HPTN 068: A Randomized Control Trial of a Conditional Cash Transfer to Reduce HIV Infection in Young Women in South Africa—Study Design and Baseline Results. *AIDS Behav* 2016;**20**:1863–82. doi:10.1007/S10461-015-1270-0/FIGURES/3
67 Faul F, Erdfelder E, Lang AG, *et al.* G*Power 3: A flexible statistical power analysis program for the social, behavioral, and biomedical sciences. *Behav Res Methods 2007 392* 2007;**39**:175–91. doi:10.3758/BF03193146

Figure 1
CONSORT Flow Diagram for the DoBAt Pilot Trial

For peer review only

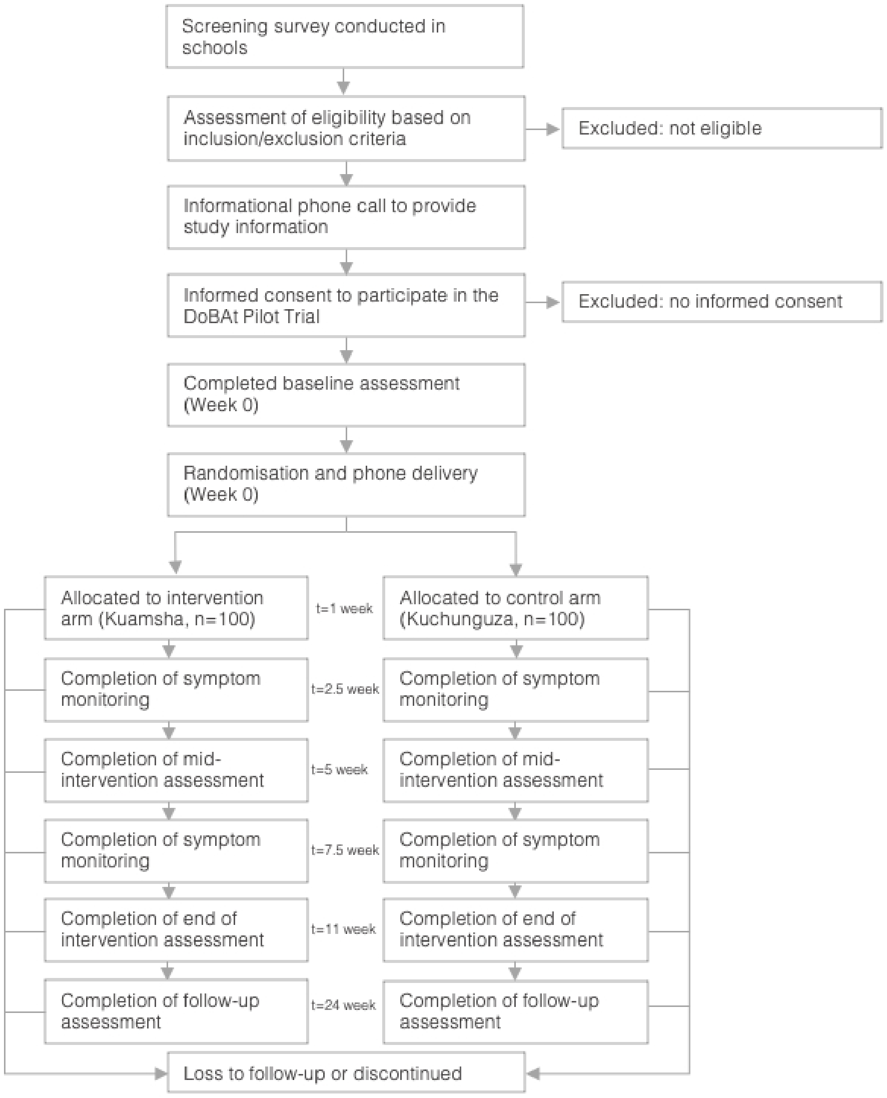

Figure 1. CONSORT Flow Diagram for the DoBAt Pilot Trial

232x263mm (72 x 72 DPI)

SUPPLEMENTAL MATERIAL

Digital delivery of Behavioural Activation therapy to overcome depression and facilitate social and economic transitions of adolescents in South Africa (the DoBAt study): protocol for a pilot randomised controlled trial

Table of Contents

Table 1. Trial Personnel and Committees 2
Table 2. The Song Contest story 3
Table 3. The Football Match story 4
Table 4. Example mock-ups of the Kuamsha app 5
Table 5. Trial registration data 7
Example of the Participant Consent Forms 9
DoBAt Study Statistical Analysis Plan 16

For peer review only

Table 1. Trial Personnel and Committees

Committee	Members and responsibilities
**Trial Management Team (TMT)**	Comprised of the co-Principal Investigators, the Trial Manager, the Trial Psychologist, and other investigators. The TMT is responsible for the day to day running of the trial and meets on a weekly basis.
**Risk Management Team (RMT)**	The RMT consists of the Trial Manager (a Medical Doctor), the Trial Clinical Psychologist, the Trial Registered Counsellor, and a senior Field Supervisor. Supervision and oversight are provided by Prof. Alan Stein (Child and Adolescent Psychiatrist), Prof. Kathleen Kahn (Medical Doctor), Prof. Crick Lund (Clinical Psychologist) and Prof. Tholene Sodi (Clinical Psychologist). The RMT is responsible for assessing and managing all risks amongst screened and enrolled participants.
**Trial Steering Committee (TSC)**	Consists of senior academic clinicians and researchers including Prof. Roz Shafran (chair of the TSC, Professor of Translational Psychology, UCL), Prof. Jonathan Roiser (Professor of Cognitive Neuroscience, UCL), Prof. Soraya Seedat (Professor of Psychiatry, Stellenbosch University), and Prof. Jonathan Levin (Professor of Biostatistics, University of Witwatersrand). The TSC oversees the scientific conduct of the study and meets on a quarterly basis throughout the trial.
**Independent Data and Safety Monitoring Board (DSMB)**	Comprised of Prof. Bonginkosi Chiliza (chair of the DSMB, Chief Specialist and Head of the Department of Psychiatry at the University of KwaZulu-Natal), Dr. Elizabeth George (Statistician, MRC Clinical Trials Unit at University College London), Prof. John Joska (HIV Mental Health Research Unit, Division of Neuropsychiatry, University of Cape Town), and Prof. Marguerite Schneider (Department of Psychiatry and Mental Health, University of Cape Town). The DSMB oversees the conduct and safety of the trial. Meetings will be held before recruitment begins, midway through recruitment, and at the end of the 11-week assessment period. A formal interim analysis will not be conducted.

For peer review only

For peer review only - http://bmjopen.bmj.com/site/about/guidelines.xhtml

Table 2. The Song Contest story

Learning module	Description of each module	BA Learning principles
**Episode 1 ("Pick a team")**	Students find out about a school Song Contest. The winning prize is a voucher to shop for a new outfit at the mall. User chooses the main character's name and picks two teammates to join the Song Contest.<br>Main character gets anxious about the idea of performing in front of everyone else. Christine (teacher) talks about the benefits of stepping outside one's comfort zone and offers her support.<br>At the end of the module, the user will be asked to set a goal to work on over the 10 weeks of intervention.	Absorption<br>TRAP-TRAC<br>Sleep<br>Self-confidence<br>Relapse prevention
**Episode 2 ("Find a cool tune")**	First practice session with group which didn't go well because one teammate fell asleep. Main character is frustrated and decides to ask Christine for advice. After hearing that teammate's grandmother ill, main character showed leadership and compassion in how they handled the situation.	
**Episode 3 ("The Lure")**	The team worked well together and made progress on the song. At the end of the practice, Prince (desirable character) invites the main character to go to the tavern. Main character is asked to think about the consequences of their actions but decides to go anyway.	
**Episode 4 ("The Fallout")**	The next day the main character is exhausted, hungover, and unable to concentrate. They arrive late to practice session without homework done. Teammates get annoyed. Main character decides to apologise instead of avoiding the problem and their teammates are forgiving. Main character learns about the importance of sleep and goes to sleep early.	
**Episode 5 ("The Return")**	Main character sleeps and feels recharged. Team is happy with the practice. Song is completed. Main character learns that they have to give a presentation if they win and asks Christine for advice. Main character learns ways to deal with being nervous and how to feel more self-confident.	
**Episode 6 ("The Big Day")**	Team feels nervous before the show. They perform and win the prize. Group reflects on journey.	

For peer review only

Table 3. The Football Match story

Learning module	Description of each module	BA Learning principles
**Episode 1 ("Pick a team")**	Main character is the striker for the local team. User chooses the main character's name and picks a name for the football team. User gets introduced to the football task. At the end of the module, the user will be asked to set a goal to work on over the 10 weeks of intervention.	Absorption<br>Rumination<br>TRAP-TRAC<br>Problem-solving<br>Negotiation
**Episode 2 ("Game Over")**	Main character misses some important shots. Shane (antagonistic character) is a discouraging main character because of their poor performance. Team loses the game. Main character leaves the match alone feeling upset and discouraged, ruminating over her performance during the match. Bird offers different, more positive perspective on performance but the main character not in a frame of mind to believe it.	
**Episode 3 ("Hide away")**	Main character is frustrated, misses school and gets a detention. Main character feels a bit better after friends visit her/him at her house, but apprehensive about going to school. Main character plays the football task which makes them feel energised enough to do their homework.	
**Episode 4 ("That's okay")**	Main character returns to school feeling more positive after a good night's sleep. Main character handles Shane well, but their mood takes a dip. Main character decides to speak with Coach Bayer after school about their performance in the match. Main character learns that mistakes can be useful opportunities to learn, instead of something to avoid. Main character practises what they've learnt and starts to feel more confident.	
**Episode 5 ("Keep practising")**	Main character speaks with friends before practice about what they learnt from Coach Bayer and asks for help in dealing with Shane. With their supportive presence, main character gets Shane to agree to stay away from them so that they can focus on football and winning. Main character feels confident, keeps practising, and feels ready for the re-match.	
**Episode 6 ("The Big Match")**	It all came together for the main character, stepping out onto the pitch with new confidence. Main character keeps calm and meets their mistakes with curiosity instead of frustration. Main character scores the winning goal.	

For peer review only

Table 4. Example mock-ups of the Kuamsha app

Component	Description	Example
Home screen	This is the first screen that users will see as they open the Kuamsha app. Users will have the option to play through the stories, monitor their mood, play absorbing activities to improve focus, or report on their weekly activities (see below for further details on each of these components).	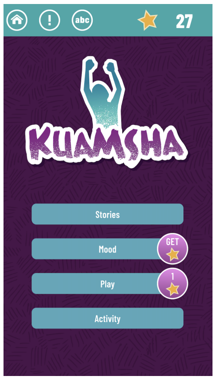
Log-in unlock code	Kuamsha is password-protected. Users will be asked to enter a password every time they access the app.	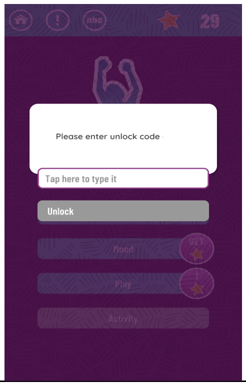
Language selector	Users will be able to select their preferred language. All the text in the app underwent two rounds of translation and has been checked by a clinical psychologist for accuracy.	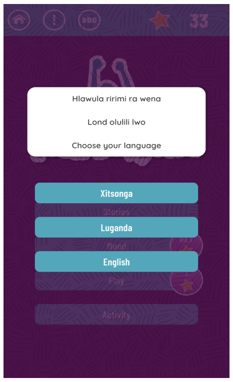
Story Selection	The core of the game consists of a choice between two narrative stories. Each consists of 6 modules that are played in sequential order. It is possible to begin one story and then switch to the other. During gameplay points are earned for the choices made and by completing other core game elements described below.	

For peer review only

For peer review only - http://bmjopen.bmj.com/site/about/guidelines.xhtml

<table>
<tr><td></td><td></td><td>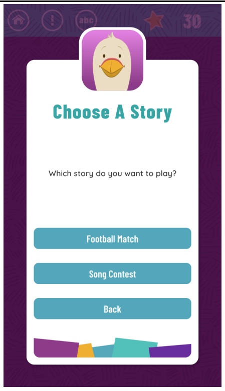
</td></tr>
<tr><td>Mood monitoring</td><td>Mood monitoring is an important part of BA and can be used as a tool for understanding how your mood is impacted and changes depending on the activities you are engaged in. Every time a user complete mood monitoring, they receive in-app points. Participants are asked to monitor their mood at various times (before and after each module, when they report a homework activity, and when they complete an absorbing activity).</td><td>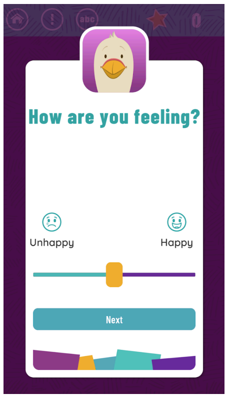
</td></tr>
</table>

For peer review only

Table 5. Trial registration data

Data category	Information
**Primary registry and trial identifying number**	South African National Clinical Trials Registry (DOH-27-112020-5741); Pan African Clinical Trials Registry (PACTR202206574814636)
**Date of registration in primary registry**	19th November 2020
**Ethics reference**	MED20-05-011 / OxTREC 34-20
**Protocol version**	v1.2 19/04/2021
**Protocol amendments**	Important protocol amendments such as changes to eligibility criteria, outcomes or analysis will be reported to investigators, the TSC, and both ethics committees in writing, and relayed to study participants at the soonest availability.
**Funder**	MRC Newton Fund UK-South Africa Joint Initiative on Mental Health (MR/S008748/1)
**Sponsor**	Wits Health Consortium (Pty) Limited, University of Witwatersrand<br>31 Princess of Wales Terrace, Parktown, Johannesburg, 2193<br>011 274 9200
**Trial Title**	Digital delivery of Behavioural Activation therapy to overcome depression and facilitate socio-economic transitions of adolescents in South Africa: pilot Randomised Control Trial
**Short Title**	DoBAt Study
**Countries of recruitment**	South Africa
**Health condition(s) or problem(s) studied**	Depression
**Intervention**	All participants in the intervention and control arms will be given a Samsung Galaxy A2 Core Android smartphone and receive active symptom monitoring via text messages sent to the smartphone every 2.5 weeks.<br>• The intervention arm will receive Behavioural Activation (BA) therapy via a smartphone application (the *Kuamsha* app) and supported by weekly phone calls from Trained Peer Mentors, implemented over 10 weeks.<br>• The control arm will receive a smartphone application (the *Kuchunguza* app) containing six module video clips from WildEarth-SafariLive, a locally produced wildlife series.
**Trial Participants**	Adolescents aged 15-19 with mild to moderately-severe depression based on scores between 5 and 19 on the 9-item Patient Health Questionnaire-Adolescent version (PHQ-A), that live in the MRC/Wits-Agincourt study area, Bushbuckridge, and from whom we obtain written informed assent and consent (including parental consent if <18 years).
**Study type**	Two-arm single-blind individually randomised controlled pilot trial
**Date of first enrolment**	25th November 2021
**Trial Status**	Enrolment of the first participant occurred on the 25th November 2021. We expect to finish data collection in January 2023.
**Target sample size**	200
**Recruitment status**	Recruiting
**Primary outcomes**	1) to determine the feasibility and acceptability of the intervention<br>2) to provide preliminary evidence on the initial efficacy (direction and magnitude) of any effects of the intervention on depressive symptoms
**Key secondary outcomes**	1) to pilot a range of mental health, social-affective cognition, risky behaviours, and socioeconomic measures

For peer review only - http://bmjopen.bmj.com/site/about/guidelines.xhtml

	2) to collect descriptive data on trial procedures such as recruitment, retention, data collection, randomisation and blinding to inform the development of a further larger trial.
**Data sharing statement**	Individual participant quantitative data that underlie the results reported in each publication arising from the trial will be made available after deidentification. Any video, audio or qualitative data will not be available. Data will be available beginning 9 months and ending 36 months following main article publication to researchers who provide a methodologically sound proposal that purposes to achieve aims in the approved proposal and /or for individual participant data meta-analysis. Data is documented and stored on the MRC/Wits-Agincourt Data Repository with a digital object identifier (doi) and can be accessed with permission and in line with with MRC/Wits-Agincourt policies and procedures. Data requestors will need to sign a data access agreement before any data can be shared. In addition, Study Protocol and Statistical Analysis Plan documents will be available.

For peer review only

For peer review only - http://bmjopen.bmj.com/site/about/guidelines.xhtml

Example of the Participant Consent Forms

PARTICIPANT INFORMATION SHEET: ADOLESCENTS UNDER 18 YEARS OLD

Study title: Digital delivery of Behavioural Activation therapy to overcome depression and facilitate social and economic transitions of adolescents in South Africa (DoBAt study)

Good day,

I am Mr/Mrs____ from the MRC/Wits-Agincourt Research Unit.

We would like to ***invite*** you to take part in a research study. Please read this carefully. **Ask us** if there is anything you do not understand and **take time** to decide whether or not you want to participate. It is important that you understand what it will mean if you take part, including what could go wrong. You can talk about this with your family, friends, parents, or anyone else you want to. You may not take part in another study while you take part in this study. Please read through this sheet and ask the study team about anything that you do not understand.

1. What is the study about?

We are doing research on adolescents' wellbeing. In this study we want to see whether it is possible to improve low mood, or symptoms of depression, by using a smartphone application (app) combined with phone calls from a peer mentor. The app will be set up like a game and teach ideas based on Behavioural Activation (BA), a strategy to help treat low mood. A peer mentor is someone who speaks Xitsonga and is a few years older than you.

2. Why have I been invited to take part?

You have been invited to take part because you recently participated in a survey at your high school about your thoughts, feelings and behaviours. Based on your answers, we think you may be experiencing low mood, which is one of the things we are looking at in this study.

In order to take part in the study, you must be aged 15-19, enrolled in Grades 9-11 and speak and read Xitsonga. You must also live in the local area for at least 12 weeks after the study begins.

You can't take part in the study if you have a problem with your health that would make it hard to participate. You also can't take part if you are seeing someone (like a counsellor or doctor) for problems with your mood or mental health already. You can ask the researcher about this if you're not sure.

3. How big is the study and how long will it last for?

There will be about 200 adolescents taking part. The study will take place during 2021 and 2022. If you decide to participate, you would be involved for 6 months.

4. What is involved for me?

If you are interested in taking part in the study, a researcher will arrange a time to come to your house. They will go over information about the study with you and your parent/guardian. You can ask any questions you have about the study at any point. After this, if you decide to take part, you will be asked to sign an electronic form to say that you agree to participate (an *assent form*). Because you are under 18, your parent/guardian will also need to give permission by signing a *consent form* before you can participate. The researcher will ask you some questions about your health and wellbeing to check that you are able to participate in the study. They will also ask you to answer some questions and do some tasks to measure

For peer review only - http://bmjopen.bmj.com/site/about/guidelines.xhtml

your thoughts, feelings and behaviours (see more on 'assessments' below). We will also ask your parent/guardian some questions about your household.

After this, the researcher will find a time to come back to your house to give you the smartphone. The smartphone will have an app installed which you will use during the study. The researcher will show you how to use the phone and the app, and give you a chance to ask any questions. They will also tell you who to ask for help if you have a problem with the smartphone or the app, or if your mood is very low.

Once you are signed up for the study, you will find out whether you are in Group 1 (Kuamsha app, the type of support we are testing) or Group 2 (Kuchunguza app, more on this below). You have a 50/50 chance of getting one or the other. You will then use the smartphone for 10 weeks. What you do with the smartphone depends on which group you are in:

Group 1 (Kuamsha app)
If you are in Group 1, your smartphone will have an app on it. The app will be set up like a game and teach ideas based on Behavioural Activation (BA), a strategy that teaches skills to cope with your problems and low mood. The app is like a game and you can play through six 'episodes' which are each part of a story. Each episode will take around 10 minutes and you will get some weekly activities to work on at the end. The weekly activities will take around 20 minutes and involves different activities like going for a walk, setting up a bedtime routine, or listening to music. The app will also ask you to monitor your mood at different times during the study. The researcher will also put you in touch with a peer mentor, which is someone a few years older than you who will help you with the app and the weekly activities. The peer mentor will keep your information private and confidential. The peer mentor will first call you for an 'introductory phone call', where you will get the chance to introduce each other and answer any questions you may have. After this phone call, you will speak to the peer mentor for 15-20 minutes for each episode that you finish.

Group 2 (Kuchunguza app)
If you are in Group 2, your smartphone will have an app with videos to watch (6 in total). Each video should take around 15-20 minutes to watch.

Assessments (Groups 1 and 2)
We will ask you questions at different points during the study. The first time (week 0) the researcher visits your house, they will ask you some questions on your thoughts, behaviours, past experiences, employment, consumption, and aspirations for the future. You will listen to some of these questions using headphones and click your answers on a tablet. This means that you won't have to say anything out loud (so your answers are private). You will also use the tablet to do some tasks which measure memory, learning, and decision-making. Some of these tasks will give you a chance to win mobile data. After 11 weeks (week 11), the researcher will come back to your house to do these questions again.

During the study, the researcher will also call you or send you a text message (SMS) every 2-3 weeks to ask you some questions about your symptoms. This will also allow us to check on how you are doing.

At the end of the study (week 24), the researcher will call you or send you a text message to ask you some of the questions you were asked at the beginning of the study. The researcher will ask some participants (but not everyone) to take part in a one-on-one interview on a separate occasion. If you are asked to take part in an interview, the researcher will ask you questions about what it was like participating in the study. This will last around 1 hour.

During the study, we will use information about your household which was collected by the MRC Wits-Agincourt Research Unit. This was collected during the census, also known as the Health and Demographic Surveillance System (HDSS).

The timeline shows what will take place in the study, when and where:

For peer review only - http://bmjopen.bmj.com/site/about/guidelines.xhtml

Timeline for Group 1:

WEEK 0	WEEK 1	WEEK 2	WEEK 3	WEEK 4	WEEK 5	WEEK 6	WEEK 7	WEEK 8	WEEK 9	WEEK 10	WEEK 11	WEEK 24
**Consent, Assent, Assessment 1** Home visit 2 hours<br>**Phone delivery** Home visit 1 hour<br>**Introductory Phone call Peer mentor** 15-20 mins	**Episode 1** Kuamsha app 10 minutes<br>**Peer mentor call 1** Phone call 15-20 minutes	**Episode 2** Kuamsha app 10 minutes<br>**Peer mentor call 2** Phone call 15-20 minutes<br>**Symptoms monitoring** SMS 5 minutes	**Episode 3** Kuamsha app 10 minutes<br>**Peer mentor call 3** Phone call 15-20 minutes		**Assessment 2** Phone/ electronically 30 minutes	**Episode 4** Kuamsha app 10 minutes<br>**Peer mentor call 4** Phone call 15-20 minutes	**Episode 5** Kuamsha app 10 minutes<br>**Peer mentor call 5** Phone call 15-20 minutes<br>**Symptoms monitoring** SMS 5 minutes	**Episode 6** Kuamsha app 10 minutes<br>**Peer mentor call 6** Phone call 15-20 minutes			**Assessment 3, Interview** Home visit 2 hours	**Assessment 4** Phone/ electronically 1 hour

Timeline for Group 2:

WEEK 0	WEEK 1	WEEK 2	WEEK 3	WEEK 4	WEEK 5	WEEK 6	WEEK 7	WEEK 8	WEEK 9	WEEK 10	WEEK 11	WEEK 24
**Consent, Assent, Assessment 1** Home visit 2 hours<br>**Phone delivery** Home visit 1 hour	**Video 1** Kuchunguza app 20 minutes	**Video 2** Kuchunguza app 20 minutes<br>**Symptoms monitoring** SMS 5 minutes	**Video 3** Kuchunguza app 20 minutes		**Assessment 2** Phone/ electronically 30 minutes	**Video 4** Kuchunguza app 20 minutes	**Video 5** Kuchunguza app 20 minutes<br>**Symptoms monitoring** SMS 5 minutes	**Video 6** Kuchunguza app 20 minutes			**Assessment 3, Interview** Home visit 2 hours	**Assessment 4** Phone/ electronically 1 hour

5. Do I have to participate?

No. It is up to you whether you decide to take part. If you decide that you don't want to take part, that's okay. You can decide that you don't want to take part at any time – before the study, during the study, or afterwards. You don't need to give a reason, and no one will be upset or annoyed. Deciding not to take part won't affect your grades, school, medical care or any other services. You may discuss whether or not to take part with your family, friends, or teachers.

6. Will taking part cause me any problems?

Whilst we hope that participating in the study is a positive experience for you, we know that some of the questions might make you feel upset or frustrated. If any question makes you uncomfortable, you can skip the question. If you feel upset by anything in the study you can talk to the research staff who will do their best to help you feel better.

We also realise that in some places people can have negative ideas around people who have low mood. We will make sure to keep this information very private and confidential so that no one outside of the study team and your parent/guardian knows you are participating in the study.

We think it is very unlikely that you will suffer any harm from taking part in the study, but it is good to know that the University of Witwatersrand has insurance in case anything did happen to you.

7. Will taking part help me at all?

We know that Behavioural Activation (BA) can sometimes improve young people's mood. We're not sure whether it would improve your mood or not, because it is one of the things we are testing in the study. Your participation in the study will also help us to understand the needs of adolescents with low mood and to develop a treatment that works for them.

For peer review only - http://bmjopen.bmj.com/site/about/guidelines.xhtml

8. What happens to my information? Will it be kept confidential?

All the information you share with us is private and confidential. That means that we would not name you or tell your parents, friends, or teachers what you said when we meet. This includes your answers to the questions, what you say during the phone calls and interviews. We will also take out any information that would identify you and keep all of the information about you safely in a different place to your answers to the study questions. Your information will be stored on a password protected computer for at least 10 years. If you stop taking part, we will automatically destroy any data related to you, unless you give us permission not to.

The only times we would break our confidentiality is if we were worried that you – or someone in contact with you – was at risk of serious harm. If that happens, we would talk with you about it so that we could make a plan together to get you the help and support you need. We might also need to break the confidentiality if it was required by law or by the Human Research Ethics Committee at the University of the Witwatersrand.

If you are in Group 1 and receive calls from the peer mentor, we will record the peer mentor calls. We will also record the interviews that happen in Week 11, if you are asked to do one. We will write down what was said, taking care to take out your name and any other information which could identify you. Once we write everything down, we will delete the recordings. We might include quotes of what you say when we publish results of the study, but we will make sure that no one can tell who said them. You will be able to choose a name that we can use instead of your real name (a *pseudonym).*

9. Will I have to pay for anything? Will I be paid to take part?

You do not have to pay to take part. You will also not be paid. As part of the study, we will give you a smartphone to use, which you can keep at the end of the study. We will cover the cost of mobile data for you to use the app. We will also offer you a snack and a small amount of mobile data when we visit your home to do the questionnaires. You will also receive 10ZAR mobile data each time you complete a questionnaire by text message (SMS).

If you are in Group 1, the peer mentor will call you so that you do not have to use your own airtime.

10. Will we publish the results of this study?

Yes, we will publish the results of this study in academic journals. We will also provide ‘fact sheets’ with information about the results of the study at community meetings and to service providers, village leaders and other people locally. We will publish only findings that are important and apply to many people, and we will not publish any information that would identify you. You can get in touch with us if you would like us to tell you the results of the study directly.

11. Who has approved this study?

This study has been approved by the Human Research Ethics Committee (Medical) of University of Witwatersrand, Ehlanzeni District and Mpumalanga Provincial Departments of Health and Education, and the Oxford Tropical Research Ethics Committee. The purpose of these committees is to protect the integrity (honesty) of the research, and the rights and dignity of all people who agree to participate in a research project.

12. Who can I talk to for more information or to report a problem?

If you have any questions about this study or you are worried about something, please call us:

- Professor Kathy Kahn, Principal Investigator, tel: 011 717 2617, email: Kathleen.Kahn@wits.ac.za
- Dr Bianca Moffett, Project Manager, email: Bianca.Moffett@wits.ac.za.
- If you need assistance in Xitsonga, you can call this number: 0824068527

For peer review only - http://bmjopen.bmj.com/site/about/guidelines.xhtml

Within 10 days, they should say that they have received your concern and tell you how they are thinking of dealing with it.

If you are feeling upset or distressed, you can also contact Sindile Hlatswayo, the social worker at Agincourt CHC, on 013 708 1471/7940 during office hours, or book an appointment to see the clinical psychologist at Mapulaneng Hospital by contacting 013 799 0214.

If you have any concerns about the way the study is being conducted, please contact the Chairperson of the University of the Witwatersrand Ethics Committee, Dr Clement Penny, tel: 011 717 2301, email: Clement.Penny@wits.ac.za. The telephone numbers for the Ethics Committee secretariat are 011 717 2700/1234 and the e-mail addresses are Zanele.Ndlovu@wits.ac.za and Rhulani.Mukansi@wits.ac.za.

13. Data protection

The University of the Witwatersrand is responsible for the way your data is used in the study. They will process your data for this research, which is performed in the public interest. More information about your rights with regard to personal data can be seen online at: https://compliance.web.ox.ac.uk/individual-rights.

Thank you for reading this Study Information Sheet.

Date:

For peer review only - http://bmjopen.bmj.com/site/about/guidelines.xhtml

ASSENT FORM: ADOLESCENTS UNDER 18 YEARS OLD

Study title: Digital delivery of Behavioural Activation therapy to overcome depression and facilitate social and economic transitions of adolescents in South Africa (DoBAT)

1. Have you had enough time to read the Participant Information Sheet or has someone explained this study to you?	Yes / No
2. Do you understand what this study is about?	Yes / No
3. Have you asked all the questions you want?	Yes / No
4. Do you understand it's okay to stop taking part in this study at any time?	Yes / No
5. Do you understand that you will not receive any payment to be in the study and that participating in the study will not cost you anything but your time?	Yes / No
6. Do you understand who will have access to your information, how it will be stored, and what will happen to it at the end of the study?	Yes / No
7. Do you understand that we will keep all your information anonymous and confidential?	Yes / No
8. Do you understand that your parent/legal guardian will also need to give permission for you to take part in this study?	Yes / No
9. Do you understand that you can contact the research team if you have any questions?	Yes / No
10. Are you happy to take part?	Yes / No

For peer review only

PARTICIPANT

________________	________________	________________	________________
Name of participant	Signature or mark	Date and time	Phone number (if available)

STUDY TEAM

________________	________________	________________
Name of person taking consent	Signature or mark	Date and time

For peer review only - http://bmjopen.bmj.com/site/about/guidelines.xhtml

AUDIO RECORDING

As mentioned on the information sheet, we may ask you to do an interview after 3 months, which will be recorded. If you are in Group 1, we will also record the conversations you have with the peer mentor. We will type up this information as soon as possible and then delete the recording. All the information you say with us is private and confidential. We may use quotes of what you say when we write about the study, but we will take care to take out any information which could identify you. We will keep all of the information about you safely and in a different place to the recording.

Are you happy for your voice to be recorded?	Yes / No

PARTICIPANT

______________________ ____________ ____________________
Name of participant Date and time Signature or mark

STUDY TEAM

______________________ ____________ ____________________
Name of person taking consent Date and time Signature

Researcher contact details:

- Professor Kathy Kahn, Principal Investigator, tel: 011 717 2617, email: Kathleen.Kahn@wits.ac.za.
- Dr Bianca Moffett, Project Manager, email: Bianca.Moffett@wits.ac.za.
- If you need assistance in Xitsonga, you can call this number: 0824068527
- Dr CB Penny, Chairperson of the Human Research Ethics Committee (Medical) at the University of Witwatersrand, tel: 011 717 2301, e-mail: Clement.Penny@wits.ac.za
- Ms. Zanele Ndlovu or Mr. Rhulani Mkansi, Committee Secretariat, tel: 011 717 2700/1234, e-mail: Zanele.Ndlovu@wits.ac.za or Rhulani.Mkansi@wits.ac.za

For peer review only

For peer review only - http://bmjopen.bmj.com/site/about/guidelines.xhtml

DoBAt Study Statistical Analysis Plan

Statistical Analysis Plan

PILOT TRIAL FULL TITLE	Digital delivery of Behavioural Activation therapy to overcome depression and facilitate social and economic transitions of adolescents in South Africa<br>(The DoBAt study)
SAP VERSION	Version 1.0
SAP VERSION DATE	3rd November 2022
PILOT TRIAL STATISTICIAN	Prof. Eustasius Musenge<br>School of Public Health<br>University of the Witwatersrand
Protocol Version (SAP associated with)	Version 1.2 (19/04/21)
PILOT TRIAL PRINCIPAL CO-INVESTIGATOR	Prof. Kathleen Kahn, University of the Witwatersrand<br>Prof. Alan Stein, University of Oxford
SAP AUTHOR(s)	Ms. Julia Ruiz Pozuelo (lead author)<br>Dr. Kate Orkin<br>Prof. Eustasius Musenge

For peer review only - http://bmjopen.bmj.com/site/about/guidelines.xhtml

Abbreviations

AE	Adverse Event
BA	Behavioural Activation
EF	Executive Function
DSMB	Data and Safety Monitoring Board
ESoC	Enhanced Standard of Care
ITT	Intention to treat population
MRC	Medical Research Council
PHQ-A	Patient Heath Questionnaire Adolescent version
SAE	Serious Adverse Event
SAP	Statistical Analysis Plan
TSC	Trial Steering Committee

For peer review only

For peer review only - http://bmjopen.bmj.com/site/about/guidelines.xhtml

1. Introduction

This document details the proposed presentation and analysis for the final trial report presenting the results from an MRC-funded pilot randomised controlled trial of a digital intervention for adolescents with depression in the rural Bushbuckridge Local Municipality of Mpumalanga Province, South Africa.

This document describes the objectives, intervention, core pre-registered hypotheses we commit to testing, the definitions of the outcomes of interest, and estimation and inference methods. We do not rule out the possibility of running other estimations. However, we will make clear in the papers which estimations are specified in this pre-analysis plan and which are not.

We have not yet completed recruitment at the time of lodging this analysis plan.

2. Background information

2.1 Rationale

Depression peaks during adolescence, with up to 20% of adolescents affected in rural South Africa and with few having access to treatment (Goin et al., 2019; Kessler et al., 2005). Untreated depression exerts a substantial economic toll as it impairs functioning, interferes with schooling, and impacts adolescents' ability to build successful relationships and make important life decisions (Thapar et al., 2012). These impairments have a greater impact on adolescents in low- and middle-income countries due to the additional adversities they face and the lack of available, effective treatments. Evidence is urgently needed for cost-effective and scalable interventions targeting adolescent depression.

2.2 Study Objectives

In this pilot study, we are exploring if a smartphone app that delivers Behavioural Activation (a psychological therapy for depression) can reduce depressive symptoms among adolescents living with depression in the Bushbuckridge area.

The co-primary objectives of the study are:
1) to determine the feasibility and acceptability of a digitally delivered Behavioural Activation therapy intervention for adolescents living with depression in the Bushbuckridge sub-district of Mpumalanga province, South Africa; and
2) to provide preliminary evidence on the efficacy (direction and magnitude) of any effects of the intervention on depressive symptoms amongst adolescents in the intervention arm compared to the control group.

The secondary objectives are:
1) to pilot locally adapted outcome measures of mental health, social-affective cognition, risky behaviours, and socioeconomic measures; and
2) to collect descriptive data on trial procedures such as recruitment, retention, data collection, randomisation, and blinding to inform key parameters in developing a future large-scale trial.

2.3 Trial reporting

This protocol is reported in accordance with the Standard Protocol Items: Recommendations for Intervention Trials (SPIRIT) 2013 statement (Chan et al., 2013). The final analysis for the main

For peer review only - http://bmjopen.bmj.com/site/about/guidelines.xhtml

results will be conducted once all the end-of-intervention assessments (week 11) have been completed.

2.4 Trial committees

Trial Steering Committee

The Trial Steering Committee (TSC) consists of Prof. Roz Shafran (chair of the TSC, Professor of Translational Psychology, UCL), Prof. Jonathan Roiser (Professor of Cognitive Neuroscience, UCL), Prof. Soraya Seedat (Professor of Psychiatry, Stellenbosch University), and Prof. Jonathan Levin (Professor of Biostatistics, University of Witwatersrand). The TSC oversees the scientific conduct of the study and meets on a quarterly basis.

Data and Safety Monitoring Board

The Data and Safety Monitoring Board (DSMB) is comprised of Prof. Bonginkosi Chiliza (chair of the DSMB, Chief Specialist and Head of the Department of Psychiatry at the University of KwaZulu-Natal), Dr Elizabeth George (Statistician, MRC Clinical Trials Unit at University College London), Prof. John Joska (HIV Mental Health Research Unit, Division of Neuropsychiatry, University of Cape Town), and Prof. Marguerite Schneider (Department of Psychiatry and Mental Health, University of Cape Town). The DSMB oversees the protection of participant safety. Meetings will be held before recruitment begins, midway through recruitment, and at the end of the 11-week assessment period. A formal interim analysis will not be conducted.

3. Intervention

All participants in the intervention and control arms will be given an entry-level Samsung Galaxy A2 Core Android **smartphone**, which they can keep at the end of the study. Furthermore, participants in both groups 200MB of **mobile internet data** at six different points (0, 2.5, 5, 7.5, 11, and 24 weeks) to ensure they have data to use the app and complete the online surveys.

Participants in both arms will receive **active symptom monitoring** via text messages sent to the smartphone every 2.5 weeks. Any adolescents who develop severe depression or high-risk suicidal ideation will be assessed by the Risk Management Team and referred to local clinical services. Adolescents started on anti-depressant medication or receiving psychological therapy because of these referrals will not be discontinued (we will note concomitant care and examine this using sensitivity analyses).

3.1 Control arm: Enhanced Standard of Care (ESoC)

The non-intervention arm will receive a control app (the *Kuchunguza app*) containing six video clips from *WildEarth-SafariLive*, a locally produced wildlife series. Each video clip takes approximately 15-20 minutes to complete and allows users to explore the African wilderness while listening to calming and atmospheric sounds.

In the given context, the control represents an enhancement of standard care since most adolescents with depression would not usually receive any intervention or active symptom monitoring and referral.

3.2 Intervention arm: Kuamsha programme

For peer review only - http://bmjopen.bmj.com/site/about/guidelines.xhtml

The intervention arm will receive six modules of Behavioural Activation (BA) therapy via a smartphone application (the *Kuamsha app*) supported by trained Peer Mentors, implemented over ten weeks. The app and phone calls from the Peer Mentors comprise the Kuamsha programme.

The Kuamsha app is primarily an interactive narrative game comprising six tailored modules (sessions) that contain Behavioural Activation's core principles integrated into a gamified format.[1] Each module takes approximately 15-20 mins to complete. They cover topics such as identifying and engaging in meaningful activities, and using strategies to overcome barriers, for example, problem-solving, effective communication, getting enough sleep, and disengaging from rumination. Each module is followed by a homework activity where the participant is encouraged to reflect on the BA principles outlined in each module and think about ways to apply them to their own lives. Participants will be asked to report how often they completed the homework activities and their mood as they did them. The app includes game design elements to stimulate motivation and performance, including character personification, in-app points, and reminders/ notifications. The Kuamsha app has been developed through extensive formative research and iterative user-centred design with adolescents in the study area to increase usability and acceptability.

The Kuamsha app will be supplemented by brief phone calls (15 mins per module) from Trained Peer Mentors. There will be seven calls in total, including an introductory phone call and six calls to cover the content of each module. Peer Mentors will attempt to reach participants by calling up to 5 times per week. The role of the Peer Mentors is mainly to support adherence and compliance with the app and to troubleshoot problems related to the use of the app and implementation of the homework activities. They will be trained not to provide additional advice or counselling and conduct their calls according to a pre-determined checklist of activities to help ensure fidelity. Peer Mentors will be Xitsonga-speaking students or recent graduates from the department of psychology or social work at an accredited South African university. They will be trained and supervised by the Trial Psychologist according to the training manual developed specifically for the intervention.

Table 1 shows DoBAt's time schedule of enrolment, interventions, assessments, and visits for participants.

4. Study methods

4.1 Study design

The DoBAt study is a two-arm single-blind individual-level randomised controlled pilot feasibility trial. A total of 200 adolescents will be recruited (1:1 allocation ratio).

4.2 Recruitment and study timeline

Participants will be recruited through a two-stage recruitment process. The first stage of recruitment (Phase 1) will consist of a screening survey conducted in schools to identify adolescents with symptoms of mild to moderately severe depression. The second stage of recruitment (Phase 2) will be done with adolescents who score between 5 and 19 on the Patient Health Questionnaire Adolescent version (PHQ-A) and who also meet the other eligibility criteria.

---

[1] See (Jacobson et al., 2006) for a description of Behavioural Activation.

For peer review only - http://bmjopen.bmj.com/site/about/guidelines.xhtml

The pilot trial consists of an 11-week intervention treatment phase, and participants will be followed up for a further 13 weeks after completing the intervention (i.e., a total of 24 weeks). Primary outcomes will be assessed at the end-of-intervention assessment (week 11) by blinded outcomes assessors, and qualitative interviews will be conducted with a sub-sample of participants. Table 1 indicates the time schedule of enrolment, interventions, assessments, and visits for participants, and Figure 1 shows the flow of participants.

4.3 Study setting

The study will be based in the Bushbuckridge sub-district of Mpumalanga Province, South Africa. Since 1992 the MRC/Wits Rural Public Health and Health Transitions Research Unit (MRC/Wits-Agincourt) has collected population data, with vital events (pregnancy outcome, deaths, in- and out-migration) and household composition updated annually through its Health and socio-Demographic Surveillance System (HDSS). The total population under surveillance is currently ~116,000 inhabitants residing in 31 contiguous villages (Kahn et al., 2012).

4.4 Eligibility criteria

To be eligible for inclusion in the pilot trial participants must: 1) be between 15 and 19 years of age and in grades 9 to 11 at the beginning of the study; 2) have symptoms of mild to moderately-severe depression indicated by a score between 5 and 19 on the Patient Health Questionnaire Adolescent Version (PHQ-A); 3) be able to read sufficiently in the local language (Xitsonga) to use the Kuamsha app; 4) intend to continue living in the study site for 12-weeks after the baseline assessment; and 5) provide written informed assent/consent to participate in the study, as well as parent/guardian consent if younger than 18 years.

Participants will be excluded if they: 1) have symptoms of severe depression as indicated by a score of >19 on the PHQ-A; 2) have current suicidal ideation with specific plans and means identified; 3) are receiving psychological treatment for a mental health condition at the time of enrolment; 4) have been hospitalised for at least five days for a severe psychiatric illness (specifically Bipolar Mood Disorder, Schizophrenia or other Psychotic Disorders) or life-threatening or other serious medical illness; 5) have a history of Bipolar Mood Disorder, Schizophrenia or other Psychotic Disorders; 6) lack capacity to consent to research participation. Participants excluded for points 1) and 2) will be assessed by the Risk Management Team and referred to local clinical services as per prior arrangements with local service providers.

4.5 Allocation

Following the baseline assessment, participants will be randomly assigned to the intervention or control arm with a 1:1 allocation using a computerised minimisation algorithm, balanced by sex (male or female) and severity of depressive symptoms (<10 on the PHQ-A or ≥10 on the PHQ-A). Both variables will be collected during the first stage of recruitment (Phase 1). The minimisation algorithm was generated by the Centre for Healthcare Randomised Trials (CHaRT) at the University of Aberdeen. Participants will be allocated using CHaRT's online software and the trial manager will oversee enrolling participants as per assignment.

4.6 Blinding

This is a single-blind study, with all fieldworkers conducting outcomes assessments and other investigators remaining blinded.

For peer review only - http://bmjopen.bmj.com/site/about/guidelines.xhtml

Participating adolescents will be unblinded. For practical and risk management reasons, *Field Supervisors* will be aware of which adolescents have been allocated to intervention and control groups, but they will not be involved in any outcome assessments. Similarly, the *Project Manager and Risk Management Team* will not be blinded for the purposes of the pilot trial, to prioritise participant safety.

Any breaches in blinding will be documented, and we will endeavour to change the fieldworker conducting subsequent assessments on a particular adolescent where unblinding has occurred.

4.7 Sample size determination

The sample size was calculated on the basis of one of our co-primary objectives which aims to provide preliminary evidence of signals of efficacy (direction and magnitude) of any effects of the intervention on depressive symptoms (measured by the PHQ-A) amongst adolescents in the intervention arm compared to the control group.

Statistical power was calculated to detect differences between two independent groups, in a two-sided test with an α of 0.05 and a power of $1–\beta=0.80$, for an effect size (Cohen's d) of 0.45. This effect size is based on findings from previous studies investigating digital psychological interventions, with effect size (Cohen's d) ranging from 0.24 to 0.57 (Arjadi et al., 2018; Fu et al., 2020; Lehtimaki et al., 2021). Given these findings, we chose a small-to-medium effect size of 0.45 to account for the limited number of studies conducted with our target population and in our study setting. We allowed for 25% attrition, based on a previous study with adolescents in the Agincourt setting (Pettifor et al., 2016). Given these assumptions, we aimed to recruit 200 participants at baseline. We calculated the required sample size using G*Power software, version 3.1.9.3 (Faul et al., 2007).

5. Data collection

5.1 Data collection schedule

There are 4 planned assessments for this pilot trial, including the baseline assessment and 3 follow-up assessments (at week 5, 11, and 24). More information can be found in Table 1.

5.2 Description of measures

Primary outcomes

(1) Feasibility and acceptability of the intervention. A mixed methods approach will be adopted to establish feasibility and acceptability.

- **Feasibility** will be assessed by collecting data on the following:

  (1) Recruitment (enrolment rate of eligible participants) and retention in trial at the end of the intervention period (11 weeks).

  (2) Feasibility of testing procedures and data collection methods, including assessment of completion rates.

  (3) Treatment adherence rates, where adherence is defined as having opened at least 4 out of 6 of the app modules and as having completed 3 out of 6 phone calls with the peer mentor (excluding the introductory call). We will complement the treatment adherence rates with engagement metrics collected via the app (number of times participants logged into the app, number of modules opened and completed, total time spent on the app, number of weekly activities set to do, and number of times the participant completed the weekly activities).

For peer review only - http://bmjopen.bmj.com/site/about/guidelines.xhtml

- **Acceptability** of the intervention and study procedures will be assessed via:

  (1) An acceptability questionnaire conducted at the end of intervention assessment (week 11) with all participants. The questionnaire consists of three measures: Acceptability of Intervention Measure (AIM), Intervention Appropriateness Measure (IAM), and Feasibility of Intervention Measure (FIM) (Weiner et al., 2017). Each measure consists of 4 items. The score for each measure ranges from 1 to 5 and is calculated by assigning scores to the response categories [1=completely disagree to 5=completely agree] and calculating an average score for each of the measures. No items need to be reverse coded. Higher scores indicate greater acceptability, appropriateness or feasibility. We will inquire about the acceptability of the app using these three measures (to both intervention and control group) and the acceptability of the peer mentor programme (to the intervention group only).

  (2) In-depth interviews with a subsample of participants. We estimate that interviews with 20 participants will provide a sufficient range of experiences and perspectives to reach data saturation. This subsample will contain participants from both the treatment and control arms (2:1 ratio) and will be stratified based on high versus low app engagement (i.e., for each participant in the control group, we will have 2 participants in the treatment group, one with low engagement and another with high engagement).
- ***Fidelity of delivery of the intervention*** will be assessed by collecting data on adherence and competence of Trained Peer Mentors.

  (1) Adherence of the peer mentors is defined as the number of sessions that meet at least 90% of criteria for adherence according to the training protocol. Independent raters will listen to a random sample (10%) of recordings of Peer Mentors' phone calls with participants and assess them against the training protocol.

  (2) Competence of the peer mentors will be expressed as a percentage based on their Competency Assessment Test. This test will include a written test and observation of skills through role-playing to assess Knowledge, Attitudes and Practices. Tests will be scored by the Trial Psychologist using a pre-determined scoring system.

(2) Signals of initial efficacy on depressive symptoms

This will be assessed using the PHQ-A score at end of intervention assessment (week 11). The PHQ-A is a widely used and well-established measure of adolescent depressive symptoms over the past two weeks (Kroenke et al., 2001). The questionnaire consists of 9 items. The PHQ-A total score ranges from 0 to 27 and is calculated by assigning scores to the response categories [0=not at all, 1=several days, 2=more than half the days, 3-nearly every day] and summing the score for each of the items. Higher scores indicate greater severity of depression: 0-4 No or Minimal depression, 5-9 Mild depression, 10-14 Moderate depression, 15-19 Moderately severe depression, 20-27 Severe depression. This measure has shown good psychometric properties with a sample of adolescents and young adults in South Africa and Kenya (Bhana et al., 2015; Cholera et al., 2014; Osborn et al., 2019). Participants will be asked to complete this scale at screening, mid-

For peer review only - http://bmjopen.bmj.com/site/about/guidelines.xhtml

intervention (week 5), end of intervention (week 11), and follow-up (week 24). Participants will also be asked to complete this scale as part of the symptom monitoring (week 2.5 and week 7.5).

Secondary outcomes

We aim to pilot locally adapted measures of the concepts below to examine their acceptability and variation and inform the development of a further larger trial.

Mental health outcomes

Generalised Anxiety Disorder (GAD-7). The GAD-7 is designed to assess the symptoms of generalised anxiety disorder over the previous 2 weeks (Spitzer et al., 2006). The questionnaire consists of 7 items. The GAD-7 total score ranges from 0 to 21 and is calculated by assigning scores to the response categories [0=not at all, 1=several days, 2=more than half the days, 3-nearly every day] and summing the score for each of the items. Higher scores represent increased anxiety: 0-5 mild; 6-10 moderate; 11-15 moderately severe anxiety; 15-21 severe anxiety. Studies from Sub-Saharan Africa examining the psychometric properties of the GAD-7 have demonstrated good internal consistency and performance characteristics with young adults in Zimbabwe and among Kenyan youth (Chibanda et al., 2016; Osborn et al., 2019). Participants will be asked to complete this scale at baseline (week 0), mid-intervention (week 5), end of intervention (week 11), and follow-up (week 24).

Conor-Davidson Resilience Scale (CD-RISC). The CD-RISC measures the ability to cope with traumatic stress (Connor and Davidson, 2003). The questionnaire consists of 10 items. The CD-RISC total score ranges from 0 to 40 and is calculated by assigning scores to the response categories [0=not true at all, 1=rarely true, 2=sometimes true, 3=often true, 4=true nearly all the time] and summing the score for each of the items. Higher scores represent higher resilience. CD-RISC-25 has been used in a sample of South African adolescents in public schools (Jørgensen and Seedat, 2008) and with homeless youth in Ghana (Asante and Meyer-Weitz, 2014). Participants will be asked to complete this scale at baseline (week 0) and end of intervention (week 11).

Brief Rumination Response Scale (RRS). The brief RRS measures brooding, defined as a passive comparison of one's current situation with some unachieved standard (Treynor et al., 2003). The questionnaire consists of 5 items. The brief RRS total score ranges from 0 to 15 and is calculated by assigning scores to the response categories [0=Almost never, 1=Sometimes, 2=Often, 3=Almost always] and summing the score for each of the items. Higher scores represent higher rumination/brooding. The full scale has been previously used among depressed Nigerian adolescents (Olaseni, 2018) and it is currently being used in a trial led by Prof. Stein with HIV-positive pregnant women in KwaZulu-Natal. Participants will be asked to complete this scale at baseline (week 0), mid intervention (week 5), end of intervention (week 11), and follow-up (week 24).

Warwick-Edinburgh Mental Wellbeing Scale (WEMWBS). The WEMWBS is a questionnaire to assess psychological functioning and emotional wellbeing (Tennant et al., 2007). The questionnaire consists of 14 items. The WEMWBS total score for the full-scale ranges from 14-70 and is calculated by assigning scores to the response categories [1=None of the time, 2=Rarely, 3=Some of the time, 4=Often, 5=Nearly every day] and summing the score for each of the items. The 7-item scale SWEMWBS, raw scores will be transformed as described in the authors'

For peer review only - http://bmjopen.bmj.com/site/about/guidelines.xhtml

conversion table. The scale has been used in South Africa, Tanzania, and Kenya and proved to be valid in a range of settings (e.g., public health, schools, clinical settings, etc.). The Cronbach's alpha for the WEMWBS in a sample of Kenyan adolescents was 0.70, indicating adequate reliability of this measure (Osborn et al., 2019). Participants will be asked to complete the full-scale (WEMWBS, 14-items) at baseline (week 0) and end of intervention (week 11), and the short form (SWEWMBS, 7-items) at mid-intervention (week 5) and follow-up (week 24).

Behavioural Activation for Depression Scale (BADS). The BADS measures when and how individuals become less avoidant and more activated over the course of treatment (Kanter et al., 2007). The questionnaire consists of 25 items. The BADS total score for the full-scale ranges from 0-150 and is calculated by assigning scores to the response categories [0=Very untrue, 1=Untrue, 2=Somewhat untrue, 3=Neutral, 4=Somewhat true, 5=True, 6=True] and summing the score for each of the items. Although most of the evidence comes from high income countries, several BA trials with adolescents have found an association between BADS and treatment effect (Chu et al., 2016; McCauley et al., 2016; Takagaki et al., 2016). Participants will be asked to complete the full-scale (25-items) at baseline (week 0) and end of intervention (week 11), the activation and social impairment subscales at mid-intervention (week 5), and the activation subscale at follow-up (week 24).

Cognition outcomes

Computerised cognitive tasks include computerised paradigms of affective control, social cognition, risk-taking and executive function. Participants will be asked to complete these tasks at baseline (week 0) and end of intervention (week 11).

Affective Set-Shifting: set-shifting was assessed with a modified version of the Wisconsin Card Sorting Task. In this version participants assign cards to one of four decks based on three sorting criteria: (i) the number of items on the card, (ii) the colour of the card, (iii) or the shape of the items (neutral version) or the emotional expression of the faces (affective version). Participants are not told the sorting rule but instead rely on the computerised feedback they receive to determine the correct rule. The sorting rules change suddenly, and participants need to adapt their sorting criterion (Schweizer et al., 2019).

Affective Backwards Digit Span: the backward digit span is an updating task in which participants are asked to recall digits that are serially presented, superimposed over neutral (e.g., scrambled face) or negative (e.g., sad face) images, in reverse order. The task assesses how many digits participants can remember in sequence and was adapted for computerised presentation (Jahanshahi et al., 2008).

Balloon Analogue Risk Task (BART): in this incentivised task, participants earn monetary rewards for inflating a balloon and choose when to stop and claim their reward. With each pump, the potential payoff increases, however if the balloon explodes no reward is given. This task assesses risk-seeking preferences under uncertainty (White et al., 2008).

Emotion recognition task: participants are shown images of faces showing different types of emotional expressions and are asked to classify the emotion that is expressed. They can choose

For peer review only - http://bmjopen.bmj.com/site/about/guidelines.xhtml

one out of four answer options: happy, sad, angry, neutral. Facial expressions are displayed at different intensities to vary task difficulty. Binary variable equal to 1 if facial expression was labelled correctly (Fuhrmann et al., 2016).

Matrix Reasoning Item Bank (MaRs-IB): participants complete a measure of abstract reasoning, where they must select one of several potential objects to complete the pattern of a matrix. The task requires participants to identify the missing element that completes a pattern and is frequently used to test fluid intelligence (Chierchia et al., 2019).

Risky behaviour and peer pressure outcomes

Participants will be asked to complete the risky behaviour outcomes at baseline (week 0) and end of intervention (week 11) using Audio Computer-Assisted Self-Interviewing (ACASI) software. We will measure 4 types of risky behaviours, and construct the following variables:

Risky sexual behaviour: we will create variables for the following measures: (1) *Coital debut before age of 15:* Binary variable equal to 1 if had sex before age 15; (2) *Pregnancy:* Binary variable equal to 1 if ever pregnant/gotten a woman pregnant; Discrete variable equal to the number of times pregnant/gotten a woman pregnant; Binary variable equal to 1 if unintended pregnancy; (3) *Unsafe sex:* Binary variable equal to 1 if unsafe sex; (4) *Multiple partners:* Binary variable equal to 1 if multiple partners in the past 12 months; (5) *Risky sex:* Binary variable equal to 1 if sex while high on alcohol/drugs; (6) *Transactional sex:* Binary variable equal to 1 if had sex in exchange for money/gifts

Substance use: the frequency of substance use will be assessed using the Alcohol, Smoking and Substance Involvement Screening Test for Youth (ASSIST-Y). The ASSIST-Y consists of six questions. For each substance listed, sum the scores for questions 2–6. More information about scoring and cut-offs can be found in (Humeniuk et al., 2016).

Delinquency: Binary variable equal to 1 if, in the past 30 days, the participant has hit, slapped, or physically hurt someone; or broken into a house, school, shop or other building without permission; or caused serious damage to property that did not belong to them, or stolen something.

Gambling: We will create (1) a binary variable equal to 1 if participant won/lost money from gambling or betting in the past 30 days; and (2) a continuous variable equal to the amount won/lost from gambling or betting

Peer pressure: We will create several categorical variables indicating how much pressure does the participant get from his/her friends to: (1) have sex; (2) smoke; (3) drink alcohol; (4) take drugs, where 0=no pressure, to 3=a lot of pressure.

Socioeconomic outcomes

We will measure economic preferences (time preference, risk preference, and loss aversion) using three incentivised tasks (Andersen et al., 2006; Kahneman and Tversky, 2018). In addition, we

For peer review only - http://bmjopen.bmj.com/site/about/guidelines.xhtml
For peer review only

will measure a range of socioeconomic outcomes, including measures of time use, human capital investment, and spending. Participants will be asked to complete these outcomes at baseline (week 0) and end of intervention (week 11). The description of these measures as well as details of this analysis will be documented in a separate analysis plan.

Covariates

Participants and participants' caregivers (if adolescent<18) were asked to complete a brief sociodemographic questionnaire about the household at baseline (week 0). We will also ask participants about trauma (also at baseline only). We will construct the following variables:

Assets. We will measure household assets using two measures, both of which will be assessed only at baseline. Both measures will be used as a control and for heterogeneity analysis: (1) The *Simple Poverty Scorecard Poverty-Assessment Tool for South Africa* (Schreiner, 2017). The questionnaire consists of 12 items. All points in the scorecard are non-negative integers, and total scores range from 0 (most likely below a poverty line) to 100 (least likely below a poverty line); and (2) *Large assets:* Participant's caregivers will be asked whether the household owns different items (e.g., house, motorcycle, jewellery, land, large or small livestock, poultry). We will sum the total number of items the household owns.

Food security. We assessed food insecurity using the six-item Short Form of the Household Food Security Scale (Economic Research Service USDA, 2012). Total score ranges from 0 (=high or marginal food security), to 6 (=very low food security).

Cost for children. Total spent on school-related expenses, clothes, and health care for all children in the household.

Transfers. Total household received in pensions, insurances, scholarships, government grants or lottery in the past 30 days.

Demographic measures: In addition to these household characteristics, we also collected the following demographic indicators: Age; Marital status; Country of origin; Total number of children born alive; Orphan status (2=double orphan, 1=single orphan, 0=not an orphan); Caregiver is not the parent; Living arrangements (binary variable equal to 1 if participant lives in the household without parents; and Education (Grade enrolled in; Highest education level; Missed school days: binary variable equal to 1 if participant missed more than one whole week of school at any one time during the past 12 months).

Trauma. Four brief questions about trauma were included as part of the survey. These questions were initially developed for use in a study of digitally delivered Cognitive Behavioural Therapy, supported by peer mentors, at the University of California Los Angeles (UCLA). The questions ask whether the participant has experienced physical, sexual, or emotional violence currently or in the past. We will construct a binary variable equal to 1 if the participant experienced any type of trauma.

For peer review only - http://bmjopen.bmj.com/site/about/guidelines.xhtml

6. General issues for statistical analysis

6.1 Post randomisation exclusions

The numbers (with percentages of the randomised population) of post-randomisation exclusions will be reported by trial arm and reasons summarised. The following participants will be excluded from the baseline table and the analysis of all outcomes:

- Adolescents who withdrew consent to use their data
- Adolescents for whom an entire record of fraudulent data was detected.

6.2 Population definitions

The analysis will be on the intention to treat (ITT) population; participants will be categorised in the arm they were randomised to, despite the allocation received, excluding the post-randomisation exclusions listed in section 6.1.

- ***Descriptive analysis population:*** Baseline demographic and clinical characteristics will be reported for all participants randomised minus post-randomisation exclusions (Table 2A).
- ***Comparative analysis population*****:** Participants' outcomes will be reported for all participants randomised minus post-randomisation exclusions (Table 2B-2E).
- ***Safety population:*** Safety data will be reported for all participants randomised (Table 2F).

6.3 Missing data

Patterns in missing data will be explored for all analyses. Complete case analyses will be reported in all instances, but in the event of data which appear to be missing at random, we will also consider the use of imputation methods as appropriate. Multiple imputation by chained equations (MICE) is a multiple imputation method used to replace missing data values in a data set under certain assumptions about the data missingness. MICE operates under the assumption that given the variables used in the imputation procedure, the missing data are Missing At Random (MAR), which means that the probability that a value is missing depends only on observed values and not on unobserved values (Schafer and Graham, 2002).

7. Descriptive Analyses

7.1 Baseline characteristics of randomised groups

The following participant and household characteristics at trial entry will be described separately for the two randomised groups:

- *Individual characteristics:* age, sex, marital status, orphan status, number of children, highest educational level.
- *Household characteristics:* household asset index, large assets module, and food security
- *Outcomes at baseline:* mental health outcomes (PHQ-A score, GAD-7 score, CD-RISC score, RRS score, WEMWBS score, BADS score), cognition outcomes (all 5 tasks), risky behaviours and peer pressure, economic preferences, socioeconomic outcomes (employment, earnings, savings, consumption, aspirations).

For peer review only - http://bmjopen.bmj.com/site/about/guidelines.xhtml

Numbers (with percentages) for binary and categorical variables and means (and standard deviations), or medians (with lower and upper quartiles) for continuous variables will be presented.

7.2 Attrition

The number and percentage of losses to follow up among participants will be reported for the two trial arms, and the reasons will be recorded. All deaths of the participants will be reported separately.

We will compare the attrition rate by assigned treatment status to test whether the probability of attrition differs by treatment type. We will analyse:

$$Y_i = Treatment * \beta_1 + \boldsymbol{X_i \lambda} + \boldsymbol{\varepsilon_1}$$

'i' indexes individuals, Y_i is an indicator variable for whether the participant attrited from the round, $Treatment_I$ is an indicator variable for a person assigned to the treatment and X_i is a vector of prespecified covariates, including individual and household characteristics (age, sex, marital status, highest educational level, household asset index, and food security) and mental health outcomes at baseline. We will display this analysis for each round of data collection.

8. Comparative Analyses

Quantitative data will be analysed using Stata Version 17.0 and/or other appropriate statistical analysis packages under the direction of the trial statistician (StataCorp, 2021).

Participants will be analysed in the groups to which they are randomly assigned, regardless of deviation from the protocol or treatment received (ITT population) and the data analyst will be blind to arm allocation. We will use descriptives to explore patterns in the data followed by inferential statistics involving univariate and multivariable models. A two-tailed p-value <0.05 will be considered as statistically significant in the inferential analysis.

8.1 Primary analysis

(1) Feasibility and acceptability of the intervention. Our first co-primary objective (feasibility and acceptability) will be assessed using a mixed-methods approach. We will compute appropriate summary statistics (e.g., proportions, means, SDs) for each quantitative outcome and evaluate the feasibility of the trial based on the pre-defined progression criteria discussed earlier.

The number and percentage of participants screened, assessed for eligibility, randomised in the two arms, and retention rates (including completion rates) will be provided, as well as the number and reason for participants excluded from the study. This will be illustrated with a flowchart, summarising the flow of participants from screening and recruitment to the end of intervention assessment (11 week). See Figure 1.

Treatment adherence rates, acceptability, and fidelity of delivery of the intervention will be reported as shown in Table 2B-2D.

Qualitative interviews will be audio-recorded, transcribed verbatim and translated into English. Transcripts will be analysed using thematic analysis. We will follow Braun and Clarke's six phases of analysis (i.e. becoming familiar with the data, generating initial codes, searching for themes, reviewing themes, defining and naming themes, and producing the report) (Braun and Clarke,

For peer review only - http://bmjopen.bmj.com/site/about/guidelines.xhtml

2006). Coding will be done by two independent researchers. NVivo10, a computer program that aids in the sorting and management of qualitative data, will be used to facilitate the analysis.

(2) Initial efficacy on depressive symptoms

Our second co-primary objective (initial efficacy on depressive symptoms) will be based on the PHQ-A score (continuous) at 11 weeks. Outcomes will be compared between intervention and control groups using a multiple linear regression model and adjusting for covariates.
The primary inference will be estimated as per the following form:

$$\text{PHQscore}_i = \alpha + \beta\text{Treated}_i + \delta^T X_i + \varepsilon_i$$

where 'i' stands for individuals, PHQscore_i denotes the PHQ-A score at the end-of-intervention visit (week 11), Treated_i is an indicator variable equal to one for individuals assigned to receive the treatment and zero if the individual is assigned to the control group, and X_i is a vector of covariates that might affect depressive symptoms based on past literature and data collected from a previous study among adolescents in the study setting (Pettifor et al., 2016). These covariates will include: PHQ score measured at baseline, sex, age, and household asset index
We will also conduct the analysis only including PHQ-A score at baseline and with no further adjustements.

We will test the hypothesis that assignment to the treatment group has no effect on the outcome relative to the control group (β_1=0). We will use robust standard errors to allow for the presence of heteroskedasticity. Results from this analysis will be reported as per Table 2E.

As a secondary analysis, we will make use of the repeated measurements of the PHQ-A throughout the trial (up to 6 times per individual) to evaluate the treatment effects across time. We will rely on statistically advanced methods to compute this (mixed-effect model for repeated measures) and adjust for baseline scores. We will use generalized estimating equations (GEE) based approach which assumes nonvarying (or average) coefficient in the presence of clustering multivariable models with PHQ-A over time as the main outcome. Additional to these we will also run multilevel (hierarchical) models focus on estimating the aspects of the model that vary by group. We will explore various covariance structures forms to model the PHQs over time such as first order auto-regressive, structured and unstructured.

Further exploratory analysis might include choosing additional covariates as part of the primary inference by regressing outcome variables for part of the endline sample on various combinations of baseline covariates and selecting a combination of covariates that explains a large portion of the outcome variation. The choice of covariates is designed to improve the precision of treatment effect estimates by absorbing outcome variation. This exercise will be performed in the control group before endline data is available for the full sample.

For peer review only - http://bmjopen.bmj.com/site/about/guidelines.xhtml

Trial progression criteria

Progression criteria for a definitive RCT will be based on a traffic light system of green (continue to RCT), amber (make modifications in discussion with TSC; proceed with caution), and red (discussion with TSC to not proceed to the RCT) (Avery et al., 2017).

We will evaluate three criteria as follows:

Criteria	Green	Amber	Red
Enrolment (recruitment) rate of eligible participants	≥60%	<60%, ≥40%	<40%
Retention to trial at 11 weeks	≥90%	<90%, ≥50%	<50%
Share of participants that open at least 4 out of 6 of the app episodes	≥70%	<70%, ≥50%	<50%
Share of participants that have 3 out of 6 phone calls with the peer mentor	≥70%	<70%, ≥50%	<50%

8.2 Secondary analysis

As part of the secondary analyses, we will pilot locally adapted outcome measures of mental health, cognitive tasks, risky behaviours, socioeconomic measures, and collect descriptive data to inform the development of a further larger RCT.

For the cognition tasks, we will calculate overall performance and distributions, the correlation between tasks, and convergent and external validity of risk related-measures. For the mental health outcomes, we will perform standard psychometric tests to examine whether the instruments perform well in the study context and calculate bivariate correlations across variables of interest. Specifically, we will assess the measure's reliability (internal consistency and corrected item-total correlations), validity (construct validity, confirmatory factor analysis, and psychometric invariance), and acceptability (descriptive statistics, endorsement rates, missing data, and response-style bias).

We will also analyse treatment effects, but the analysis is exploratory, to inform future work, rather than confirmatory. We will focus on the direction and magnitude of effects. We will not correct p-values for multiple hypothesis testing as drawing conclusions about the statistical significance of effects is less of a focus of this analysis. We anticipate that some secondary analysis will be included in companion papers rather than the main paper.

8.3 Exploring possible mechanisms

Exploratory analyses will be conducted to identify mediators and moderators of the primary outcome at the end-of-intervention visit (week 11). Potential mediators will include executive function and social cognition, as well as resilience, rumination, and functioning. We will decompose the total treatment effect on the primary outcome into (i) an indirect effect through one or more mediators, and (ii) the direct effect of the intervention not captured by these mediator(s) following Acharya et al. (2016). Moderators will include age, education, household asset index, suicide risk, and severity of depressive symptoms at screening.

For peer review only - http://bmjopen.bmj.com/site/about/guidelines.xhtml

8.4 Sensitivity analysis

A multiple imputation analysis will be performed for the primary outcome if attrition exceeds 5%. The multiple imputation model will include baseline characteristics and outcome measures collected prior to the missing assessment, which are associated with missing status (Enders and London, 2010; Xu et al., 2020).

We might also consider running the primary analysis using the per-protocol population, which consists of the ITT population but excludes any participants defined as having a major protocol deviation (see section 10.1).

9. Safety data analysis

Serious adverse events (SAEs) and whether they were 'related' to research procedures or 'unexpected' will be listed by trial arm. As the trial intervention involves increased assessments and contact, the number of safety events detected and reported is likely to be higher in the intervention arm.

10. Protocol non-compliances

A protocol non-compliance is defined as a failure to adhere to the protocol such as the wrong intervention being administered, incorrect data being collected and documented, errors in applying inclusion/exclusion criteria or missed follow-up visits due to error. The overall number of participants experiencing a protocol non-compliance and the total number of non-compliances will be reported. Treatment group will be cross tabulated with type of major and minor deviation.

10.1 Major

A major protocol non-compliance is any failure to comply with the final study protocol as approved by ethics committees, resulting from error, fraud or misconduct and results in the exclusion of a patient from the study. Fraudulent data will be defined as a major protocol non-compliance in this pilot trial.

10.2 Minor

The following will be defined as minor protocol non-compliances:

- *Participants randomised in error.* These include adolescents: (1) Whose informed consent is not fully documented; (2) Aged below 15 or above 19 years; (3) Whose PHQ-A score <5 points at screening; (4) Whose PHQ-A score >19 at screening; (5) With severe psychiatric illness, or a life-threatening or other serious physical illness; (6) With suicidal ideation/ thoughts with specific plans and means identified; (7) Who are planning to move away from the study area in the next 12 weeks; (8) Who do not speak and read sufficiently in Xitsonga
- *Participants who do not receive the allocated intervention*. These include adolescents: (1) In the intervention arm who did not receive the intervention as planned in the protocol; (2) In the control group who received some or all the intervention.
- *The assessor becomes unblinded prior to or during an assessment.*

For peer review only - http://bmjopen.bmj.com/site/about/guidelines.xhtml

11. References

Acharya, A., Blackwell, M., Sen, M., 2016. Explaining Causal Findings Without Bias: Detecting and Assessing Direct Effects. Am. Polit. Sci. Rev. 110, 512–529. https://doi.org/10.1017/S0003055416000216

Andersen, S., Harrison, G.W., Lau, M.I., Rutström, E.E., 2006. Elicitation using multiple price list formats. Exp. Econ. 9, 383–405. https://doi.org/10.1007/s10683-006-7055-6

Arjadi, R., Nauta, M.H., Scholte, W.F., Hollon, S.D., Chowdhary, N., Suryani, A.O., Uiterwaal, C.S.P.M., Bockting, C.L.H., 2018. Internet-based behavioural activation with lay counsellor support versus online minimal psychoeducation without support for treatment of depression: a randomised controlled trial in Indonesia. The Lancet Psychiatry 5, 707–716. https://doi.org/10.1016/S2215-0366(18)30223-2

Asante, K.O., Meyer-Weitz, A., 2014. Measuring resilience among homeless youth: psychometric assessment of the Connor-Davidson Resilience Scale in Ghana. J. Psychol. Africa 24, 321–326. https://doi.org/10.1080/14330237.2014.980620

Avery, K.N.L., Williamson, P.R., Gamble, C., Francischetto, E.O.C., Metcalfe, C., Davidson, P., Williams, H., Blazeby, J.M., Blencowe, N., Bugge, C., Campbell, M., Collinson, M., Cooper, C., Darbyshire, J., Dimairo, M., Doré, C., Eldridge, S., Farrin, A., Foster, N., Gilbody, S., Goodacre, S., Hampson, L., Kolias, A.G., Lamb, S., Lane, A., Maguire, L., Norrie, J., Pickering, R., Shorter, G., Treweek, S., 2017. Informing efficient randomised controlled trials: exploration of challenges in developing progression criteria for internal pilot studies. BMJ Open 7, e013537. https://doi.org/10.1136/BMJOPEN-2016-013537

Bhana, A., Rathod, S.D., Selohilwe, O., Kathree, T., Petersen, I., 2015. The validity of the Patient Health Questionnaire for screening depression in chronic care patients in primary health care in South Africa. BMC Psychiatry 15, 118. https://doi.org/10.1186/s12888-015-0503-0

Braun, V., Clarke, V., 2006. Using thematic analysis in psychology. Qual. Res. Psychol. 3, 77–101. https://doi.org/10.1191/1478088706qp063oa

Chan, A.W., Tetzlaff, J.M., Altman, D.G., Laupacis, A., Gøtzsche, P.C., Krleža-Jerić, K., Hróbjartsson, A., Mann, H., Dickersin, K., Berlin, J.A., Doré, C.J., Parulekar, W.R., Summerskill, W.S.M., Groves, T., Schulz, K.F., Sox, H.C., Rockhold, F.W., Rennie, D., Moher, D., 2013. SPIRIT 2013 statement: Defining standard protocol items for clinical trials. Ann. Intern. Med. 158, 200–207. https://doi.org/10.7326/0003-4819-158-3-201302050-00583

Chibanda, D., Verhey, R., Gibson, L.J., Munetsi, E., Machando, D., Rusakaniko, S., Munjoma, R., Araya, R., Weiss, H.A., Abas, M., 2016. Validation of screening tools for depression and anxiety disorders in a primary care population with high HIV prevalence in Zimbabwe. J. Affect. Disord. 198, 50–55. https://doi.org/10.1016/j.jad.2016.03.006

Chierchia, G., Fuhrmann, D., Knoll, L.J., Pi-Sunyer, B.P., Sakhardande, A.L., Blakemore, S.-J., 2019. The matrix reasoning item bank (MaRs-IB): novel, open-access abstract reasoning items for adolescents and adults. R. Soc. Open Sci. 6, 190232. https://doi.org/10.1098/rsos.190232

Cholera, R., Gaynes, B.N., Pence, B.W., Bassett, J., Qangule, N., Macphail, C., Bernhardt, S., Pettifor, A., Miller, W.C., 2014. Validity of the patient health questionnaire-9 to screen for depression in a high-HIV burden primary healthcare clinic in Johannesburg, South Africa. J. Affect. Disord. 167, 160–166. https://doi.org/10.1016/j.jad.2014.06.003

For peer review only - http://bmjopen.bmj.com/site/about/guidelines.xhtml

Chu, B.C., Crocco, S.T., Esseling, P., Areizaga, M.J., Lindner, A.M., Skriner, L.C., 2016. Transdiagnostic group behavioral activation and exposure therapy for youth anxiety and depression: Initial randomized controlled trial. Behav. Res. Ther. 76, 65–75. https://doi.org/10.1016/j.brat.2015.11.005
Economic Research Service USDA, 2012. Food Security Survey Module: Six-Item Short Form.
Enders, C.K., London, N.Y., 2010. Applied missing data analysis.
Faul, F., Erdfelder, E., Lang, A.G., Buchner, A., 2007. G*Power 3: A flexible statistical power analysis program for the social, behavioral, and biomedical sciences. Behav. Res. Methods 2007 392 39, 175–191. https://doi.org/10.3758/BF03193146
Fu, Z., Burger, H., Arjadi, R., Bockting, C.L.H., 2020. Effectiveness of digital psychological interventions for mental health problems in low-income and middle-income countries: a systematic review and meta-analysis. The Lancet Psychiatry 7, 851–864. https://doi.org/10.1016/S2215-0366(20)30256-X
Fuhrmann, D., Knoll, L.J., Sakhardande, A.L., Speekenbrink, M., Kadosh, K.C., Blakemore, S.-J., 2016. Perception and recognition of faces in adolescence. Sci. Rep. 6, 33497. https://doi.org/10.1038/srep33497
Goin, D.E., Pearson, R.M., Craske, M.G., Stein, A., Pettifor, A., Lippman, S.A., Kahn, K., Neilands, T.B., Hamilton, E.L., Selin, A., MacPhail, C., Wagner, R.G., Gomez-Olive, F.X., Twine, R., Hughes, J.P., Agyei, Y., Laeyendecker, O., Tollman, S., Ahern, J., 2019. Depression and incident HIV in adolescent girls and young women in HPTN 068: Targets for prevention and mediating factors. Am. J. Epidemiol. https://doi.org/10.1093/aje/kwz238
Humeniuk, R., Holmwood, C., Beshara, M., Kambala, A., 2016. ASSIST-Y V1.0: First-Stage Development of the WHO Alcohol, Smoking and Substance Involvement Screening Test (ASSIST) and Linked Brief Intervention for Young People. J. Child Adolesc. Subst. Abuse 25, 384–390. https://doi.org/10.1080/1067828X.2015.1049395
Jacobson, N.S., Martell, C.R., Dimidjian, S., 2006. Behavioral Activation Treatment for Depression: Returning to Contextual Roots. Clin. Psychol. Sci. Pract. 8, 255–270. https://doi.org/10.1093/clipsy.8.3.255
Jahanshahi, M., Saleem, T., Ho, A.K., Fuller, R., Dirnberger, G., 2008. A preliminary investigation of the running digit span as a test of working memory. Behav. Neurol. 20, 17–25. https://doi.org/10.3233/BEN-2008-0212
Jørgensen, I.E., Seedat, S., 2008. Factor structure of the Connor-Davidson Resilience Scale in South African adolescents. Int. J. Adolesc. Med. Health 20, 23–32. https://doi.org/10.1515/IJAMH.2008.20.1.23
Kahn, K., Collinson, M.A., Gómez-Olivé, F.X., Mokoena, O., Twine, R., Mee, P., Afolabi, S.A., Clark, B.D., Kabudula, C.W., Khosa, A., Khoza, S., Shabangu, M.G., Silaule, B., Tibane, J.B., Wagner, R.G., Garenne, M.L., Clark, S.J., Tollman, S.M., 2012. Profile: Agincourt health and socio-demographic surveillance system. Int. J. Epidemiol. 41, 988–1001. https://doi.org/10.1093/ije/dys115
Kahneman, D., Tversky, A., 2018. Prospect theory: An analysis of decision under risk, in: Experiments in Environmental Economics. Taylor and Francis Inc., pp. 143–172. https://doi.org/10.2307/1914185
Kessler, R.C., Berglund, P., Demler, O., Jin, R., Merikangas, K.R., Walters, E.E., 2005. Lifetime prevalence and age-of-onset distributions of DSM-IV disorders in the national comorbidity survey replication. Arch. Gen. Psychiatry. https://doi.org/10.1001/archpsyc.62.6.593
Lehtimaki, S., Martic, J., Wahl, B., Foster, K.T., Schwalbe, N., 2021. Evidence on Digital

For peer review only - http://bmjopen.bmj.com/site/about/guidelines.xhtml

Mental Health Interventions for Adolescents and Young People: Systematic Overview. JMIR Ment Heal. 8, e25847. https://doi.org/10.2196/25847

McCauley, E., Gudmundsen, G., Schloredt, K., Martell, C., Rhew, I., Hubley, S., Dimidjian, S., 2016. The Adolescent Behavioral Activation Program: Adapting Behavioral Activation as a Treatment for Depression in Adolescence. J. Clin. Child Adolesc. Psychol. 45, 291–304. https://doi.org/10.1080/15374416.2014.979933

Olaseni, J.T., 2018. Rumination and Academic Hardiness as Predicators of Suicidal Ideation among Nigerian Adolescents. Abnorm. Behav. Psychol. 04. https://doi.org/10.4172/2472-0496.1000133

Osborn, T.L., Venturo-Conerly, K.E., Wasil, A.R., Schleider, J.L., Weisz, J.R., 2019. Depression and Anxiety Symptoms, Social Support, and Demographic Factors Among Kenyan High School Students. J. Child Fam. Stud. 1–12. https://doi.org/10.1007/s10826-019-01646-8

Pettifor, A., MacPhail, C., Selin, A., Gómez-Olivé, F.X., Rosenberg, M., Wagner, R.G., Mabuza, W., Hughes, J.P., Suchindran, C., Piwowar-Manning, E., Wang, J., Twine, R., Daniel, T., Andrew, P., Laeyendecker, O., Agyei, Y., Tollman, S., Kahn, K., The HPTN 068 protocol team, 2016. HPTN 068: A Randomized Control Trial of a Conditional Cash Transfer to Reduce HIV Infection in Young Women in South Africa—Study Design and Baseline Results. AIDS Behav. 20, 1863–1882. https://doi.org/10.1007/S10461-015-1270-0/FIGURES/3

Schafer, J.L., Graham, J.W., 2002. Missing data: Our view of the state of the art. Psychol. Methods 7, 147–177. https://doi.org/10.1037/1082-989X.7.2.147

Schreiner, M., 2017. Simple Poverty Scorecard Poverty-Assessment Tool South Africa.

Schweizer, S., Parker, J., Leung, J.T., Griffin, C., Blakemore, S.-J., 2019. Age-related differences in affective control and its association with mental health difficulties. Dev. Psychopathol. 1–13. https://doi.org/10.1017/S0954579419000099

StataCorp, 2021. Stata Statistical Software: Release 17.

Takagaki, K., Okamoto, Yasumasa, Jinnin, R., Mori, A., Nishiyama, Y., Yamamura, T., Yokoyama, S., Shiota, S., Okamoto, Yuri, Miyake, Y., Ogata, A., Kunisato, Y., Shimoda, H., Kawakami, N., Furukawa, T.A., Yamawaki, S., 2016. Behavioral activation for late adolescents with subthreshold depression: a randomized controlled trial. Eur. Child Adolesc. Psychiatry 25, 1171–1182. https://doi.org/10.1007/s00787-016-0842-5

Thapar, A., Collishaw, S., Pine, D.S., Thapar, A.K., 2012. Depression in adolescence. Lancet. https://doi.org/10.1016/S0140-6736(11)60871-4

Weiner, B.J., Lewis, C.C., Stanick, C., Powell, B.J., Dorsey, C.N., Clary, A.S., Boynton, M.H., Halko, H., 2017. Psychometric assessment of three newly developed implementation outcome measures. Implement. Sci. 12, 1–12. https://doi.org/10.1186/S13012-017-0635-3/TABLES/3

White, T.L., Lejuez, C.W., de Wit, H., 2008. Test-Retest Characteristics of the Balloon Analogue Risk Task (BART). Exp. Clin. Psychopharmacol. 16, 565–570. https://doi.org/10.1037/a0014083

Xu, X., Xia, L., Zhang, Q., Wu, S., Wu, M., Liu, H., 2020. The ability of different imputation methods for missing values in mental measurement questionnaires. BMC Med. Res. Methodol. 20, 1–9. https://doi.org/10.1186/S12874-020-00932-0/TABLES/3

For peer review only - http://bmjopen.bmj.com/site/about/guidelines.xhtml

12. Table and Figures

Table 1. DoBAt's time schedule of enrolment, interventions, assessments, and visits for participants

	STUDY PERIOD								
Assessment	Enrolment	Allocation	Post-allocation						Close-out
**TIME POINT (in weeks)**	$-t_1$	0	1	2.5	5	7.5	11	24 WKS
**ENROLMENT**								
Eligibility screen (school survey)	X							
Informed consent	X							
Allocation		X						
Phone delivery		X						
**INTERVENTION**								
Intervention arm: Kuamsha app & Peer Mentor calls			●	—	—	—	●	
Control arm: Kuchunguza app			●	—	—	—	●	
**ASSESSMENTS**								
School survey: PHQ-A, sociodemographic questions & eligibility checklist	X							
Baseline assessment: demographic questionnaire, GAD-7, CD-RISC, RRS, WEMWBS, BADS, trauma, five social-affective and cognition tasks, risky behaviours, and socioeconomic outcomes	X							
Symptom monitoring: PHQ-A				X		X		
Mid-intervention assessment: PHQ-A, GAD-7, RRS, WEMWBS short form, BADS (activation & social impairment subscales)					X			
Post-intervention assessment: same as baseline + PHQA+ acceptability questionnaire & IDIs with subsample							X	
Follow-up assessment: PHQ-A, GAD-7, RRS, BADS activation subscale, WEMWBS short form								X

Table 2. Dummy tables

**TABLE 2A. Baseline comparability of randomised groups**		
	**Treatment group** (N=XX)	**Control group** (N=XX)
**Individual characteristics**<br>• Age<br>• Sex<br>• Marital status<br>• Orphan status<br>• Number of children<br>• Highest educational level<br>• Employment status		
**Household characteristics**<br>• Household asset index<br>• Food security<br>• Maternal/paternal education level		
**Outcomes at baseline**<br>• PHQ-A score<br>• GAD-7 score<br>• CD-RISC score<br>• RRS score<br>• WEMWBS score<br>• BADS score<br>• Affective Set-Shifting		

review only

For peer review only - http://bmjopen.bmj.com/site/about/guidelines.xhtml

• Affective Backwards Digit Span<br>• Balloon Analogue Risk Task (BART)<br>• Emotion recognition task<br>• Matrix Reasoning Item Bank (MaRs-IB)<br>• Risky behaviours<br>• Peer pressure<br>• Economic preferences<br>• Employment<br>• Earnings<br>• Savings<br>• Consumption<br>• Aspirations		

Comparative analysis:

TABLE 2B. FEASIBILITY	
	**Treatment group *(Kuamsha app)*** (N=XX)
**Number of participants with data**	
**Recruitment and retention:**<br>• Enrolment rate of eligible participants<br>• Retention at the end of the intervention (11 weeks)	
**Treatment adherence rate:**<br>• N (%) opened ≥4 modules/episodes in the app<br>• N (%) had ≥3 phone calls with the peer mentors (excluding introductory call)	
**Kuamsha app engagement metrics**<br>• Number of logins<br>• Number of episodes opened<br>• Number of episodes completed<br>• Total time spent on app<br>• Number of set up weekly activities<br>• Number of completed weekly activities	
	**Control group *(Kuchunguza app)*** (N=XX)
**Number of participants with data**	
**Kuchunguza app engagement metrics**<br>• Number of videos opened<br>• Number of videos completed<br>• Total time spent on app	

TABLE 2C. ACCEPTABILITY		
	**Treatment group** (N=XX)	**Control group** (N=XX)

For peer review only - http://bmjopen.bmj.com/site/about/guidelines.xhtml

Acceptability of using the app<br>• AIM score<br>• IAM score<br>• FIM score	Median {IQR} [Range]	Median {IQR} [Range]
Acceptability of the peer mentor programme (only treatment group)<br>• AIM score<br>• IAM score<br>• FIM score	Median {IQR} [Range]	N. A

TABLE 2D. FIDELITY OF DELIVERY OF THE INTERVENTION	
	**Treatment group** (N of peer mentors=XX)
**Adherence and competence of peer mentors:**<br>• N (%) sessions that meet ≥90% criteria for adherence<br>• Competency Assessment score	

TABLE 2E. INITIAL EFFICACY			
	**Treatment group** (N=XX)	**Control group** (N=XX)	**Mean difference (95% CI)**
Primary inference:<br>PHQ-A score at end of intervention (11 weeks)<br>• Mean (SD)<br>• Unknown			

Safety population:

TABLE 2F. ADVERSE EVENTS						
**AE number**	**Treatment allocation**	**Description**	**Severity**	**Related**	**Action taken**	**Outcome**

For peer review only - http://bmjopen.bmj.com/site/about/guidelines.xhtml

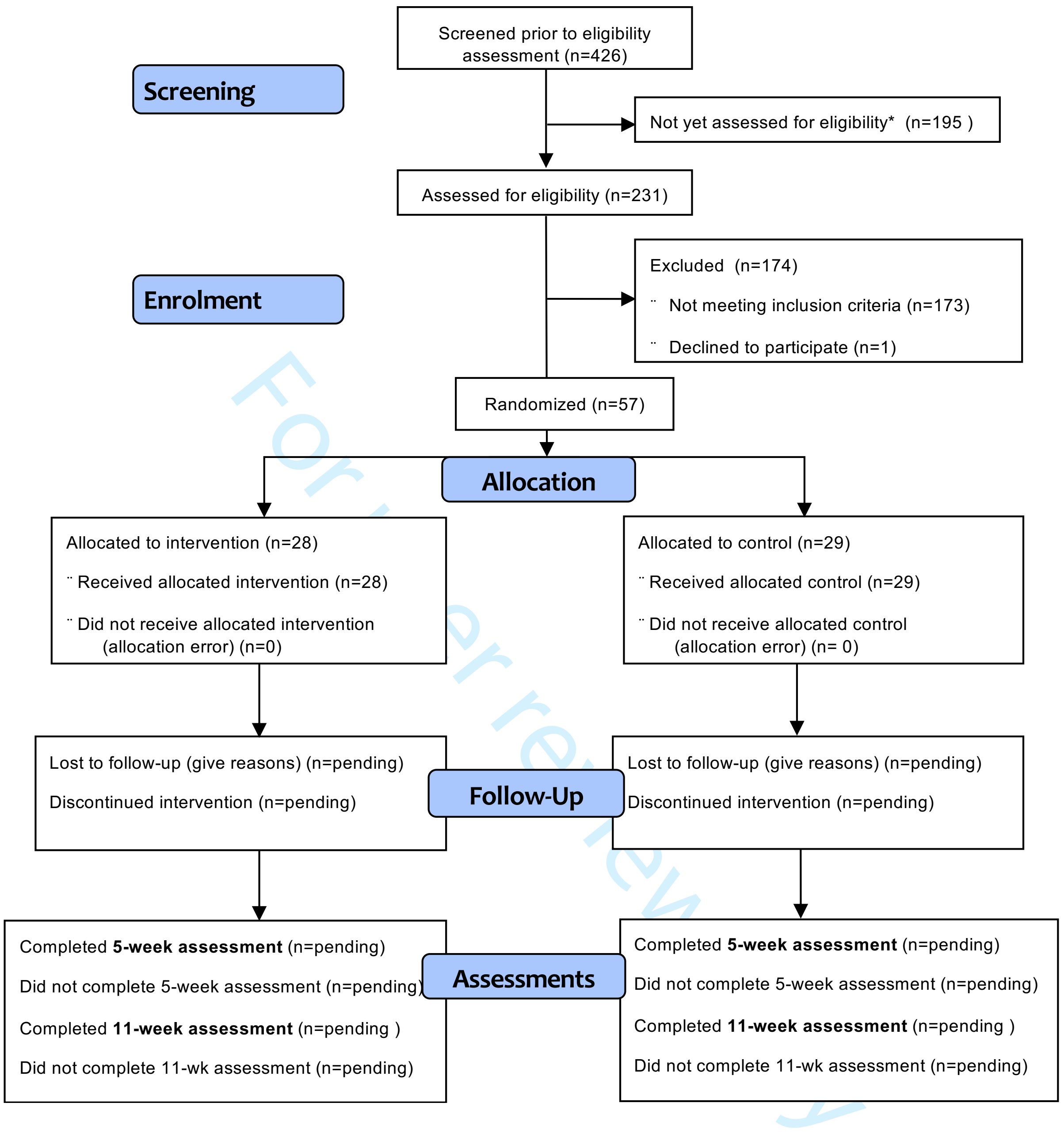

Figure 1. CONSORT flow diagram for DoBAt study

13. Approval

For peer review only

Lead Medical Statistician	Name: Professor Eustasius Musenge	
	Signature	Date: 03/11/22
Chief Co-Investigators	Name: Professor Kathleen Kahn	
	Signature	Date: 03/11/22
	Name: Professor Alan Stein	
	Signature	Date: 03/11/22
Chair of DSMB	Name: Professor Bonga Chiliza	
	Signature	Date 04/11/2022
Chair of Trial Steering Committee	Name: Professor Roz Shafran	
	Signature	Date 04.11.22

SPIRIT
STANDARD PROTOCOL ITEMS: RECOMMENDATIONS FOR INTERVENTIONAL TRIALS

SPIRIT 2013 Checklist: Recommended items to address in a clinical trial protocol and related documents*

Section/item	Item No	Description	Page
**Administrative information**			
Title	1	Descriptive title identifying the study design, population, interventions, and, if applicable, trial acronym	Page 1
Trial registration	2a	Trial identifier and registry name. If not yet registered, name of intended registry	Page 2
	2b	All items from the World Health Organization Trial Registration Data Set	Sup. Material (Table 5)
Protocol version	3	Date and version identifier	Sup. Material (Table 5)
Funding	4	Sources and types of financial, material, and other support	Page 14
Roles and responsibilities	5a	Names, affiliations, and roles of protocol contributors	Pages 1 & 14
	5b	Name and contact information for the trial sponsor	Sup. Material (Table 5)
	5c	Role of study sponsor and funders, if any, in study design; collection, management, analysis, and interpretation of data; writing of the report; and the decision to submit the report for publication, including whether they will have ultimate authority over any of these activities	Page 14
	5d	Composition, roles, and responsibilities of the coordinating centre, steering committee, endpoint adjudication committee, data management team, and other individuals or groups overseeing the trial, if applicable (see Item 21a for data monitoring committee)	Sup. Material (Table 1)

For peer review only - http://bmjopen.bmj.com/site/about/guidelines.xhtml

Introduction			
Background and rationale	6a	Description of research question and justification for undertaking the trial, including summary of relevant studies (published and unpublished) examining benefits and harms for each intervention	Pages 3-4
	6b	Explanation for choice of comparators	Page 6
Objectives	7	Specific objectives or hypotheses	Page 4
Trial design	8	Description of trial design including type of trial (eg, parallel group, crossover, factorial, single group), allocation ratio, and framework (eg, superiority, equivalence, noninferiority, exploratory)	Page 5
**Methods: Participants, interventions, and outcomes**			
Study setting	9	Description of study settings (eg, community clinic, academic hospital) and list of countries where data will be collected. Reference to where list of study sites can be obtained	Page 5
Eligibility criteria	10	Inclusion and exclusion criteria for participants. If applicable, eligibility criteria for study centres and individuals who will perform the interventions (eg, surgeons, psychotherapists)	Pages 5-6
Interventions	11a	Interventions for each group with sufficient detail to allow replication, including how and when they will be administered	Pages 6-7 & Sup. Material (Tables 2-4)
	11b	Criteria for discontinuing or modifying allocated interventions for a given trial participant (eg, drug dose change in response to harms, participant request, or improving/worsening disease)	n/a
	11c	Strategies to improve adherence to intervention protocols, and any procedures for monitoring adherence (eg, drug tablet return, laboratory tests)	Pages 6-8
	11d	Relevant concomitant care and interventions that are permitted or prohibited during the trial	Page 6

For peer review only - http://bmjopen.bmj.com/site/about/guidelines.xhtml

Outcomes	12	Primary, secondary, and other outcomes, including the specific measurement variable (eg, systolic blood pressure), analysis metric (eg, change from baseline, final value, time to event), method of aggregation (eg, median, proportion), and time point for each outcome. Explanation of the clinical relevance of chosen efficacy and harm outcomes is strongly recommended	Pages 7-9
Participant timeline	13	Time schedule of enrolment, interventions (including any run-ins and washouts), assessments, and visits for participants. A schematic diagram is highly recommended (see Figure)	Page 10 (Table 2, Figure 1)
Sample size	14	Estimated number of participants needed to achieve study objectives and how it was determined, including clinical and statistical assumptions supporting any sample size calculations	Page 12
Recruitment	15	Strategies for achieving adequate participant enrolment to reach target sample size	Page 10
**Methods: Assignment of interventions (for controlled trials)**			
Allocation:			
Sequence generation	16a	Method of generating the allocation sequence (eg, computer-generated random numbers), and list of any factors for stratification. To reduce predictability of a random sequence, details of any planned restriction (eg, blocking) should be provided in a separate document that is unavailable to those who enrol participants or assign interventions	Page 11
Allocation concealment mechanism	16b	Mechanism of implementing the allocation sequence (eg, central telephone; sequentially numbered, opaque, sealed envelopes), describing any steps to conceal the sequence until interventions are assigned	Page 11
Implementation	16c	Who will generate the allocation sequence, who will enrol participants, and who will assign participants to interventions	Page 11
Blinding (masking)	17a	Who will be blinded after assignment to interventions (eg, trial participants, care providers, outcome assessors, data analysts), and how	Page 11
	17b	If blinded, circumstances under which unblinding is permissible, and procedure for revealing a participant's allocated intervention during the trial	Page 11
**Methods: Data collection, management, and analysis**			

Data collection methods	18a	Plans for assessment and collection of outcome, baseline, and other trial data, including any related processes to promote data quality (eg, duplicate measurements, training of assessors) and a description of study instruments (eg, questionnaires, laboratory tests) along with their reliability and validity, if known. Reference to where data collection forms can be found, if not in the protocol	Pages 7-11
	18b	Plans to promote participant retention and complete follow-up, including list of any outcome data to be collected for participants who discontinue or deviate from intervention protocols	Sup. Material (SAP)
Data management	19	Plans for data entry, coding, security, and storage, including any related processes to promote data quality (eg, double data entry; range checks for data values). Reference to where details of data management procedures can be found, if not in the protocol	Sup. Material (SAP)
Statistical methods	20a	Statistical methods for analysing primary and secondary outcomes. Reference to where other details of the statistical analysis plan can be found, if not in the protocol	Pages 11-12 & Sup. Material (SAP)
	20b	Methods for any additional analyses (eg, subgroup and adjusted analyses)	Pages 11-12 & Sup. Material (SAP)
	20c	Definition of analysis population relating to protocol non-adherence (eg, as randomised analysis), and any statistical methods to handle missing data (eg, multiple imputation)	Pages 11-12 & Sup. Material (SAP)
**Methods: Monitoring**			
Data monitoring	21a	Composition of data monitoring committee (DMC); summary of its role and reporting structure; statement of whether it is independent from the sponsor and competing interests; and reference to where further details about its charter can be found, if not in the protocol. Alternatively, an explanation of why a DMC is not needed	Sup. Material (Table 1)
	21b	Description of any interim analyses and stopping guidelines, including who will have access to these interim results and make the final decision to terminate the trial	Sup. Material (SAP)

For peer review only - http://bmjopen.bmj.com/site/about/guidelines.xhtml

Harms	22	Plans for collecting, assessing, reporting, and managing solicited and spontaneously reported adverse events and other unintended effects of trial interventions or trial conduct	Page 13
Auditing	23	Frequency and procedures for auditing trial conduct, if any, and whether the process will be independent from investigators and the sponsor	N/A
**Ethics and dissemination**			
Research ethics approval	24	Plans for seeking research ethics committee/institutional review board (REC/IRB) approval	Page 13
Protocol amendments	25	Plans for communicating important protocol modifications (eg, changes to eligibility criteria, outcomes, analyses) to relevant parties (eg, investigators, REC/IRBs, trial participants, trial registries, journals, regulators)	Sup. Material (Table 5)
Consent or assent	26a	Who will obtain informed consent or assent from potential trial participants or authorised surrogates, and how (see Item 32)	Page 13
	26b	Additional consent provisions for collection and use of participant data and biological specimens in ancillary studies, if applicable	N/A
Confidentiality	27	How personal information about potential and enrolled participants will be collected, shared, and maintained in order to protect confidentiality before, during, and after the trial	Page 13
Declaration of interests	28	Financial and other competing interests for principal investigators for the overall trial and each study site	Page 14
Access to data	29	Statement of who will have access to the final trial dataset, and disclosure of contractual agreements that limit such access for investigators	Sup. Material (SAP)
Ancillary and post-trial care	30	Provisions, if any, for ancillary and post-trial care, and for compensation to those who suffer harm from trial participation	N/A
Dissemination policy	31a	Plans for investigators and sponsor to communicate trial results to participants, healthcare professionals, the public, and other relevant groups (eg, via publication, reporting in results databases, or other data sharing arrangements), including any publication restrictions	Page 14
	31b	Authorship eligibility guidelines and any intended use of professional writers	Page 14

For peer review only - http://bmjopen.bmj.com/site/about/guidelines.xhtml

	31c	Plans, if any, for granting public access to the full protocol, participant-level dataset, and statistical code	Sup. Material (Table 5)
Appendices			
Informed consent materials	32	Model consent form and other related documentation given to participants and authorised surrogates	Sup. Material
Biological specimens	33	Plans for collection, laboratory evaluation, and storage of biological specimens for genetic or molecular analysis in the current trial and for future use in ancillary studies, if applicable	N/A

*It is strongly recommended that this checklist be read in conjunction with the SPIRIT 2013 Explanation & Elaboration for important clarification on the items. Amendments to the protocol should be tracked and dated. The SPIRIT checklist is copyrighted by the SPIRIT Group under the Creative Commons "Attribution-NonCommercial-NoDerivs 3.0 Unported" license.

For peer review only

BMJ Open

Digital delivery of Behavioural Activation therapy to overcome depression and facilitate social and economic transitions of adolescents in South Africa (the DoBAt study): protocol for a pilot randomised controlled trial

Journal:	*BMJ Open*
Manuscript ID	bmjopen-2022-065977.R2
Article Type:	Protocol
Date Submitted by the Author:	02-Dec-2022

Complete List of Authors:	Moffett, Bianca; University of the Witwatersrand, MRC/Wits Rural Public Health and Health Transitions Research Unit (Agincourt), School of Public Health, Faculty of Health Sciences<br>Pozuelo, Julia; University of Oxford, Psychiatry; University of the Witwatersrand, MRC/Wits Rural Public Health and Health Transitions Research Unit (Agincourt), School of Public Health, Faculty of Health Sciences<br>van Heerden , Alastair ; Human Sciences Research Council, Center for Community Based Research<br>O'Mahen, Heather; University of Exeter, Mood Disorders Centre, Department of Psychology<br>Craske, Michelle; University of California Los Angeles, Psychology, Psychiatry and Biobehavioral Sciences<br>Sodi, Tholene; University of Limpopo, Psychology<br>Lund, Crick; University of Cape Town, Alan J Flisher Centre for Public Mental Health, Department of Psychiatry and Mental Health; King's College London, Centre for Global Mental Health, Institute of Psychiatry, Psychology and Neuroscience<br>Orkin, Kate; University of Oxford, Blavatnik School of Government<br>Kilford, Emma J; University College London, Department of Clinical, Educational and Health Psychology<br>Blakemore, Sarah-Jayne; University of Cambridge, Department of Psychology; University of the Witwatersrand, MRC/Wits Rural Public Health and Health Transitions Research Unit (Agincourt), School of Public Health, Faculty of Health Sciences<br>Mahmud, Mahreen; University of Exeter, Department of Economics<br>Musenge, Eustasius; University of the Witwatersrand, MRC/Wits Rural Public Health and Health Transitions Research Unit (Agincourt), School of Public Health, Faculty of Health Sciences; University of the Witwatersrand, Division of Epidemiology and Biostatistics, School of Public Health, Faculty of Health Sciences<br>Davis, Meghan; University of Oxford, Department of Psychiatry<br>Makhanya, Zamakhanya; University of the Witwatersrand, MRC/Wits Rural Public Health and Health Transitions Research Unit (Agincourt), School of Public Health, Faculty of Health Sciences<br>Baloyi, Tlangelani; University of Limpopo, Department of Psychology<br>Mahlangu, Daniel; University of the Witwatersrand, MRC/Wits Rural Public Health and Health Transitions Research Unit (Agincourt), School of

	Public Health, Faculty of Health Sciences<br>Chierchia, Gabriele; University of Cambridge, Department of Psychology<br>Fielmann, Sophie L.; University of Cambridge, Department of Psychology<br>Gómez-Olivé, F. Xavier; University of the Witwatersrand, MRC/Wits Rural Public Health and Health Transitions Research Unit (Agincourt), School of Public Health, Faculty of Health Sciences<br>Valodia, Imraan; University of the Witwatersrand, Southern Centre for Inequality Studies<br>Tollman, Stephen; University of the Witwatersrand, MRC/Wits Rural Public Health and Health Transitions Research Unit (Agincourt), School of Public Health, Faculty of Health Sciences; Umeå Universitet, Umeå Centre for Global Health Research, Division of Epidemiology and Global Health, Department of Public Health and Clinical Medicine<br>Kahn, Kathleen; University of the Witwatersrand, MRC/Wits Rural Public Health and Health Transitions Research Unit (Agincourt), School of Public Health, Faculty of Health Sciences; Umeå Universitet, Umeå Centre for Global Health Research, Division of Epidemiology and Global Health, Department of Public Health and Clinical Medicine<br>Stein, Alan; University of Oxford, Department of Psychiatry; University of the Witwatersrand, MRC/Wits Rural Public Health and Health Transitions Research Unit (Agincourt), School of Public Health, Faculty of Health Sciences
**Primary Subject Heading**:	Mental health
Secondary Subject Heading:	Mental health, Global health, Public health
Keywords:	Depression & mood disorders < PSYCHIATRY, Child & adolescent psychiatry < PSYCHIATRY, PUBLIC HEALTH

SCHOLARONE™
Manuscripts

Digital delivery of Behavioural Activation therapy to overcome depression and facilitate social and economic transitions of adolescents in South Africa (the DoBAt study): protocol for a pilot randomised controlled trial

Authors

Bianca D. Moffett*[1], Julia R. Pozuelo*[1,2], Alastair van Heerden[3], Heather A. O'Mahen[4], Michelle Craske[5], Tholene Sodi[6], Crick Lund[7,8], Kate Orkin[9], Emma J Kilford[10], Sarah-Jayne Blakemore[1,11], Mahreen Mahmud[12], Eustasius Musenge[1,16], Meghan Davis[2], Zamakhanya Makhanya[1], Tlangelani Baloyi[6], Daniel Mahlangu[1] Gabriele Chierchia[11], Sophie L. Fielmann[11], Xavier Gomez-Olive[1], Imraan Valodia[13], Stephen Tollman[1,14], Kathleen Kahn**[1,14], Alan Stein**[1,2,15]

* denotes joint first author
** denotes joint senior author

Affiliations

[1] MRC/Wits Rural Public Health and Health Transitions Research Unit (Agincourt), School of Public Health, Faculty of Health Sciences, University of the Witwatersrand, Johannesburg, South Africa
[2] Department of Psychiatry, University of Oxford, Oxford, United Kingdom
[3] Center for Community Based Research, Human Sciences Research Council, Pietermaritzburg, South Africa
[4] Mood Disorders Centre, Department of Psychology, University of Exeter, Exeter, United Kingdom
[5] Department of Psychology, Psychiatry and Biobehavioral Sciences at the University of California, Los Angeles, United States of America
[6]Department of Psychology, University of Limpopo, Polokwane, South Africa
[7]Alan J Flisher Centre for Public Mental Health, Department of Psychiatry and Mental Health, University of Cape Town, Cape Town, South Africa
[8] Centre for Global Mental Health, Institute of Psychiatry, Psychology and Neuroscience, King's College London, London, United Kingdom
[9] Blavatnik School of Government, University of Oxford, Oxford, United Kingdom
[10] Department of Clinical, Educational and Health Psychology, University College London, London, United Kingdom
[11] Department of Psychology, University of Cambridge, Cambridge, United Kingdom
[12]Department of Economics, University of Exeter, Exeter, United Kingdom
[13]Southern Centre for Inequality Studies, University of the Witwatersrand, Johannesburg, South Africa
[14] Umeå Centre for Global Health Research, Division of Epidemiology and Global Health, Department of Public Health and Clinical Medicine, Umeå University, Umeå, Sweden
[15]Africa Health Research Institute, KwaZulu Natal, South Africa
[16]Division of Epidemiology and Biostatistics, School of Public Health, Faculty of Health Sciences, University of the Witwatersrand, Johannesburg, South Africa

Corresponding author: Bianca D Moffett, MRC/Wits Rural Public Health and Health Transitions Research Unit (Agincourt), School of Public Health, Faculty of Health Sciences, University of the Witwatersrand, Johannesburg, South Africa, bianca.moffett@wits.ac.za

Word Count
Abstract: 298
Text: 4665(Excl. References, Tables and Figures)
Tables & Figures: 2 Tables, 1 Figure
References: 67

Keywords: depression, adolescents, pilot trial, feasibility, digital, app, Behavioural Activation

For peer review only

ABSTRACT

Introduction: Scalable psychological treatments to address depression among adolescents are urgently needed. This is particularly relevant to low- and middle-income countries where 90% of the world's adolescents live. Whilst digital delivery of Behavioural Activation presents a promising solution, its feasibility, acceptability, and effectiveness among adolescents in an African context remains to be shown.

Methods and analysis: This study is a two-arm single-blind individual-level randomised controlled pilot trial to assess the feasibility, acceptability, and initial efficacy of digitally delivered Behavioural Activation (BA) therapy amongst adolescents with depression. The intervention has been co-produced with adolescents at the study site. The study is based in the rural northeast of South Africa in the Bushbuckridge sub-district of Mpumalanga Province. A total of 200 adolescents with symptoms of mild to moderately-severe depression on the Patient Health Questionnaire Adolescent Version (PHQ-A) will be recruited (1:1 allocation ratio). The treatment group will receive Behavioural Activation therapy via a smartphone application (the Kuamsha app) supported by trained peer mentors. The control group will receive an Enhanced Standard of Care. The feasibility and acceptability of the intervention will be evaluated using a mixed-methods design, and signals of the initial efficacy of the intervention in reducing symptoms of depression will be determined on an intention-to-treat basis. Secondary objectives are to pilot a range of cognitive, mental health, risky behaviour, and socioeconomic measures; and to collect descriptive data on the feasibility of trial procedures to inform the development of a further larger trial.

Ethics and dissemination: This study has been approved by the University of the Witwatersrand Human Research Ethics Committee (MED20-05-011) and the Oxford Tropical Research Ethics Committee (OxTREC 34-20). Study findings will be published in scientific open access peer-reviewed journals, presented at scientific conferences, and communicated to participants, their caregivers, public sector officials, and other relevant stakeholders.

Trial registration: This trial was registered on 19 November 2020 with the South African National Clinical Trials Registry (DOH-27-112020-5741) and the Pan African Clinical Trials Registry (PACTR202206574814636).

STRENGTHS AND LIMITATIONS

- The intervention has been iteratively co-produced with local adolescents using multiple user-centred design methods to ensure that it was engaging, culturally relevant, and usable for the targeted population.
- We will use a rigorous mixed-methods design to assess the feasibility, acceptability, and initial efficacy of the digital intervention.
- Culturally adapted measures of social-affective cognition, as well as a range of relevant mental health, risky behaviours, and socioeconomic measures, will be piloted.
- We have excluded participants with severe depression and high risk suicidal ideation.
- This study uses the PHQ-A, a validated screening tool to screen for depression, but does not provide a clinical diagnosis of depression.

For peer review only - http://bmjopen.bmj.com/site/about/guidelines.xhtml

1. INTRODUCTION

Despite growing acknowledgement of the importance of adolescent mental health and the potential of investing in this formative period, it remains severely neglected[1]. This is particularly true in low and middle-income countries (LMICs), where 90% of the world's adolescents live, socioeconomic adversities affecting mental health are prevalent, and mental health resources remain poor[2]. Given that most mental disorders have their onset before 25 years of age, early identification and treatment of emergent mental disorders has the potential to reduce chronicity and sequelae for individuals, and be an efficient strategy for addressing population-level mental health[3]. Investments in mental health have the potential to support socioeconomic transitions and contribute to breaking the cycle of poverty and mental ill-health in LMICs[4].

Adolescence is a critical developmental period during which individuals develop their self-identity, acquire skills and preferences with which they navigate future challenges, and make decisions that can affect their long-term health, education, relationships, and employment prospects[5,6]. Higher-order cognitive functions such as executive function and social cognition, which regulate the ability to strategise, set and maintain goals, and build successful relationships with others, develop significantly during this period[7]. Thus, the physical, cognitive, social, and emotional capabilities acquired during adolescence lay a foundation for the wellbeing of individuals throughout their adult lives[8]. Depression interferes with the acquisition of these capacities, thus limiting young people's ability to fulfil their potential.

Globally, depression is one of the leading causes of disability amongst adolescents, and suicide is the third leading cause of death amongst the 15-19 year age group[2,9]. Left untreated, depression affects interpersonal relationships, interferes with schooling, and disrupts productivity[10]. It also has a marked negative effect on executive function and social cognition, with depressed individuals consistently underperforming on cognitive assessments compared to healthy controls[11,12]. Furthermore, depression in adolescence has been associated with a greater risk for substance use, poor sexual health, delinquency, and a significant reduction in future income[13,14]. Scalable psychological therapies to address depression amongst adolescents, particularly in low-resource contexts, are urgently needed.

Several barriers hinder progress in providing treatment to adolescents with depression in LMICs. Firstly, whilst various treatments for depression have been shown to be effective amongst adolescents in high-income contexts, few have been adapted for or rigorously evaluated in low-resource or culturally diverse settings[15,16]. Secondly, there is a major shortage of mental health professionals in most LMICs[17], and although task-sharing approaches present a promising strategy, they require substantial inputs in training and supervision of non-specialist healthcare workers[18]. Thirdly, there are significant challenges associated with relying on an already overburdened healthcare sector, not least that depression is rarely detected in primary care in most LMICs[19]. Finally, even in contexts where adolescent mental health services are available, stigma, lack of mental health awareness, and issues with the acceptability of services prevent adolescents from seeking care[20].

For peer review only - http://bmjopen.bmj.com/site/about/guidelines.xhtml

Digitally delivered Behavioural Activation (BA) therapy offers the potential to overcome a number of these barriers. BA is a highly effective psychological therapy that is easier to deliver and less costly than Cognitive Behavioural Therapy (CBT)[21,22]. Importantly, there is also evidence that it can be effectively adapted for use: 1) with adolescents[23,24], 2) in low-resource contexts and diverse cultural settings[25–27] and 3) in a digital format[28]. Delivering BA in a digital format may be particularly helpful in contexts where there is a paucity of mental health specialists and health systems are already juggling multiple competing priorities. It also offers the potential to overcome internalised barriers to care, such as stigma and lack of mental health awareness, and this form of guided self-help may be more acceptable to adolescents[20].

Despite the many potential advantages of digital mental health interventions, studies that have evaluated their effectiveness amongst adolescents have yielded mixed results [29,30]. Most of the evidence on the effectiveness of digitally-delivered BA comes from studies conducted in high-income countries[28], and it is, therefore, an open question whether it is possible to generalise these findings to an African context, where conditions and resources differ vastly [31,32]. Furthermore, studies of online self-help interventions amongst adults in LMICs showed high attrition rates[31], and adherence to mental health apps in all contexts is often low[33]. While some commercial smartphone apps attract more users, many have not been rigorously evaluated and show little fidelity to evidence-based treatments [34,35].

This study will address an important gap in the literature by providing evidence on the feasibility, acceptability, and initial efficacy of using digitally delivered Behavioural Activation to address depression amongst adolescents in an LMIC context. Furthermore, we will pilot a range of relevant mental health, risky behaviour and socioeconomic measures, as well as novel culturally adapted measures of executive function and social cognition.

Study objectives

The co-primary objectives of this pilot study are: 1) to determine the feasibility and acceptability of a digitally delivered Behavioural Activation therapy intervention for adolescents living with depression in the Bushbuckridge sub-district of Mpumalanga province, South Africa; and 2) to provide preliminary evidence on any signals of initial efficacy (direction and magnitude) of any effects of the intervention on depressive symptoms amongst adolescents in the intervention arm compared to control.

The secondary objectives are: 1) to pilot a range of mental health, social-affective cognition, risky behaviours, and socioeconomic measures; and 2) to collect descriptive data on trial procedures such as recruitment, retention, data collection, randomisation and blinding to inform key parameters in the development of a further large-scale trial.

2. METHODS AND ANALYSIS

This protocol is reported in accordance with the Standard Protocol Items: Recommendations for Intervention Trials (SPIRIT) 2013 statement[36]. The statistical aspects of the pilot study are summarised here with details fully described in a Statistical Analysis Plan (SAP) that will be available before the recruitment of the last participant.

For peer review only - http://bmjopen.bmj.com/site/about/guidelines.xhtml

2.1 Study design

The DoBAt study is a two-arm single-blind individual-level randomised controlled pilot feasibility trial. A total of 200 adolescents will be recruited (1:1 allocation ratio).

2.2 Study Setting

The study will be based in the Bushbuckridge sub-district of Mpumalanga Province, South Africa. Whilst South Africa is classified as a middle-income country, large inequalities exist, and the study setting represents a rural area characterised by socioeconomic disadvantage and high rates of youth unemployment. Since 1992 the MRC/Wits Rural Public Health and Health Transitions Research Unit (MRC/Wits-Agincourt) has collected population data, with vital events (pregnancy outcome, deaths, in- and out-migration) and household composition updated annually through its Health and socio-Demographic Surveillance System (HDSS). The total population under surveillance is currently ~116,000 inhabitants residing in 31 contiguous villages[37]. Recruitment will take place at schools within the study site. Notably, a previous study amongst adolescent females in this setting reported a prevalence of depressive symptoms of 18.2%[38] and yet access to treatment is severely limited. Similar data for males is not available.

2.3 Patient and Public Involvement

Adolescents and members of the public have been involved at several stages of the study. The intervention was developed through extensive formative research and user-centred design with adolescents in the study area. We also conducted participatory workshops with adolescents and met with local educators, healthcare workers, and relevant non-governmental organisations to obtain their input on trial methods, including recruitment and risk management strategies. We will seek the involvement of adolescents and members of the public in developing appropriate methods to disseminate study findings.

2.4 Eligibility criteria

To be eligible for inclusion in the pilot trial participants must: 1) be between 15 and 19 years of age and in grades 9 to 11 at the beginning of the study; 2) have symptoms of mild to moderately-severe depression indicated by a score between 5 and 19 on the Patient Health Questionnaire Adolescent Version (PHQ-A); 3) be able to read sufficiently in the local language (Xitsonga) to use the Kuamsha app; 4) intend to continue living in the study site for 12-weeks after the baseline assessment; and 5) provide written informed assent/consent to participate in the study, as well as parent/guardian consent if younger than 18 years.

Participants will be excluded if they: 1) have symptoms of severe depression as indicated by a score of >19 on the PHQ-A; 2) have current suicidal ideation with specific plans and means identified; 3) are receiving psychological treatment for a mental health condition at the time of enrolment; 4) have been hospitalised for at least five days for a severe psychiatric illness (specifically Bipolar Mood Disorder, Schizophrenia or other Psychotic Disorders) or life-

For peer review only - http://bmjopen.bmj.com/site/about/guidelines.xhtml

threatening or other serious medical illness; 5) have a history of Bipolar Mood Disorder, Schizophrenia or other Psychotic Disorders; 6) lack capacity to consent to treatment or research participation or to use the app. Participants excluded for points 1) and 2) will be assessed by the Risk Management Team and referred to local clinical services as per prior arrangements with local service providers.

2.5 Intervention

All participants in the intervention and control arms will be given the entry-level Samsung Galaxy A2 Core Android smartphone, which they can keep at the end of the study. Furthermore, participants in both groups will receive 200MB of mobile internet data at six different time points (0; 2.5; 5; 7.5; 11 and 24 weeks) to ensure they have data to use the app and complete the online surveys. Participants in both arms will receive active symptom monitoring via text messages sent to the smartphone every 2.5 weeks. Any adolescents who develop severe depression or high-risk suicidal ideation will be assessed by the Risk Management Team (further details provided in Table 1 of the Supplemental Materials) and referred to local clinical services. Adolescents started on anti-depressant medication or who receive psychological therapy because of these referrals will not be discontinued, but we will take note of concomitant care and will examine this using sensitivity analyses.

Control arm: Enhanced Standard of Care

The non-intervention arm will receive a control app (the *Kuchunguza app*) containing six video clips from *WildEarth-SafariLive*, a locally produced wildlife series. Each video clip takes approximately 15-20 minutes to complete and allows users to explore the African wilderness while listening to calming and atmospheric sounds. In the given context, the control represents an enhancement of standard care since most adolescents with depression would not usually receive any intervention or active symptom monitoring and referral.

Intervention arm: Kuamsha programme

The intervention arm will receive six modules of Behavioural Activation (BA) therapy via a smartphone application (the *Kuamsha app*) supported by trained Peer Mentors, implemented over 10 weeks. The app and phone calls from the Peer Mentors together comprise the Kuamsha programme.

The Kuamsha app is primarily an interactive narrative game consisting of six tailored modules (sessions) containing Behavioural Activation's core principles integrated into the gamified story content format[39]. Each module takes approximately 15-20 mins to complete, and they cover topics such as identifying and engaging in meaningful activities and using strategies to overcome barriers, for example, using problem-solving, effective communication, getting enough sleep, and disengaging from rumination. Each module is followed by a homework activity where the participant is encouraged to reflect on the BA principles outlined in each module and think about ways to apply the principles to their own lives. Participants will be asked to report often they completed the homework activities and their mood as they were doing these activities. The app includes game design elements to stimulate motivation and performance, including character personification, in-app points, and reminders/notifications. Example mock-ups of the Kuamsha app and a summary of each module are shown in Tables 2-4 in the Supplemental Materials.

For peer review only - http://bmjopen.bmj.com/site/about/guidelines.xhtml

The Kuamsha app will be supplemented by weekly phone calls (15 mins per module) from Trained Peer Mentors. There will be seven calls in total, including one introductory phone call and six calls to cover module content. Peer Mentors will attempt to reach participants by calling up to 5 times per week. The role of the Peer Mentors is mainly to support adherence and compliance with the app, troubleshoot problems related to the use of the app, and assist with the implementation of the homework activities. They will be trained not to provide additional advice or counselling and conduct their calls according to a pre-determined checklist of activities to help ensure fidelity. Peer Mentors will be Xitsonga-speaking students or recent graduates from the department of psychology or social work at an accredited South African university. The Trial Psychologist will train and supervise them according to the training manual developed specifically for the intervention.

2.6 Primary objectives and outcome measures

(1) Feasibility and acceptability of the intervention. A mixed-methods approach will be adopted to establish feasibility and acceptability.

- **Feasibility** will be assessed by collecting data on the following:
  (1) Recruitment (enrolment rate of eligible participants) and retention in the trial at the end of the intervention period (11 weeks).
  (2) Feasibility of testing procedures and data collection methods, including assessment of completion rates.
  (3) Treatment adherence rates, where adherence is defined as having opened at least 4 out of 6 of the app modules and as having completed 3 out of 6 phone calls with the peer mentor (excluding the introductory call). We will complement the treatment adherence rates with engagement metrics collected via the app (number of times participants logged into the app, number of modules opened and completed, total time spent on the app, number of weekly activities set to do, and number of times the participant completed the weekly activities).
- **Acceptability** of the intervention and study procedures will be assessed via:
  (1) An acceptability questionnaire conducted at the end of intervention assessment (week 11) with all participants. The questionnaire consists of three measures: Acceptability of Intervention Measure (AIM), Intervention Appropriateness Measure (IAM), and Feasibility of Intervention Measure (FIM)[40]. Each measure consists of 4 items. The total score ranges from 1 to 5 and is calculated by averaging response scores to the response categories. We will calculate an average score for each of the measures. We will inquire about the acceptability of the app using these three measures (to both the intervention and control groups) and the acceptability of the peer mentor programme (to the intervention group only).
  (2) In-depth interviews with a subsample of participants. We estimate that interviews with 20 participants will provide a sufficient range of experiences and perspectives to reach data saturation. This subsample will contain participants from the treatment and control arms and will be stratified based on high versus low app engagement.
- ***Fidelity of delivery of the intervention*** will be assessed by collecting data on adherence and competence of Trained Peer Mentors.

For peer review only - http://bmjopen.bmj.com/site/about/guidelines.xhtml

(1) Adherence of the peer mentors is defined as the number of sessions that meet at least 90% of the criteria for adherence according to the training protocol. Independent raters will listen to a random sample (10%) of recordings of Peer Mentors' phone calls with participants and assess them against the training protocol.
(2) Competence of the peer mentors will be expressed as a percentage based on their Competency Assessment Test. This test will include a written test and observation of skills through role-playing to assess Knowledge, Attitudes and Practices. Tests will be scored by the Trial Psychologist using a pre-determined scoring system.

Trial progression criteria

Feasibility of the intervention and trial procedures and progression criteria for a definitive RCT are given in Table 1. These will be based on a traffic light system of green (continue to RCT), amber (make modifications to trial procedures before embarking on a definitive RCT), and red (a definitive RCT is unlikely to be feasible)[41].

Table 1. Trial progression criteria

**Criteria**	**Green**	**Amber**	**Red**
Enrolment (recruitment) rate of eligible participants	≥60%	<60%, ≥40%	<40%
Retention in the trial at 11 weeks	≥90%	<90%, ≥50%	<50%
Proportion of participants that open at least 4 out of 6 of the app modules	≥70%	<70%, ≥50%	<50%
Proportion of participants that have 3 out of 6 phone calls with the peer mentor	≥70%	<70%, ≥50%	<50%

(2) Signals of Initial Efficacy on depressive symptoms.
This will be assessed using the PHQ-A score at the end of intervention assessment (week 11). The PHQ-A is a widely used and well-established measure of adolescent depressive symptoms over the past two weeks[42]. The PHQ-A will be administered in Xitsonga. Whilst this measure has not been validated in Xitsonga, it showed good psychometric properties with a sample of adolescents and young adults in South Africa and Kenya [43–45] and has been validated in various other South African languages which also form part of the 'Nguni' language group, including isiXhosa [46], seTswana [43], isiZulu and seSotho[44]. The questionnaire consists of 9 items. The PHQ-A total score ranges from 0 to 27 and is calculated by assigning scores to the response categories [0=not at all, 1=several days, 2=more than half the days, 3-nearly every day] and summing the score for each of the items. Higher scores indicate greater severity of depression. PHQ-A scores of 5, 10, 15, and 20 represent mild, moderate, moderately severe and severe depression, respectively [42]. We will use this scale to assess depressive symptoms at screening, week 5, week 11, and week 24. Participants will also be asked to complete this scale

For peer review only - http://bmjopen.bmj.com/site/about/guidelines.xhtml

at week 2.5 and week 7.5 as part of the symptom monitoring. More information on how we will analyse this data and trial progression criteria can be found in the data analysis section.

2.7 Secondary objectives and outcome measures

As part of our secondary objectives, we will pilot locally adapted measures of mental health, social-affective and cognitive processing, risky behaviours, and socioeconomic outcomes. The overall aim of implementing these measures is to assess their acceptability, feasibility, and variation, and use these descriptive data to inform the development of a future larger trial. More details on how we will analyse these secondary outcomes can be found in the Statistical Analysis Plan in the Supplemental Materials.

(i) *Mental health outcomes:* include the following scales Generalised Anxiety Disorder (GAD-7)[47]; Connor-Davidson Resilience Scale (CD-RISC) [48]; Brief Rumination Response Scale (RRS) [49]; Warwick-Edinburgh Mental Wellbeing Scale (WEMWBS)[50]; Behavioural Activation for Depression Scale (BADS)[51]. Participants will be asked to complete these scales at baseline (week 0), mid-intervention (week 5), end of intervention (week 11), and follow-up (week 24). See Table 2 for details.

(ii) Social-affective and cognitive processing: include five computerised cognitive tasks measuring affective set-shifting, affective working memory, risk-seeking preferences, emotion recognition, and abstract reasoning. The tasks include a modified version of the Wisconsin Card Sorting Task[52]; the Backward Digit Span Task[53]; the Balloon Analogue Risk Task (BART)[54]; an Emotion Recognition Task[55]; and the Matrix Reasoning Item Bank (MaRs-IB) Task[56]. Participants will be asked to complete these tasks at baseline (week 0) and end of intervention (week 11).

(iii) Risky behaviours: The questions on sexual behaviours include a subset of items from the Wits Reproductive Health and HIV Institute (Wits RHI) sexual behaviour survey[57]. We will measure substance use using the Alcohol, Smoking and Substance Involvement Screening Test for Youth (ASSIST-Y)[58]. We will also ask about delinquency, gambling, and peer influence in participants' engagement with risky behaviours. Participants will be asked to complete these tasks at baseline (week 0) and end of intervention (week 11).

(iv) Socioeconomics: We will measure economic preferences (time preference, risk preference, and loss aversion) using three incentivised tasks[59,60]. In addition, we will measure a range of socioeconomic outcomes, including measures of time use, human capital investment, and spending. Participants will be asked to complete these tasks at baseline (week 0) and end of intervention (week 11).

2.8 Recruitment and participant timeline

Participants will be recruited through a two-stage recruitment process. The first stage of recruitment (Phase 1) will consist of a screening survey conducted in schools to identify adolescents with symptoms of mild to moderately-severe depression. The second stage of recruitment (Phase 2) will be done with adolescents who score between 5 and 19 on the PHQ-A in the school survey and who also meet the other eligibility criteria.

For peer review only - http://bmjopen.bmj.com/site/about/guidelines.xhtml

The pilot trial consists of an 11-week intervention treatment phase, and participants will be followed up for a further 13 weeks after completing the intervention (i.e., a total of 24 weeks). Primary and secondary outcomes will be assessed at the end of intervention assessment (week 11). We will use different methods to assess these outcomes. Specifically, we will ask about mental health (including PHQ-A) and risky behaviours using Audio Computer-Assisted Self-Interviewing (ACASI) software. Under this approach, participants will listen to pre-recorded survey questions through headphones and select their responses on a tablet computer. The behavioural tasks will be performed individually by participants on a tablet computer, in a quiet location under the instruction of a trained fieldworker. All app engagement metrics (including treatment adherence) will be captured on an online database automatically as participants engage with the app. The acceptability questionnaire will be sent via text message to the study phones. The socioeconomic outcomes will be assessed by blinded outcomes assessors. Table 2 indicates the time schedule of enrolment, interventions and assessments for participants, and Figure 1 shows the flow of participants.

Table 2. Schedule of enrolment, interventions, and assessments for participants

	STUDY PERIOD							
Assessment	Enrolment	Allocation	Post-allocation					Close-out
TIME POINT (in weeks)	$-t_1$	0	1	2.5	5	7.5	11	24
ENROLMENT								
Eligibility screen (school survey)	X							
Informed consent	X							
Allocation		X						
Phone delivery		X						
INTERVENTION								
Intervention arm: Kuamsha app & Peer Mentor calls			●	—	—	—	●	
Control arm: Kuchunguza app			●	—	—	—	●	
ASSESSMENTS								
School survey: PHQ-A, sociodemographic questions & eligibility checklist	X							
Baseline assessment: demographic questionnaire, GAD-7, CD-RISC, RRS, WEMWBS, BADS, five social-affective and cognition tasks, risky behaviours, and socioeconomic outcomes	X							
Symptom monitoring: PHQ-A				X		X		
Mid-intervention assessment: PHQ-A, GAD-7, RRS, WEMWBS short form, BADS (activation & social impairment subscales)					X			

For peer review only - http://bmjopen.bmj.com/site/about/guidelines.xhtml

End of intervention assessment: same as baseline + PHQ-A + acceptability questionnaire & in-depth interviews with subsample							X	
Follow-up assessment: PHQ-A + GAD-7, RRS, BADS (activation subscale), WEMWBS short form								X

Note: BADS: Behavioural Activation for Depression Scale; CD-RISC: Connor-Davidson Resilience Scale; GAD-7: Generalised Anxiety Disorder; PHQ-A: Patient Health Questionnaire Adolescent Version; RRS: Brief Rumination Response Scale; WEMWBS: Warwick-Edinburgh Mental Wellbeing Scale.

2.9 Allocation and blinding

Participants will be randomly assigned to the intervention or control arm with a 1:1 allocation using a computerised minimisation algorithm, balanced by sex (male or female) and severity of depressive symptoms (<10 or ≥ 10 on the PHQ-A). The minimisation algorithm was generated by the Centre for Healthcare Randomised Trials (CHaRT) at the University of Aberdeen. Participants will be allocated using CHaRT's online software and the Trial Manager will oversee the enrolment of participants as per assignment.

For practical and risk management reasons the Trial Manager, Trial Psychologist, Peer Mentors, and Fieldworkers delivering the phones to participants and receiving calls to troubleshoot problems with the app/ phone (i.e., "Phones Team") will not be blinded. However, all fieldworkers conducting outcomes assessments (i.e., the "Assessments Team") will be blinded to participants' allocation status. Any breaches in blinding will be documented, and we will ensure to change the fieldworker conducting subsequent assessments on a particular adolescent where unblinding has occurred.

2.10 Analysis and statistical methods

Quantitative data will be analysed using *Stata* Version 14.0[61], R[62] and other appropriate statistical analysis packages under the direction of the trial statistician.

Participants will be analysed in the groups to which they are randomly assigned, regardless of deviation from the protocol or treatment received (intention to treat population), and the data analyst will be blind to arm allocation. We will use descriptives to explore patterns in the data, followed by inferential statistics involving univariate and multivariable models. A two-tailed p-value <0.05 will be considered statistically significant in the inferential analyses.

Primary objectives

Our first co-primary objective (feasibility and acceptability) will be assessed using a mixed-methods approach. We will compute appropriate summary statistics (e.g., proportions, means, SDs, etc.) for each quantitative outcome and evaluate the feasibility of the trial based on the pre-defined progression criteria discussed earlier.

For peer review only

For peer review only - http://bmjopen.bmj.com/site/about/guidelines.xhtml

Qualitative interviews will be audio-recorded, transcribed verbatim and translated into English. Transcripts will be analysed using thematic analysis. We will follow Braun and Clarke's six phases of analysis (i.e. becoming familiar with the data, generating initial codes, searching for themes, reviewing themes, defining and naming themes, and producing the report)[63]. Coding will be done by two independent researchers. NVivo10, a computer program that aids in the sorting and management of qualitative data, will be used to facilitate the analysis.

Our second co-primary objective (initial efficacy on depressive symptoms) will be based on the PHQ-A score (continuous) at 11 weeks. Outcomes will be compared between intervention and control groups using a linear regression model and adjusting for covariates (PHQ score measured at baseline, sex, age, depression severity, and household asset index). As a secondary analysis, we will make use of the repeated measurements of the PHQ-A throughout the trial (up to 6 times per individual) to evaluate the treatment effects over time. The details of this analysis will be documented in the SAP.

Secondary objectives

The descriptive data of the secondary outcomes will include possible floor and ceiling effects, accuracy, duration, latency, and efficiency. We will perform standard psychometric tests to examine whether the instruments perform well in the study context (e.g., reliability, validity, acceptability) and calculate bivariate correlations across variables of interest. We anticipate that some secondary analyses will be included in companion papers rather than the main paper. The details of this analysis will be documented in a separate analysis plan.

2.11 Sample size considerations

The sample size was calculated on the basis of one of our co-primary objectives which aims to provide preliminary evidence of signals of efficacy (direction and magnitude) of any effects of the intervention on depressive symptoms (measured by the PHQ-A) amongst adolescents in the intervention arm compared to the control group.

Statistical power was calculated to detect differences between two independent groups, in a two-sided test with an α of 0.05 and a power of $1-\beta=0.80$, for an effect size of 0.45. This effect size is based on findings from previous studies investigating digital psychological interventions, with effect size (Cohen's d) ranging from 0.24 to 0.57[29,64,65]. Given these findings, we chose a small-to-medium effect size of 0.45 to account for the limited number of studies conducted with our target population and in our study setting. We allowed for 25% attrition, based on a previous study with adolescents in the Agincourt setting[66]. Given these assumptions, we aimed to recruit 200 participants at baseline. We calculated the required sample size using G*Power software, version 3.1.9.3[67].

2.12 Trial status

Enrolment of the first participant occurred on the 25th of November 2021. The trial is currently ongoing, and we expect to finish data collection in January 2023.

For peer review only - http://bmjopen.bmj.com/site/about/guidelines.xhtml

3. ETHICS AND DISSEMINATION

3.1 Research Ethics Approval

Ethical approval for this study has been obtained from the University of the Witwatersrand Human Research Ethics Committee (MED20-05-011), Ehlanzeni District and Mpumalanga Provincial Departments of Health and Education, and the Oxford Tropical Research Ethics Committee (OxTREC 34-20). This trial was registered with the South African National Clinical Trials Registry (DOH-27-112020-5741) and the Pan African Clinical Trials Registry (PACTR202206574814636) in November 2020. Further details on Trial Registration can be found in Table 5 of the Supplemental Material.

3.2 Informed Consent

At both stages of recruitment, we will obtain informed consent from participants aged 18 or over and informed assent and parental/guardian consent from participants younger than 18. All information sheets will be available in Xitsonga and English, and assent/consent will be obtained by trained and supervised bilingual fieldworkers.

3.3 Confidentiality and management of participant data

The confidentiality of participants' data and information will be respected and maintained by all study staff. Staff members will be trained accordingly and required to sign a non-disclosure agreement. A unique participant identification number will be used to link study data and information. All electronic data will be stored in a secure, protected and access-controlled database at the Agincourt data centre. Paper-based documentation will be stored in a locked cabinet and only accessible to authorised staff. Confidentiality may be broken in instances of immediate harm to self or others, as detailed in consent and assent forms.

3.4 Harms/ Anticipated risks

A comprehensive Risk Management Protocol has been developed to ensure the safety of participants in the trial. Participants with severe depression, suicidal ideation and other risks will receive a telephonic and/or in-person assessment by a member of the Risk Management Team. Subsequently, appropriate referrals and linkage to care will be made using established networks with local providers. Details of the independent Data and Safety Monitoring Board (DSMB) and Trial Steering Committee (TSC) are provided in Table 1 of the Supplemental Materials. Serious Adverse Events will be reported to the DSMB chair within 48 hours and to both Ethics Committees within seven days. Summary tables of all Adverse Events will be sent to the DSMB on a quarterly basis and to the Ethics Committees on an annual basis.

3.5 Publication and dissemination

Results of the trial will be communicated to participants, the public, researchers, healthcare professionals, and policymakers. We will seek the involvement of adolescents and members of the public in the development of appropriate methods to disseminate study findings. Policymakers and other key stakeholders within provincial and national departments of health, education, and social development, as well as relevant local NGOs, will be engaged in dialogue and supplied with a technical brief to convey the results of the trial and their implications for policy and practice. Trial results will be published as soon as possible after completion, and ensuing publications will be made open access. Results will also be presented at relevant national

For peer review only - http://bmjopen.bmj.com/site/about/guidelines.xhtml

and international conferences. Authorship will be determined in accordance with the ICMJE guidelines, and other contributors will be acknowledged.

Acknowledgements
We would like to acknowledge the Field Supervisors Meriam Meritze, Princess Makhubela and Nokthula Mayindi, as well as all the Fieldworkers, Registered Counsellor, Tinyiko Mafumo, and Peer Mentors for their hard work and dedication. We would also like to thank all the adolescents who were involved in the formative work and participatory workshops that were integral to the design and development of the intervention, as well as the educators, healthcare workers and non-governmental organisations that provided valuable input into the trial design, particularly regarding recruitment and risk management strategies.

Funding Statement
Research reported in this publication was supported by the South African Medical Research Council with funds received from the South African National Department of Health and the UK Medical Research Council, with funds received from the UK Government's Newton Fund (grant number MR/S008748/1). It also received financial support from the NIHR Oxford Health Biomedical Research Centre (grant number: N/A). The study was conducted within the MRC/Wits Rural Public Health and Health Transitions Research Unit and Agincourt Health and socio-Demographic Surveillance System, a node of the South African Population Research Infrastructure Network (SAPRIN), supported by the Department of Science and Innovation, the University of the Witwatersrand, and the Medical Research Council, South Africa (grant number: N/A). SJB is funded by Wellcome (grant number WT107496/Z/15/Z), the MRC-UK, the Jacobs Foundation, the Wellspring Foundation, and the University of Cambridge (grant number: N/A). JRP is funded by Fundación Rafael del Pino and received funding support from MRC UK and the Wellspring Philanthropic Fund (grant number: N/A). EJK received funding support from a UKRI-GCRF Impact Acceleration Award (grant number: N/A). Study funders played no role in the study design, collection, management, analysis or interpretation of data, the writing of this report, or the decision to submit it for publication.

Competing Interests
The authors declare no competing interests.

Authors' contributions
AS, KK, ST, MC, HO, SJB, CL, TS, KO, JRP, EJK, BM, MM, XGO, IV and AvH made substantial contributions to the conception and design of the study. BM, JRP, MD, SLF, GC and DM were responsible for programming study measures and training field teams. ZM and TB trained and supervised peer mentors. JRP, KO and EM drafted the Statistical Analysis Plan. The first draft of the manuscript was written by BM and JRP. All authors reviewed the manuscript, and gave the final approval of the version to be published. BM submitted the manuscript.

For peer review only

For peer review only - http://bmjopen.bmj.com/site/about/guidelines.xhtml

REFERENCES

1 Kieling C, Baker-Henningham H, Belfer M, *et al.* Child and adolescent mental health worldwide: evidence for action. *Lancet* 2011;**378**:1515–25. doi:10.1016/S0140-6736(11)60827-1

2 Erskine HE, Moffitt TE, Copeland WE, *et al.* A heavy burden on young minds: the global burden of mental and substance use disorders in children and youth. *Psychol Med* 2015;**45**:1551. doi:10.1017/S0033291714002888

3 Herrman H, Patel V, Kieling C, *et al.* Time for united action on depression: a Lancet–World Psychiatric Association Commission. *Lancet* 2022;**399**:957–1022. doi:10.1016/S0140-6736(21)02141-3

4 Lund C, De Silva M, Plagerson S, *et al.* Poverty and mental disorders: breaking the cycle in low-income and middle-income countries. *Lancet* 2011;**378**:1502–14. doi:10.1016/S0140-6736(11)60754-X

5 Sawyer SM, Afifi RA, Bearinger LH, *et al.* Adolescence: A foundation for future health. Lancet. 2012;**379**:1630–40. doi:10.1016/S0140-6736(12)60072-5

6 Almlund M, Duckworth AL, Heckman J, *et al.* Personality Psychology and Economics. In: Hanushek EA, Machin SJ, Woessman L, eds. *Handbook of the Economics of Education*. Elsevier 2011. 1–181. doi:10.1016/B978-0-444-53444-6.00001-8

7 Blakemore SJ, Choudhury S. Development of the adolescent brain: implications for executive function and social cognition. *J Child Psychol Psychiatry* 2006;**47**:296–312. doi:10.1111/J.1469-7610.2006.01611.X

8 Sheehan P, Sweeny K, Rasmussen B, *et al.* Building the foundations for sustainable development: a case for global investment in the capabilities of adolescents. *Lancet* 2017;**390**:1792–806. doi:10.1016/S0140-6736(17)30872-3

9 Kieling C, Adewuya A, Fisher HL, *et al.* Identifying depression early in adolescence. *Lancet Child Adolesc Heal* 2019;**3**:211. doi:10.1016/S2352-4642(19)30059-8

10 Thapar A, Collishaw S, Pine DS, *et al.* Depression in adolescence. Lancet. 2012;**379**:1056–67. doi:10.1016/S0140-6736(11)60871-4

11 Snyder HR. Major depressive disorder is associated with broad impairments on neuropsychological measures of executive function: A meta-analysis and review. *Psychol Bull* 2013;**139**:81–132. doi:10.1037/a0028727

12 Weightman MJ, Air TM, Baune BT. A review of the role of social cognition in major depressive disorder. Front. Psychiatry. 2014;**5**:179. doi:10.3389/fpsyt.2014.00179

13 Pozuelo JR, Desborough L, Stein A, *et al.* Systematic Review and Meta-Analysis: Depressive Symptoms and Risky Behaviours among Adolescents in Low- and Middle-Income Countries. *J Am Acad Child Adolesc Psychiatry* Published Online First: 2021. doi:10.1016/j.jaac.2021.05.005

14 Lund C, Myer L, Stein DJ, *et al.* Mental illness and lost income among adult South Africans. *Soc Psychiatry Psychiatr Epidemiol* 2013;**48**:845–51. doi:10.1007/s00127-012-0587-5

15 Das JK, Salam RA, Lassi ZS, *et al.* Interventions for Adolescent Mental Health: An Overview of Systematic Reviews. *J Adolesc Heal* 2016;**59**:S49. doi:10.1016/J.JADOHEALTH.2016.06.020

16 Klasen H, Crombag AC. What works where? A systematic review of child and adolescent mental health interventions for low and middle income countries. *Soc Psychiatry*

For peer review only - http://bmjopen.bmj.com/site/about/guidelines.xhtml

Psychiatr Epidemiol 2013;**48**:595–611. doi:10.1007/S00127-012-0566-X/TABLES/3
17 Kakuma R, Minas H, Van Ginneken N, *et al.* Human resources for mental health care: current situation and strategies for action. *Lancet* 2011;**378**:1654–63. doi:10.1016/S0140-6736(11)61093-3
18 Kemp CG, Petersen I, Bhana A, *et al.* Supervision of Task-Shared Mental Health Care in Low-Resource Settings: A Commentary on Programmatic Experience. *Glob Heal Sci Pract* 2019;**7**:150–9. doi:10.9745/GHSP-D-18-00337
19 Fekadu A, Demissie M, Birhane R, *et al.* Under detection of depression in primary care settings in low and middle-income countries: a systematic review and meta-analysis. *Syst Rev* 2022;**11**:1–10. doi:10.1186/S13643-022-01893-9/FIGURES/3
20 Stunden C, Zasada J, VanHeerwaarden N, *et al.* Help-Seeking Behaviors of Transition-Aged Youth for Mental Health Concerns: Qualitative Study. *J Med Internet Res* 2020;**22**:e18514. doi:10.2196/18514
21 Richards DA, Ekers D, McMillan D, *et al.* Cost and Outcome of Behavioural Activation versus Cognitive Behavioural Therapy for Depression (COBRA): a randomised, controlled, non-inferiority trial. *Lancet* 2016;**388**:871–80. doi:10.1016/S0140-6736(16)31140-0
22 Ekers D, Webster L, Van Straten A, *et al.* Behavioural activation for depression; An update of meta-analysis of effectiveness and sub group analysis. *PLoS One* 2014;**9**:e100100. doi:10.1371/journal.pone.0100100
23 Tindall L, Mikocka-Walus A, McMillan D, *et al.* Is behavioural activation effective in the treatment of depression in young people? A systematic review and meta-analysis. *Psychol Psychother Theory, Res Pract* 2017;**90**:770–96. doi:10.1111/PAPT.12121
24 Martin F, Oliver T. Behavioral activation for children and adolescents: a systematic review of progress and promise. *Eur Child Adolesc Psychiatry* 2019;**28**:427–41. doi:10.1007/S00787-018-1126-Z/FIGURES/2
25 Jacob M, L. Keeley M, Ritschel L, *et al.* Behavioural activation for the treatment of low-income, African American adolescents with major depressive disorder: a case series. *Clin Psychol Psychother* 2013;**20**:87–96. doi:10.1002/CPP.774
26 Patel V, Weobong B, Weiss HA, *et al.* The Healthy Activity Program (HAP), a lay counsellor-delivered brief psychological treatment for severe depression, in primary care in India: a randomised controlled trial. *Lancet* 2017;**389**:176–85. doi:10.1016/S0140-6736(16)31589-6
27 Lehmann DC, Bördlein C. A Systematic Review of Culturally Adapted Behavioral Activation Treatments for Depression: *Res Soc Work Pract* 2020;**30**:688–702. doi:10.1177/1049731520915635
28 Huguet A, Miller A, Kisely S, *et al.* A systematic review and meta-analysis on the efficacy of Internet-delivered behavioral activation. *J Affect Disord* 2018;**1**:27–38.
29 Lehtimaki S, Martic J, Wahl B, *et al.* Evidence on Digital Mental Health Interventions for Adolescents and Young People: Systematic Overview. *JMIR Ment Heal* 2021;**8**:e25847. doi:10.2196/25847
30 Eilert N, Wogan R, Leen A, *et al.* Internet-Delivered Interventions for Depression and Anxiety Symptoms in Children and Young People: Systematic Review and Meta-analysis. *JMIR Pediatr Parent 2022;5(2)e33551 https//pediatrics.jmir.org/2022/2/e33551* 2022;**5**:e33551. doi:10.2196/33551
31 Naslund JA, Aschbrenner KA, Araya R, *et al.* Digital technology for treating and

For peer review only - http://bmjopen.bmj.com/site/about/guidelines.xhtml

preventing mental disorders in low-income and middle-income countries: a narrative review of the literature. *The Lancet Psychiatry* 2017;**4**:486–500. doi:10.1016/S2215-0366(17)30096-2
32 Lehtimaki S, Martic J, Wahl B, *et al.* Evidence on Digital Mental Health Interventions for Adolescents and Young People: Systematic Overview. *JMIR Ment Heal* 2021;**8**. doi:10.2196/25847
33 Linardon J, Fuller-Tyszkiewicz M. Attrition and adherence in smartphone-delivered interventions for mental health problems: A systematic and meta-analytic review. *J Consult Clin Psychol* 2020;**88**:1–13. doi:10.1037/CCP0000459
34 Schueller SM, Torous J. Scaling evidence-based treatments through digital mental health. *Am Psychol* 2020;**75**:1093–104. doi:10.1037/AMP0000654
35 Huguet A, Rao S, McGrath PJ, *et al.* A Systematic Review of Cognitive Behavioral Therapy and Behavioral Activation Apps for Depression. *PLoS One* 2016;**11**:e0154248. doi:10.1371/JOURNAL.PONE.0154248
36 Chan AW, Tetzlaff JM, Altman DG, *et al.* SPIRIT 2013 statement: Defining standard protocol items for clinical trials. *Ann Intern Med* 2013;**158**:200–7. doi:10.7326/0003-4819-158-3-201302050-00583
37 Kahn K, Collinson MA, Gómez-Olivé FX, *et al.* Profile: Agincourt health and socio-demographic surveillance system. *Int J Epidemiol* 2012;**41**:988–1001. doi:10.1093/ije/dys115
38 Goin DE, Pearson RM, Craske MG, *et al.* Depression and incident HIV in adolescent girls and young women in HPTN 068: Targets for prevention and mediating factors. *Am J Epidemiol* Published Online First: 29 October 2019. doi:10.1093/aje/kwz238
39 Jacobson NS, Martell CR, Dimidjian S. Behavioral Activation Treatment for Depression: Returning to Contextual Roots. *Clin Psychol Sci Pract* 2006;**8**:255–70. doi:10.1093/clipsy.8.3.255
40 Weiner BJ, Lewis CC, Stanick C, *et al.* Psychometric assessment of three newly developed implementation outcome measures. *Implement Sci* 2017;**12**:1–12. doi:10.1186/S13012-017-0635-3/TABLES/3
41 Avery KNL, Williamson PR, Gamble C, *et al.* Informing efficient randomised controlled trials: exploration of challenges in developing progression criteria for internal pilot studies. *BMJ Open* 2017;**7**:e013537. doi:10.1136/BMJOPEN-2016-013537
42 Kroenke K, Spitzer RL, Williams JBW. The PHQ-9: Validity of a brief depression severity measure. *J Gen Intern Med* Published Online First: 2001. doi:10.1046/j.1525-1497.2001.016009606.x
43 Bhana A, Rathod SD, Selohilwe O, *et al.* The validity of the Patient Health Questionnaire for screening depression in chronic care patients in primary health care in South Africa. *BMC Psychiatry* 2015;**15**:118. doi:10.1186/s12888-015-0503-0
44 Cholera R, Gaynes BN, Pence BW, *et al.* Validity of the patient health questionnaire-9 to screen for depression in a high-HIV burden primary healthcare clinic in Johannesburg, South Africa. *J Affect Disord* 2014;**167**:160–6. doi:10.1016/j.jad.2014.06.003
45 Osborn TL, Venturo-Conerly KE, Wasil AR, *et al.* Depression and Anxiety Symptoms, Social Support, and Demographic Factors Among Kenyan High School Students. *J Child Fam Stud* 2019;:1–12. doi:10.1007/s10826-019-01646-8
46 Marlow M, Skeen S, Grieve CM, *et al.* Detecting Depression and Anxiety Among Adolescents in South Africa: Validity of the isiXhosa Patient Health Questionnaire-9 and

For peer review only

For peer review only - http://bmjopen.bmj.com/site/about/guidelines.xhtml

Generalized Anxiety Disorder-7. *J Adolesc Heal* 2022;**0**. doi:10.1016/J.JADOHEALTH.2022.09.013
47 Spitzer RL, Kroenke K, Williams JBW, *et al.* A brief measure for assessing generalized anxiety disorder: the GAD-7. *Arch Intern Med* 2006;**166**:1092–7. doi:10.1001/ARCHINTE.166.10.1092
48 Connor KM, Davidson JRT. Development of a new resilience scale: the Connor-Davidson Resilience Scale (CD-RISC). *Depress Anxiety* 2003;**18**:76–82. doi:10.1002/DA.10113
49 Treynor W, Gonzalez R, Nolen-Hoeksema S. Rumination reconsidered: A psychometric analysis. *Cognit Ther Res* 2003;**27**:247–59. doi:10.1023/A:1023910315561
50 Tennant R, Hiller L, Fishwick R, *et al.* The Warwick-Dinburgh mental well-being scale (WEMWBS): Development and UK validation. *Health Qual Life Outcomes* 2007;**5**:1–13. doi:10.1186/1477-7525-5-63/TABLES/4
51 Kanter JW, Mulick PS, Busch AM, *et al.* The Behavioral Activation for Depression Scale (BADS): Psychometric properties and factor structure. *J Psychopathol Behav Assess* 2007;**29**:191–202. doi:10.1007/S10862-006-9038-5
52 Schweizer S, Parker J, Leung JT, *et al.* Age-related differences in affective control and its association with mental health difficulties. *cambridge.org* Published Online First: 2019. doi:10.1017/S0954579419000099
53 Jahanshahi M, Saleem T, Ho AK, *et al.* A preliminary investigation of the running digit span as a test of working memory. *Behav Neurol* 2008;**20**:17–25. doi:10.3233/BEN-2008-0212
54 White TL, Lejuez CW, de Wit H. Test-Retest Characteristics of the Balloon Analogue Risk Task (BART). *Exp Clin Psychopharmacol* 2008;**16**:565–70. doi:10.1037/a0014083
55 Fuhrmann D, Knoll LJ, Sakhardande AL, *et al.* Perception and recognition of faces in adolescence. *Sci Rep* 2016;**6**:33497. doi:10.1038/srep33497
56 Chierchia G, Fuhrmann D, Knoll LJ, *et al.* The matrix reasoning item bank (MaRs-IB): novel, open-access abstract reasoning items for adolescents and adults. *royalsocietypublishing.org* 2019;**6**. doi:10.1098/rsos.190232
57 Pettifor A, Rees H, Steffenson A, *et al.* HIV and sexual behaviour among young South Africans: a national survey of 15-24 year olds. *Reprod Heal Res Unit, Univ Witswatersrand* 2004.
58 Humeniuk R, Holmwood C, Beshara M, *et al.* ASSIST-Y V1.0: First-Stage Development of the WHO Alcohol, Smoking and Substance Involvement Screening Test (ASSIST) and Linked Brief Intervention for Young People. *J Child Adolesc Subst Abuse* 2016;**25**:384–90. doi:10.1080/1067828X.2015.1049395
59 Andersen S, Harrison GW, Lau MI, *et al.* Elicitation using multiple price list formats. *Exp Econ* 2006;**9**:383–405. doi:10.1007/s10683-006-7055-6
60 Kahneman D, Tversky A. Prospect theory: An analysis of decision under risk. In: *Experiments in Environmental Economics*. Taylor and Francis Inc. 2018. 143–72. doi:10.2307/1914185
61 StataCorp. Stata Statistical Software: Release 14. 2015.
62 Team RC. R: A Language and Environment for Statistical Computing. 2021.
63 Braun V, Clarke V. Using thematic analysis in psychology. *Qual Res Psychol* 2006;**3**:77–101. doi:10.1191/1478088706qp063oa
64 Arjadi R, Nauta MH, Scholte WF, *et al.* Internet-based behavioural activation with lay counsellor support versus online minimal psychoeducation without support for treatment

For peer review only - http://bmjopen.bmj.com/site/about/guidelines.xhtml

of depression: a randomised controlled trial in Indonesia. *The Lancet Psychiatry* 2018;**5**:707–16. doi:10.1016/S2215-0366(18)30223-2

65 Fu Z, Burger H, Arjadi R, *et al.* Effectiveness of digital psychological interventions for mental health problems in low-income and middle-income countries: a systematic review and meta-analysis. *The Lancet Psychiatry* 2020;**7**:851–64. doi:10.1016/S2215-0366(20)30256-X

66 Pettifor A, MacPhail C, Selin A, *et al.* HPTN 068: A Randomized Control Trial of a Conditional Cash Transfer to Reduce HIV Infection in Young Women in South Africa—Study Design and Baseline Results. *AIDS Behav* 2016;**20**:1863–82. doi:10.1007/S10461-015-1270-0/FIGURES/3

67 Faul F, Erdfelder E, Lang AG, *et al.* G*Power 3: A flexible statistical power analysis program for the social, behavioral, and biomedical sciences. *Behav Res Methods 2007 392* 2007;**39**:175–91. doi:10.3758/BF03193146

Figure 1
CONSORT Flow Diagram for the DoBAt Pilot Trial

For peer review only

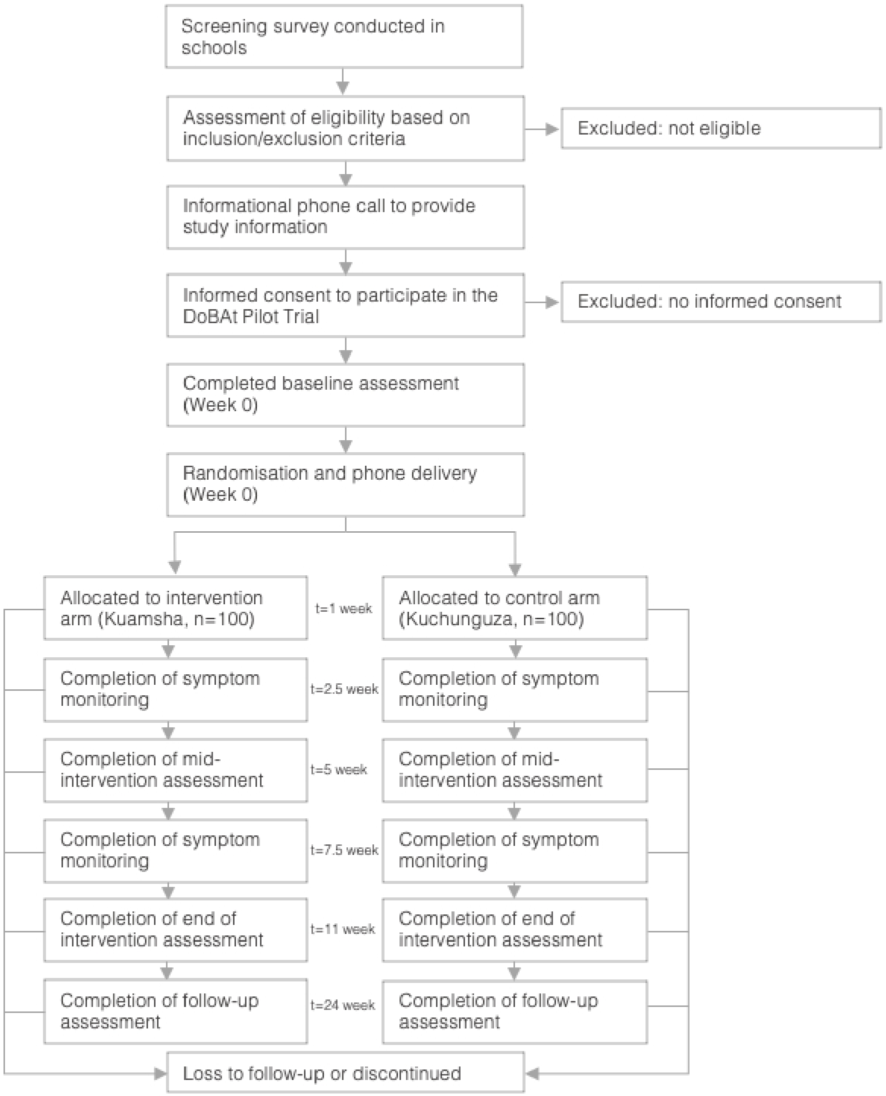

Figure 1. CONSORT Flow Diagram for the DoBAt Pilot Trial

232x263mm (72 x 72 DPI)

SUPPLEMENTAL MATERIAL

Digital delivery of Behavioural Activation therapy to overcome depression and facilitate social and economic transitions of adolescents in South Africa (the DoBAt study): protocol for a pilot randomised controlled trial

Table of Contents

Table 1. Trial Personnel and Committees **2**

Table 2. The Song Contest story **3**

Table 3. The Football Match story **4**

Table 4. Example mock-ups of the Kuamsha app **5**

Table 5. Trial registration data **7**

Example of the Participant Consent Forms **9**

DoBAt Study Statistical Analysis Plan **16**

For peer review only

Table 1. Trial Personnel and Committees

Committee	Members and responsibilities
**Trial Management Team (TMT)**	Comprised of the co-Principal Investigators, the Trial Manager, the Trial Psychologist, and other investigators. The TMT is responsible for the day to day running of the trial and meets on a weekly basis.
**Risk Management Team (RMT)**	The RMT consists of the Trial Manager (a Medical Doctor), the Trial Clinical Psychologist, the Trial Registered Counsellor, and a senior Field Supervisor. Supervision and oversight are provided by Prof. Alan Stein (Child and Adolescent Psychiatrist), Prof. Kathleen Kahn (Medical Doctor), Prof. Crick Lund (Clinical Psychologist) and Prof. Tholene Sodi (Clinical Psychologist). The RMT is responsible for assessing and managing all risks amongst screened and enrolled participants.
**Trial Steering Committee (TSC)**	Consists of senior academic clinicians and researchers including Prof. Roz Shafran (chair of the TSC, Professor of Translational Psychology, UCL), Prof. Jonathan Roiser (Professor of Cognitive Neuroscience, UCL), Prof. Soraya Seedat (Professor of Psychiatry, Stellenbosch University), and Prof. Jonathan Levin (Professor of Biostatistics, University of Witwatersrand). The TSC oversees the scientific conduct of the study and meets on a quarterly basis throughout the trial.
**Independent Data and Safety Monitoring Board (DSMB)**	Comprised of Prof. Bonginkosi Chiliza (chair of the DSMB, Chief Specialist and Head of the Department of Psychiatry at the University of KwaZulu-Natal), Dr. Elizabeth George (Statistician, MRC Clinical Trials Unit at University College London), Prof. John Joska (HIV Mental Health Research Unit, Division of Neuropsychiatry, University of Cape Town), and Prof. Marguerite Schneider (Department of Psychiatry and Mental Health, University of Cape Town). The DSMB oversees the conduct and safety of the trial. Meetings will be held before recruitment begins, midway through recruitment, and at the end of the 11-week assessment period. A formal interim analysis will not be conducted.

For peer review only

Table 2. The Song Contest story

Learning module	Description of each module	BA Learning principles
**Episode 1 ("Pick a team")**	Students find out about a school Song Contest. The winning prize is a voucher to shop for a new outfit at the mall. User chooses the main character's name and picks two teammates to join the Song Contest.<br>Main character gets anxious about the idea of performing in front of everyone else. Christine (teacher) talks about the benefits of stepping outside one's comfort zone and offers her support.<br>At the end of the module, the user will be asked to set a goal to work on over the 10 weeks of intervention.	Absorption<br>TRAP-TRAC<br>Sleep<br>Self-confidence<br>Relapse prevention
**Episode 2 ("Find a cool tune")**	First practice session with group which didn't go well because one teammate fell asleep. Main character is frustrated and decides to ask Christine for advice. After hearing that teammate's grandmother ill, main character showed leadership and compassion in how they handled the situation.	
**Episode 3 ("The Lure")**	The team worked well together and made progress on the song. At the end of the practice, Prince (desirable character) invites the main character to go to the tavern. Main character is asked to think about the consequences of their actions but decides to go anyway.	
**Episode 4 ("The Fallout")**	The next day the main character is exhausted, hungover, and unable to concentrate. They arrive late to practice session without homework done. Teammates get annoyed. Main character decides to apologise instead of avoiding the problem and their teammates are forgiving. Main character learns about the importance of sleep and goes to sleep early.	
**Episode 5 ("The Return")**	Main character sleeps and feels recharged. Team is happy with the practice. Song is completed. Main character learns that they have to give a presentation if they win and asks Christine for advice. Main character learns ways to deal with being nervous and how to feel more self-confident.	
**Episode 6 ("The Big Day")**	Team feels nervous before the show. They perform and win the prize. Group reflects on journey.	

For peer review only - http://bmjopen.bmj.com/site/about/guidelines.xhtml

Table 3. The Football Match story

Learning module	Description of each module	BA Learning principles
**Episode 1 ("Pick a team")**	Main character is the striker for the local team. User chooses the main character's name and picks a name for the football team. User gets introduced to the football task. At the end of the module, the user will be asked to set a goal to work on over the 10 weeks of intervention.	Absorption<br>Rumination<br>TRAP-TRAC<br>Problem-solving<br>Negotiation
**Episode 2 ("Game Over")**	Main character misses some important shots. Shane (antagonistic character) is a discouraging main character because of their poor performance. Team loses the game. Main character leaves the match alone feeling upset and discouraged, ruminating over her performance during the match. Bird offers different, more positive perspective on performance but the main character not in a frame of mind to believe it.	
**Episode 3 ("Hide away")**	Main character is frustrated, misses school and gets a detention. Main character feels a bit better after friends visit her/him at her house, but apprehensive about going to school. Main character plays the football task which makes them feel energised enough to do their homework.	
**Episode 4 ("That's okay")**	Main character returns to school feeling more positive after a good night's sleep. Main character handles Shane well, but their mood takes a dip. Main character decides to speak with Coach Bayer after school about their performance in the match. Main character learns that mistakes can be useful opportunities to learn, instead of something to avoid. Main character practises what they've learnt and starts to feel more confident.	
**Episode 5 ("Keep practising")**	Main character speaks with friends before practice about what they learnt from Coach Bayer and asks for help in dealing with Shane. With their supportive presence, main character gets Shane to agree to stay away from them so that they can focus on football and winning. Main character feels confident, keeps practising, and feels ready for the re-match.	
**Episode 6 ("The Big Match")**	It all came together for the main character, stepping out onto the pitch with new confidence. Main character keeps calm and meets their mistakes with curiosity instead of frustration. Main character scores the winning goal.	

For peer review only

For peer review only - http://bmjopen.bmj.com/site/about/guidelines.xhtml

Table 4. Example mock-ups of the Kuamsha app

Component	Description	Example
Home screen	This is the first screen that users will see as they open the Kuamsha app. Users will have the option to play through the stories, monitor their mood, play absorbing activities to improve focus, or report on their weekly activities (see below for further details on each of these components).	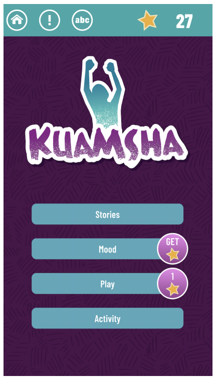
Log-in unlock code	Kuamsha is password-protected. Users will be asked to enter a password every time they access the app.	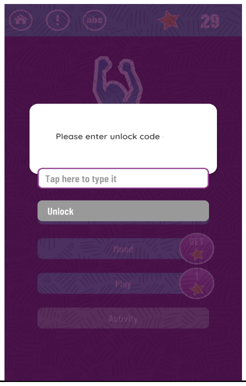
Language selector	Users will be able to select their preferred language. All the text in the app underwent two rounds of translation and has been checked by a clinical psychologist for accuracy.	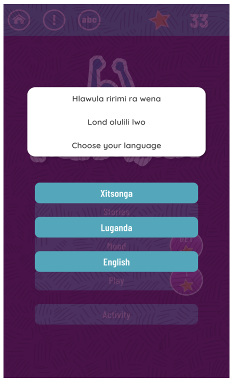
Story Selection	The core of the game consists of a choice between two narrative stories. Each consists of 6 modules that are played in sequential order. It is possible to begin one story and then switch to the other. During gameplay points are earned for the choices made and by completing other core game elements described below.	

For peer review only

		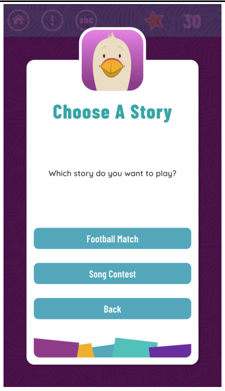
Mood monitoring	Mood monitoring is an important part of BA and can be used as a tool for understanding how your mood is impacted and changes depending on the activities you are engaged in. Every time a user complete mood monitoring, they receive in-app points. Participants are asked to monitor their mood at various times (before and after each module, when they report a homework activity, and when they complete an absorbing activity).	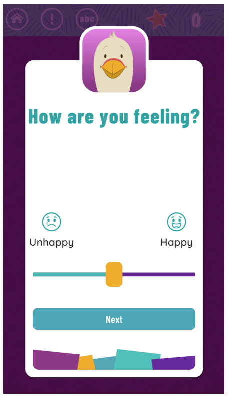

For peer review only

Table 5. Trial registration data

Data category	Information
**Primary registry and trial identifying number**	South African National Clinical Trials Registry (DOH-27-112020-5741); Pan African Clinical Trials Registry (PACTR202206574814636)
**Date of registration in primary registry**	19th November 2020
**Ethics reference**	MED20-05-011 / OxTREC 34-20
**Protocol version**	v1.2 19/04/2021
**Protocol amendments**	Important protocol amendments such as changes to eligibility criteria, outcomes or analysis will be reported to investigators, the TSC, and both ethics committees in writing, and relayed to study participants at the soonest availability.
**Funder**	MRC Newton Fund UK-South Africa Joint Initiative on Mental Health (MR/S008748/1)
**Sponsor**	Wits Health Consortium (Pty) Limited, University of Witwatersrand<br>31 Princess of Wales Terrace, Parktown, Johannesburg, 2193<br>011 274 9200
**Trial Title**	Digital delivery of Behavioural Activation therapy to overcome depression and facilitate socio-economic transitions of adolescents in South Africa: pilot Randomised Control Trial
**Short Title**	DoBAt Study
**Countries of recruitment**	South Africa
**Health condition(s) or problem(s) studied**	Depression
**Intervention**	All participants in the intervention and control arms will be given a Samsung Galaxy A2 Core Android smartphone and receive active symptom monitoring via text messages sent to the smartphone every 2.5 weeks.<br>• The intervention arm will receive Behavioural Activation (BA) therapy via a smartphone application (the *Kuamsha* app) and supported by weekly phone calls from Trained Peer Mentors, implemented over 10 weeks.<br>• The control arm will receive a smartphone application (the *Kuchunguza* app) containing six module video clips from WildEarth-SafariLive, a locally produced wildlife series.
**Trial Participants**	Adolescents aged 15-19 with mild to moderately-severe depression based on scores between 5 and 19 on the 9-item Patient Health Questionnaire-Adolescent version (PHQ-A), that live in the MRC/Wits-Agincourt study area, Bushbuckridge, and from whom we obtain written informed assent and consent (including parental consent if <18 years).
**Study type**	Two-arm single-blind individually randomised controlled pilot trial
**Date of first enrolment**	25th November 2021
**Trial Status**	Enrolment of the first participant occurred on the 25th November 2021. We expect to finish data collection in January 2023.
**Target sample size**	200
**Recruitment status**	Recruiting
**Primary outcomes**	1) to determine the feasibility and acceptability of the intervention<br>2) to provide preliminary evidence on the initial efficacy (direction and magnitude) of any effects of the intervention on depressive symptoms
**Key secondary outcomes**	1) to pilot a range of mental health, social-affective cognition, risky behaviours, and socioeconomic measures

For peer review only - http://bmjopen.bmj.com/site/about/guidelines.xhtml

	2) to collect descriptive data on trial procedures such as recruitment, retention, data collection, randomisation and blinding to inform the development of a further larger trial.
**Data sharing statement**	Individual participant quantitative data that underlie the results reported in each publication arising from the trial will be made available after deidentification. Any video, audio or qualitative data will not be available. Data will be available beginning 9 months and ending 36 months following main article publication to researchers who provide a methodologically sound proposal that purposes to achieve aims in the approved proposal and /or for individual participant data meta-analysis. Data is documented and stored on the MRC/Wits-Agincourt Data Repository with a digital object identifier (doi) and can be accessed with permission and in line with with MRC/Wits-Agincourt policies and procedures. Data requestors will need to sign a data access agreement before any data can be shared. In addition, Study Protocol and Statistical Analysis Plan documents will be available.

For peer review only

For peer review only - http://bmjopen.bmj.com/site/about/guidelines.xhtml

Example of the Participant Consent Forms

PARTICIPANT INFORMATION SHEET: ADOLESCENTS UNDER 18 YEARS OLD

Study title: Digital delivery of Behavioural Activation therapy to overcome depression and facilitate social and economic transitions of adolescents in South Africa (DoBAt study)

Good day,

I am Mr/Mrs____ from the MRC/Wits-Agincourt Research Unit.

We would like to ***invite*** you to take part in a research study. Please read this carefully. **Ask us** if there is anything you do not understand and **take time** to decide whether or not you want to participate. It is important that you understand what it will mean if you take part, including what could go wrong. You can talk about this with your family, friends, parents, or anyone else you want to. You may not take part in another study while you take part in this study. Please read through this sheet and ask the study team about anything that you do not understand.

1. **What is the study about?**

We are doing research on adolescents' wellbeing. In this study we want to see whether it is possible to improve low mood, or symptoms of depression, by using a smartphone application (app) combined with phone calls from a peer mentor. The app will be set up like a game and teach ideas based on Behavioural Activation (BA), a strategy to help treat low mood. A peer mentor is someone who speaks Xitsonga and is a few years older than you.

2. **Why have I been invited to take part?**

You have been invited to take part because you recently participated in a survey at your high school about your thoughts, feelings and behaviours. Based on your answers, we think you may be experiencing low mood, which is one of the things we are looking at in this study.

In order to take part in the study, you must be aged 15-19, enrolled in Grades 9-11 and speak and read Xitsonga. You must also live in the local area for at least 12 weeks after the study begins.

You can't take part in the study if you have a problem with your health that would make it hard to participate. You also can't take part if you are seeing someone (like a counsellor or doctor) for problems with your mood or mental health already. You can ask the researcher about this if you're not sure.

3. **How big is the study and how long will it last for?**

There will be about 200 adolescents taking part. The study will take place during 2021 and 2022. If you decide to participate, you would be involved for 6 months.

4. **What is involved for me?**

If you are interested in taking part in the study, a researcher will arrange a time to come to your house. They will go over information about the study with you and your parent/guardian. You can ask any questions you have about the study at any point. After this, if you decide to take part, you will be asked to sign an electronic form to say that you agree to participate (an *assent form*). Because you are under 18, your parent/guardian will also need to give permission by signing a *consent form* before you can participate. The researcher will ask you some questions about your health and wellbeing to check that you are able to participate in the study. They will also ask you to answer some questions and do some tasks to measure

For peer review only - http://bmjopen.bmj.com/site/about/guidelines.xhtml

your thoughts, feelings and behaviours (see more on 'assessments' below). We will also ask your parent/guardian some questions about your household.

After this, the researcher will find a time to come back to your house to give you the smartphone. The smartphone will have an app installed which you will use during the study. The researcher will show you how to use the phone and the app, and give you a chance to ask any questions. They will also tell you who to ask for help if you have a problem with the smartphone or the app, or if your mood is very low.

Once you are signed up for the study, you will find out whether you are in Group 1 (Kuamsha app, the type of support we are testing) or Group 2 (Kuchunguza app, more on this below). You have a 50/50 chance of getting one or the other. You will then use the smartphone for 10 weeks. What you do with the smartphone depends on which group you are in:

Group 1 (Kuamsha app)

If you are in Group 1, your smartphone will have an app on it. The app will be set up like a game and teach ideas based on Behavioural Activation (BA), a strategy that teaches skills to cope with your problems and low mood. The app is like a game and you can play through six 'episodes' which are each part of a story. Each episode will take around 10 minutes and you will get some weekly activities to work on at the end. The weekly activities will take around 20 minutes and involves different activities like going for a walk, setting up a bedtime routine, or listening to music. The app will also ask you to monitor your mood at different times during the study. The researcher will also put you in touch with a peer mentor, which is someone a few years older than you who will help you with the app and the weekly activities. The peer mentor will keep your information private and confidential. The peer mentor will first call you for an 'introductory phone call', where you will get the chance to introduce each other and answer any questions you may have. After this phone call, you will speak to the peer mentor for 15-20 minutes for each episode that you finish.

Group 2 (Kuchunguza app)

If you are in Group 2, your smartphone will have an app with videos to watch (6 in total). Each video should take around 15-20 minutes to watch.

Assessments (Groups 1 and 2)

We will ask you questions at different points during the study. The first time (week 0) the researcher visits your house, they will ask you some questions on your thoughts, behaviours, past experiences, employment, consumption, and aspirations for the future. You will listen to some of these questions using headphones and click your answers on a tablet. This means that you won't have to say anything out loud (so your answers are private). You will also use the tablet to do some tasks which measure memory, learning, and decision-making. Some of these tasks will give you a chance to win mobile data. After 11 weeks (week 11), the researcher will come back to your house to do these questions again.

During the study, the researcher will also call you or send you a text message (SMS) every 2-3 weeks to ask you some questions about your symptoms. This will also allow us to check on how you are doing.

At the end of the study (week 24), the researcher will call you or send you a text message to ask you some of the questions you were asked at the beginning of the study. The researcher will ask some participants (but not everyone) to take part in a one-on-one interview on a separate occasion. If you are asked to take part in an interview, the researcher will ask you questions about what it was like participating in the study. This will last around 1 hour.

During the study, we will use information about your household which was collected by the MRC Wits-Agincourt Research Unit. This was collected during the census, also known as the Health and Demographic Surveillance System (HDSS).

The timeline shows what will take place in the study, when and where:

For peer review only - http://bmjopen.bmj.com/site/about/guidelines.xhtml

Timeline for Group 1:

WEEK 0	WEEK 1	WEEK 2	WEEK 3	WEEK 4	WEEK 5	WEEK 6	WEEK 7	WEEK 8	WEEK 9	WEEK 10	WEEK 11	WEEK 24
**Consent, Assent, Assessment 1** Home visit 2 hours<br>**Phone delivery** Home visit 1 hour<br>**Introductory Phone call Peer mentor** 15-20 mins	**Episode 1** Kuamsha app 10 minutes<br>**Peer mentor call 1** Phone call 15-20 minutes	**Episode 2** Kuamsha app 10 minutes<br>**Peer mentor call 2** Phone call 15-20 minutes	**Episode 3** Kuamsha app 10 minutes<br>**Peer mentor call 3** Phone call 15-20 minutes		**Assessment 2** Phone/ electronically 30 minutes	**Episode 4** Kuamsha app 10 minutes<br>**Peer mentor call 4** Phone call 15-20 minutes	**Episode 5** Kuamsha app 10 minutes<br>**Peer mentor call 5** Phone call 15-20 minutes	**Episode 6** Kuamsha app 10 minutes<br>**Peer mentor call 6** Phone call 15-20 minutes			**Assessment 3, Interview** Home visit 2 hours	**Assessment 4** Phone/ electronically 1 hour
		**Symptoms monitoring** SMS 5 minutes					**Symptoms monitoring** SMS 5 minutes					

Timeline for Group 2:

WEEK 0	WEEK 1	WEEK 2	WEEK 3	WEEK 4	WEEK 5	WEEK 6	WEEK 7	WEEK 8	WEEK 9	WEEK 10	WEEK 11	WEEK 24
**Consent, Assent, Assessment 1** Home visit 2 hours<br>**Phone delivery** Home visit 1 hour	**Video 1** Kuchunguza app 20 minutes	**Video 2** Kuchunguza app 20 minutes	**Video 3** Kuchunguza app 20 minutes		**Assessment 2** Phone/ electronically 30 minutes	**Video 4** Kuchunguza app 20 minutes	**Video 5** Kuchunguza app 20 minutes	**Video 6** Kuchunguza app 20 minutes			**Assessment 3, Interview** Home visit 2 hours	**Assessment 4** Phone/ electronically 1 hour
		**Symptoms monitoring** SMS 5 minutes					**Symptoms monitoring** SMS 5 minutes					

5. Do I have to participate?

No. It is up to you whether you decide to take part. If you decide that you don't want to take part, that's okay. You can decide that you don't want to take part at any time – before the study, during the study, or afterwards. You don't need to give a reason, and no one will be upset or annoyed. Deciding not to take part won't affect your grades, school, medical care or any other services. You may discuss whether or not to take part with your family, friends, or teachers.

6. Will taking part cause me any problems?

Whilst we hope that participating in the study is a positive experience for you, we know that some of the questions might make you feel upset or frustrated. If any question makes you uncomfortable, you can skip the question. If you feel upset by anything in the study you can talk to the research staff who will do their best to help you feel better.

We also realise that in some places people can have negative ideas around people who have low mood. We will make sure to keep this information very private and confidential so that no one outside of the study team and your parent/guardian knows you are participating in the study.

We think it is very unlikely that you will suffer any harm from taking part in the study, but it is good to know that the University of Witwatersrand has insurance in case anything did happen to you.

7. Will taking part help me at all?

We know that Behavioural Activation (BA) can sometimes improve young people's mood. We're not sure whether it would improve your mood or not, because it is one of the things we are testing in the study. Your participation in the study will also help us to understand the needs of adolescents with low mood and to develop a treatment that works for them.

For peer review only - http://bmjopen.bmj.com/site/about/guidelines.xhtml

8. What happens to my information? Will it be kept confidential?

All the information you share with us is private and confidential. That means that we would not name you or tell your parents, friends, or teachers what you said when we meet. This includes your answers to the questions, what you say during the phone calls and interviews. We will also take out any information that would identify you and keep all of the information about you safely in a different place to your answers to the study questions. Your information will be stored on a password protected computer for at least 10 years. If you stop taking part, we will automatically destroy any data related to you, unless you give us permission not to.

The only times we would break our confidentiality is if we were worried that you – or someone in contact with you – was at risk of serious harm. If that happens, we would talk with you about it so that we could make a plan together to get you the help and support you need. We might also need to break the confidentiality if it was required by law or by the Human Research Ethics Committee at the University of the Witwatersrand.

If you are in Group 1 and receive calls from the peer mentor, we will record the peer mentor calls. We will also record the interviews that happen in Week 11, if you are asked to do one. We will write down what was said, taking care to take out your name and any other information which could identify you. Once we write everything down, we will delete the recordings. We might include quotes of what you say when we publish results of the study, but we will make sure that no one can tell who said them. You will be able to choose a name that we can use instead of your real name (a *pseudonym).*

9. Will I have to pay for anything? Will I be paid to take part?

You do not have to pay to take part. You will also not be paid. As part of the study, we will give you a smartphone to use, which you can keep at the end of the study. We will cover the cost of mobile data for you to use the app. We will also offer you a snack and a small amount of mobile data when we visit your home to do the questionnaires. You will also receive 10ZAR mobile data each time you complete a questionnaire by text message (SMS).

If you are in Group 1, the peer mentor will call you so that you do not have to use your own airtime.

10. Will we publish the results of this study?

Yes, we will publish the results of this study in academic journals. We will also provide 'fact sheets' with information about the results of the study at community meetings and to service providers, village leaders and other people locally. We will publish only findings that are important and apply to many people, and we will not publish any information that would identify you. You can get in touch with us if you would like us to tell you the results of the study directly.

11. Who has approved this study?

This study has been approved by the Human Research Ethics Committee (Medical) of University of Witwatersrand, Ehlanzeni District and Mpumalanga Provincial Departments of Health and Education, and the Oxford Tropical Research Ethics Committee. The purpose of these committees is to protect the integrity (honesty) of the research, and the rights and dignity of all people who agree to participate in a research project.

12. Who can I talk to for more information or to report a problem?

If you have any questions about this study or you are worried about something, please call us:

- Professor Kathy Kahn, Principal Investigator, tel: 011 717 2617, email: Kathleen.Kahn@wits.ac.za
- Dr Bianca Moffett, Project Manager, email: Bianca.Moffett@wits.ac.za.
- If you need assistance in Xitsonga, you can call this number: 0824068527

For peer review only - http://bmjopen.bmj.com/site/about/guidelines.xhtml

Within 10 days, they should say that they have received your concern and tell you how they are thinking of dealing with it.

If you are feeling upset or distressed, you can also contact Sindile Hlatswayo, the social worker at Agincourt CHC, on 013 708 1471/7940 during office hours, or book an appointment to see the clinical psychologist at Mapulaneng Hospital by contacting 013 799 0214.

If you have any concerns about the way the study is being conducted, please contact the Chairperson of the University of the Witwatersrand Ethics Committee, Dr Clement Penny, tel: 011 717 2301, email: Clement.Penny@wits.ac.za. The telephone numbers for the Ethics Committee secretariat are 011 717 2700/1234 and the e-mail addresses are Zanele.Ndlovu@wits.ac.za and Rhulani.Mukansi@wits.ac.za.

13. Data protection

The University of the Witwatersrand is responsible for the way your data is used in the study. They will process your data for this research, which is performed in the public interest. More information about your rights with regard to personal data can be seen online at: https://compliance.web.ox.ac.uk/individual-rights.

Thank you for reading this Study Information Sheet.

Date:

For peer review only - http://bmjopen.bmj.com/site/about/guidelines.xhtml

ASSENT FORM: ADOLESCENTS UNDER 18 YEARS OLD

Study title: Digital delivery of Behavioural Activation therapy to overcome depression and facilitate social and economic transitions of adolescents in South Africa (DoBAT)

1. Have you had enough time to read the Participant Information Sheet or has someone explained this study to you?	Yes / No
2. Do you understand what this study is about?	Yes / No
3. Have you asked all the questions you want?	Yes / No
4. Do you understand it's okay to stop taking part in this study at any time?	Yes / No
5. Do you understand that you will not receive any payment to be in the study and that participating in the study will not cost you anything but your time?	Yes / No
6. Do you understand who will have access to your information, how it will be stored, and what will happen to it at the end of the study?	Yes / No
7. Do you understand that we will keep all your information anonymous and confidential?	Yes / No
8. Do you understand that your parent/legal guardian will also need to give permission for you to take part in this study?	Yes / No
9. Do you understand that you can contact the research team if you have any questions?	Yes / No
10. Are you happy to take part?	Yes / No

PARTICIPANT

______________	______________	______________	______________
Name of participant	Signature or mark	Date and time	Phone number (if available)

STUDY TEAM

______________	______________	______________
Name of person taking consent	Signature or mark	Date and time

For peer review only - http://bmjopen.bmj.com/site/about/guidelines.xhtml

AUDIO RECORDING

As mentioned on the information sheet, we may ask you to do an interview after 3 months, which will be recorded. If you are in Group 1, we will also record the conversations you have with the peer mentor. We will type up this information as soon as possible and then delete the recording. All the information you say with us is private and confidential. We may use quotes of what you say when we write about the study, but we will take care to take out any information which could identify you. We will keep all of the information about you safely and in a different place to the recording.

Are you happy for your voice to be recorded?	Yes / No

PARTICIPANT

Name of participant	Date and time	Signature or mark

STUDY TEAM

Name of person taking consent	Date and time	Signature

Researcher contact details:

- Professor Kathy Kahn, Principal Investigator, tel: 011 717 2617, email: Kathleen.Kahn@wits.ac.za.
- Dr Bianca Moffett, Project Manager, email: Bianca.Moffett@wits.ac.za.
- If you need assistance in Xitsonga, you can call this number: 0824068527
- Dr CB Penny, Chairperson of the Human Research Ethics Committee (Medical) at the University of Witwatersrand, tel: 011 717 2301, e-mail: Clement.Penny@wits.ac.za
- Ms. Zanele Ndlovu or Mr. Rhulani Mkansi, Committee Secretariat, tel: 011 717 2700/1234, e-mail: Zanele.Ndlovu@wits.ac.za or Rhulani.Mkansi@wits.ac.za

For peer review only - http://bmjopen.bmj.com/site/about/guidelines.xhtml

DoBAt Study Statistical Analysis Plan

Statistical Analysis Plan

PILOT TRIAL FULL TITLE	Digital delivery of Behavioural Activation therapy to overcome depression and facilitate social and economic transitions of adolescents in South Africa<br>(The DoBAt study)
SAP VERSION	Version 1.0
SAP VERSION DATE	3rd November 2022
PILOT TRIAL STATISTICIAN	Prof. Eustasius Musenge<br>School of Public Health<br>University of the Witwatersrand
Protocol Version (SAP associated with)	Version 1.2 (19/04/21)
PILOT TRIAL PRINCIPAL CO-INVESTIGATOR	Prof. Kathleen Kahn, University of the Witwatersrand<br>Prof. Alan Stein, University of Oxford
SAP AUTHOR(s)	Ms. Julia Ruiz Pozuelo (lead author)<br>Dr. Kate Orkin<br>Prof. Eustasius Musenge

For peer review only

Abbreviations

AE	Adverse Event
BA	Behavioural Activation
EF	Executive Function
DSMB	Data and Safety Monitoring Board
ESoC	Enhanced Standard of Care
ITT	Intention to treat population
MRC	Medical Research Council
PHQ-A	Patient Heath Questionnaire Adolescent version
SAE	Serious Adverse Event
SAP	Statistical Analysis Plan
TSC	Trial Steering Committee

For peer review only

1. Introduction

This document details the proposed presentation and analysis for the final trial report presenting the results from an MRC-funded pilot randomised controlled trial of a digital intervention for adolescents with depression in the rural Bushbuckridge Local Municipality of Mpumalanga Province, South Africa.

This document describes the objectives, intervention, core pre-registered hypotheses we commit to testing, the definitions of the outcomes of interest, and estimation and inference methods. We do not rule out the possibility of running other estimations. However, we will make clear in the papers which estimations are specified in this pre-analysis plan and which are not.

We have not yet completed recruitment at the time of lodging this analysis plan.

2. Background information

2.1 Rationale

Depression peaks during adolescence, with up to 20% of adolescents affected in rural South Africa and with few having access to treatment (Goin et al., 2019; Kessler et al., 2005). Untreated depression exerts a substantial economic toll as it impairs functioning, interferes with schooling, and impacts adolescents' ability to build successful relationships and make important life decisions (Thapar et al., 2012). These impairments have a greater impact on adolescents in low- and middle-income countries due to the additional adversities they face and the lack of available, effective treatments. Evidence is urgently needed for cost-effective and scalable interventions targeting adolescent depression.

2.2 Study Objectives

In this pilot study, we are exploring if a smartphone app that delivers Behavioural Activation (a psychological therapy for depression) can reduce depressive symptoms among adolescents living with depression in the Bushbuckridge area.

The co-primary objectives of the study are:
1) to determine the feasibility and acceptability of a digitally delivered Behavioural Activation therapy intervention for adolescents living with depression in the Bushbuckridge sub-district of Mpumalanga province, South Africa; and
2) to provide preliminary evidence on the efficacy (direction and magnitude) of any effects of the intervention on depressive symptoms amongst adolescents in the intervention arm compared to the control group.

The secondary objectives are:
1) to pilot locally adapted outcome measures of mental health, social-affective cognition, risky behaviours, and socioeconomic measures; and
2) to collect descriptive data on trial procedures such as recruitment, retention, data collection, randomisation, and blinding to inform key parameters in developing a future large-scale trial.

2.3 Trial reporting

This protocol is reported in accordance with the Standard Protocol Items: Recommendations for Intervention Trials (SPIRIT) 2013 statement (Chan et al., 2013). The final analysis for the main

For peer review only

results will be conducted once all the end-of-intervention assessments (week 11) have been completed.

2.4 Trial committees

Trial Steering Committee

The Trial Steering Committee (TSC) consists of Prof. Roz Shafran (chair of the TSC, Professor of Translational Psychology, UCL), Prof. Jonathan Roiser (Professor of Cognitive Neuroscience, UCL), Prof. Soraya Seedat (Professor of Psychiatry, Stellenbosch University), and Prof. Jonathan Levin (Professor of Biostatistics, University of Witwatersrand). The TSC oversees the scientific conduct of the study and meets on a quarterly basis.

Data and Safety Monitoring Board

The Data and Safety Monitoring Board (DSMB) is comprised of Prof. Bonginkosi Chiliza (chair of the DSMB, Chief Specialist and Head of the Department of Psychiatry at the University of KwaZulu-Natal), Dr Elizabeth George (Statistician, MRC Clinical Trials Unit at University College London), Prof. John Joska (HIV Mental Health Research Unit, Division of Neuropsychiatry, University of Cape Town), and Prof. Marguerite Schneider (Department of Psychiatry and Mental Health, University of Cape Town). The DSMB oversees the protection of participant safety. Meetings will be held before recruitment begins, midway through recruitment, and at the end of the 11-week assessment period. A formal interim analysis will not be conducted.

3. Intervention

All participants in the intervention and control arms will be given an entry-level Samsung Galaxy A2 Core Android **smartphone**, which they can keep at the end of the study. Furthermore, participants in both groups 200MB of **mobile internet data** at six different points (0, 2.5, 5, 7.5, 11, and 24 weeks) to ensure they have data to use the app and complete the online surveys.

Participants in both arms will receive **active symptom monitoring** via text messages sent to the smartphone every 2.5 weeks. Any adolescents who develop severe depression or high-risk suicidal ideation will be assessed by the Risk Management Team and referred to local clinical services. Adolescents started on anti-depressant medication or receiving psychological therapy because of these referrals will not be discontinued (we will note concomitant care and examine this using sensitivity analyses).

3.1 Control arm: Enhanced Standard of Care (ESoC)

The non-intervention arm will receive a control app (the *Kuchunguza app*) containing six video clips from *WildEarth-SafariLive*, a locally produced wildlife series. Each video clip takes approximately 15-20 minutes to complete and allows users to explore the African wilderness while listening to calming and atmospheric sounds.

In the given context, the control represents an enhancement of standard care since most adolescents with depression would not usually receive any intervention or active symptom monitoring and referral.

3.2 Intervention arm: Kuamsha programme

For peer review only - http://bmjopen.bmj.com/site/about/guidelines.xhtml

The intervention arm will receive six modules of Behavioural Activation (BA) therapy via a smartphone application (the *Kuamsha app*) supported by trained Peer Mentors, implemented over ten weeks. The app and phone calls from the Peer Mentors comprise the Kuamsha programme.

The Kuamsha app is primarily an interactive narrative game comprising six tailored modules (sessions) that contain Behavioural Activation's core principles integrated into a gamified format.[1] Each module takes approximately 15-20 mins to complete. They cover topics such as identifying and engaging in meaningful activities, and using strategies to overcome barriers, for example, problem-solving, effective communication, getting enough sleep, and disengaging from rumination. Each module is followed by a homework activity where the participant is encouraged to reflect on the BA principles outlined in each module and think about ways to apply them to their own lives. Participants will be asked to report how often they completed the homework activities and their mood as they did them. The app includes game design elements to stimulate motivation and performance, including character personification, in-app points, and reminders/ notifications. The Kuamsha app has been developed through extensive formative research and iterative user-centred design with adolescents in the study area to increase usability and acceptability.

The Kuamsha app will be supplemented by brief phone calls (15 mins per module) from Trained Peer Mentors. There will be seven calls in total, including an introductory phone call and six calls to cover the content of each module. Peer Mentors will attempt to reach participants by calling up to 5 times per week. The role of the Peer Mentors is mainly to support adherence and compliance with the app and to troubleshoot problems related to the use of the app and implementation of the homework activities. They will be trained not to provide additional advice or counselling and conduct their calls according to a pre-determined checklist of activities to help ensure fidelity. Peer Mentors will be Xitsonga-speaking students or recent graduates from the department of psychology or social work at an accredited South African university. They will be trained and supervised by the Trial Psychologist according to the training manual developed specifically for the intervention.

Table 1 shows DoBAt's time schedule of enrolment, interventions, assessments, and visits for participants.

4. Study methods

4.1 Study design

The DoBAt study is a two-arm single-blind individual-level randomised controlled pilot feasibility trial. A total of 200 adolescents will be recruited (1:1 allocation ratio).

4.2 Recruitment and study timeline

Participants will be recruited through a two-stage recruitment process. The first stage of recruitment (Phase 1) will consist of a screening survey conducted in schools to identify adolescents with symptoms of mild to moderately severe depression. The second stage of recruitment (Phase 2) will be done with adolescents who score between 5 and 19 on the Patient Health Questionnaire Adolescent version (PHQ-A) and who also meet the other eligibility criteria.

---

[1] See (Jacobson et al., 2006) for a description of Behavioural Activation.

For peer review only - http://bmjopen.bmj.com/site/about/guidelines.xhtml

The pilot trial consists of an 11-week intervention treatment phase, and participants will be followed up for a further 13 weeks after completing the intervention (i.e., a total of 24 weeks). Primary outcomes will be assessed at the end-of-intervention assessment (week 11) by blinded outcomes assessors, and qualitative interviews will be conducted with a sub-sample of participants. Table 1 indicates the time schedule of enrolment, interventions, assessments, and visits for participants, and Figure 1 shows the flow of participants.

4.3 Study setting

The study will be based in the Bushbuckridge sub-district of Mpumalanga Province, South Africa. Since 1992 the MRC/Wits Rural Public Health and Health Transitions Research Unit (MRC/Wits-Agincourt) has collected population data, with vital events (pregnancy outcome, deaths, in- and out-migration) and household composition updated annually through its Health and socio-Demographic Surveillance System (HDSS). The total population under surveillance is currently ~116,000 inhabitants residing in 31 contiguous villages (Kahn et al., 2012).

4.4 Eligibility criteria

To be eligible for inclusion in the pilot trial participants must: 1) be between 15 and 19 years of age and in grades 9 to 11 at the beginning of the study; 2) have symptoms of mild to moderately-severe depression indicated by a score between 5 and 19 on the Patient Health Questionnaire Adolescent Version (PHQ-A); 3) be able to read sufficiently in the local language (Xitsonga) to use the Kuamsha app; 4) intend to continue living in the study site for 12-weeks after the baseline assessment; and 5) provide written informed assent/consent to participate in the study, as well as parent/guardian consent if younger than 18 years.

Participants will be excluded if they: 1) have symptoms of severe depression as indicated by a score of >19 on the PHQ-A; 2) have current suicidal ideation with specific plans and means identified; 3) are receiving psychological treatment for a mental health condition at the time of enrolment; 4) have been hospitalised for at least five days for a severe psychiatric illness (specifically Bipolar Mood Disorder, Schizophrenia or other Psychotic Disorders) or life-threatening or other serious medical illness; 5) have a history of Bipolar Mood Disorder, Schizophrenia or other Psychotic Disorders; 6) lack capacity to consent to research participation. Participants excluded for points 1) and 2) will be assessed by the Risk Management Team and referred to local clinical services as per prior arrangements with local service providers.

4.5 Allocation

Following the baseline assessment, participants will be randomly assigned to the intervention or control arm with a 1:1 allocation using a computerised minimisation algorithm, balanced by sex (male or female) and severity of depressive symptoms (<10 on the PHQ-A or ≥10 on the PHQ-A). Both variables will be collected during the first stage of recruitment (Phase 1). The minimisation algorithm was generated by the Centre for Healthcare Randomised Trials (CHaRT) at the University of Aberdeen. Participants will be allocated using CHaRT's online software and the trial manager will oversee enrolling participants as per assignment.

4.6 Blinding

This is a single-blind study, with all fieldworkers conducting outcomes assessments and other investigators remaining blinded.

For peer review only - http://bmjopen.bmj.com/site/about/guidelines.xhtml

Participating adolescents will be unblinded. For practical and risk management reasons, *Field Supervisors* will be aware of which adolescents have been allocated to intervention and control groups, but they will not be involved in any outcome assessments. Similarly, the *Project Manager and Risk Management Team* will not be blinded for the purposes of the pilot trial, to prioritise participant safety.

Any breaches in blinding will be documented, and we will endeavour to change the fieldworker conducting subsequent assessments on a particular adolescent where unblinding has occurred.

4.7 Sample size determination

The sample size was calculated on the basis of one of our co-primary objectives which aims to provide preliminary evidence of signals of efficacy (direction and magnitude) of any effects of the intervention on depressive symptoms (measured by the PHQ-A) amongst adolescents in the intervention arm compared to the control group.

Statistical power was calculated to detect differences between two independent groups, in a two-sided test with an α of 0.05 and a power of $1–\beta=0.80$, for an effect size (Cohen's d) of 0.45. This effect size is based on findings from previous studies investigating digital psychological interventions, with effect size (Cohen's d) ranging from 0.24 to 0.57 (Arjadi et al., 2018; Fu et al., 2020; Lehtimaki et al., 2021). Given these findings, we chose a small-to-medium effect size of 0.45 to account for the limited number of studies conducted with our target population and in our study setting. We allowed for 25% attrition, based on a previous study with adolescents in the Agincourt setting (Pettifor et al., 2016). Given these assumptions, we aimed to recruit 200 participants at baseline. We calculated the required sample size using G*Power software, version 3.1.9.3 (Faul et al., 2007).

5. Data collection

5.1 Data collection schedule

There are 4 planned assessments for this pilot trial, including the baseline assessment and 3 follow-up assessments (at week 5, 11, and 24). More information can be found in Table 1.

5.2 Description of measures

Primary outcomes

(1) Feasibility and acceptability of the intervention. A mixed methods approach will be adopted to establish feasibility and acceptability.

- **Feasibility** will be assessed by collecting data on the following:

  (1) Recruitment (enrolment rate of eligible participants) and retention in trial at the end of the intervention period (11 weeks).

  (2) Feasibility of testing procedures and data collection methods, including assessment of completion rates.

  (3) Treatment adherence rates, where adherence is defined as having opened at least 4 out of 6 of the app modules and as having completed 3 out of 6 phone calls with the peer mentor (excluding the introductory call). We will complement the treatment adherence rates with engagement metrics collected via the app (number of times participants logged into the app, number of modules opened and completed, total time spent on the app, number of weekly activities set to do, and number of times the participant completed the weekly activities).

For peer review only - http://bmjopen.bmj.com/site/about/guidelines.xhtml

- **Acceptability** of the intervention and study procedures will be assessed via:

  (1) An acceptability questionnaire conducted at the end of intervention assessment (week 11) with all participants. The questionnaire consists of three measures: Acceptability of Intervention Measure (AIM), Intervention Appropriateness Measure (IAM), and Feasibility of Intervention Measure (FIM) (Weiner et al., 2017). Each measure consists of 4 items. The score for each measure ranges from 1 to 5 and is calculated by assigning scores to the response categories [1=completely disagree to 5=completely agree] and calculating an average score for each of the measures. No items need to be reverse coded. Higher scores indicate greater acceptability, appropriateness or feasibility. We will inquire about the acceptability of the app using these three measures (to both intervention and control group) and the acceptability of the peer mentor programme (to the intervention group only).

  (2) In-depth interviews with a subsample of participants. We estimate that interviews with 20 participants will provide a sufficient range of experiences and perspectives to reach data saturation. This subsample will contain participants from both the treatment and control arms (2:1 ratio) and will be stratified based on high versus low app engagement (i.e., for each participant in the control group, we will have 2 participants in the treatment group, one with low engagement and another with high engagement).

- ***Fidelity of delivery of the intervention*** will be assessed by collecting data on adherence and competence of Trained Peer Mentors.

  (1) Adherence of the peer mentors is defined as the number of sessions that meet at least 90% of criteria for adherence according to the training protocol. Independent raters will listen to a random sample (10%) of recordings of Peer Mentors' phone calls with participants and assess them against the training protocol.

  (2) Competence of the peer mentors will be expressed as a percentage based on their Competency Assessment Test. This test will include a written test and observation of skills through role-playing to assess Knowledge, Attitudes and Practices. Tests will be scored by the Trial Psychologist using a pre-determined scoring system.

(2) Signals of initial efficacy on depressive symptoms

This will be assessed using the PHQ-A score at end of intervention assessment (week 11). The PHQ-A is a widely used and well-established measure of adolescent depressive symptoms over the past two weeks (Kroenke et al., 2001). The questionnaire consists of 9 items. The PHQ-A total score ranges from 0 to 27 and is calculated by assigning scores to the response categories [0=not at all, 1=several days, 2=more than half the days, 3-nearly every day] and summing the score for each of the items. Higher scores indicate greater severity of depression: 0-4 No or Minimal depression, 5-9 Mild depression, 10-14 Moderate depression, 15-19 Moderately severe depression, 20-27 Severe depression. This measure has shown good psychometric properties with a sample of adolescents and young adults in South Africa and Kenya (Bhana et al., 2015; Cholera et al., 2014; Osborn et al., 2019). Participants will be asked to complete this scale at screening, mid-

For peer review only - http://bmjopen.bmj.com/site/about/guidelines.xhtml

intervention (week 5), end of intervention (week 11), and follow-up (week 24). Participants will also be asked to complete this scale as part of the symptom monitoring (week 2.5 and week 7.5).

Secondary outcomes

We aim to pilot locally adapted measures of the concepts below to examine their acceptability and variation and inform the development of a further larger trial.

Mental health outcomes

Generalised Anxiety Disorder (GAD-7). The GAD-7 is designed to assess the symptoms of generalised anxiety disorder over the previous 2 weeks (Spitzer et al., 2006). The questionnaire consists of 7 items. The GAD-7 total score ranges from 0 to 21 and is calculated by assigning scores to the response categories [0=not at all, 1=several days, 2=more than half the days, 3-nearly every day] and summing the score for each of the items. Higher scores represent increased anxiety: 0-5 mild; 6-10 moderate; 11-15 moderately severe anxiety; 15-21 severe anxiety. Studies from Sub-Saharan Africa examining the psychometric properties of the GAD-7 have demonstrated good internal consistency and performance characteristics with young adults in Zimbabwe and among Kenyan youth (Chibanda et al., 2016; Osborn et al., 2019). Participants will be asked to complete this scale at baseline (week 0), mid-intervention (week 5), end of intervention (week 11), and follow-up (week 24).

Conor-Davidson Resilience Scale (CD-RISC). The CD-RISC measures the ability to cope with traumatic stress (Connor and Davidson, 2003). The questionnaire consists of 10 items. The CD-RISC total score ranges from 0 to 40 and is calculated by assigning scores to the response categories [0=not true at all, 1=rarely true, 2=sometimes true, 3=often true, 4=true nearly all the time] and summing the score for each of the items. Higher scores represent higher resilience. CD-RISC-25 has been used in a sample of South African adolescents in public schools (Jørgensen and Seedat, 2008) and with homeless youth in Ghana (Asante and Meyer-Weitz, 2014). Participants will be asked to complete this scale at baseline (week 0) and end of intervention (week 11).

Brief Rumination Response Scale (RRS). The brief RRS measures brooding, defined as a passive comparison of one's current situation with some unachieved standard (Treynor et al., 2003). The questionnaire consists of 5 items. The brief RRS total score ranges from 0 to 15 and is calculated by assigning scores to the response categories [0=Almost never, 1=Sometimes, 2=Often, 3=Almost always] and summing the score for each of the items. Higher scores represent higher rumination/brooding. The full scale has been previously used among depressed Nigerian adolescents (Olaseni, 2018) and it is currently being used in a trial led by Prof. Stein with HIV-positive pregnant women in KwaZulu-Natal. Participants will be asked to complete this scale at baseline (week 0), mid intervention (week 5), end of intervention (week 11), and follow-up (week 24).

Warwick-Edinburgh Mental Wellbeing Scale (WEMWBS). The WEMWBS is a questionnaire to assess psychological functioning and emotional wellbeing (Tennant et al., 2007). The questionnaire consists of 14 items. The WEMWBS total score for the full-scale ranges from 14-70 and is calculated by assigning scores to the response categories [1=None of the time, 2=Rarely, 3=Some of the time, 4=Often, 5=Nearly every day] and summing the score for each of the items. The 7-item scale SWEMWBS, raw scores will be transformed as described in the authors'

For peer review only - http://bmjopen.bmj.com/site/about/guidelines.xhtml

conversion table. The scale has been used in South Africa, Tanzania, and Kenya and proved to be valid in a range of settings (e.g., public health, schools, clinical settings, etc.). The Cronbach's alpha for the WEMWBS in a sample of Kenyan adolescents was 0.70, indicating adequate reliability of this measure (Osborn et al., 2019). Participants will be asked to complete the full-scale (WEMWBS, 14-items) at baseline (week 0) and end of intervention (week 11), and the short form (SWEWMBS, 7-items) at mid-intervention (week 5) and follow-up (week 24).

Behavioural Activation for Depression Scale (BADS). The BADS measures when and how individuals become less avoidant and more activated over the course of treatment (Kanter et al., 2007). The questionnaire consists of 25 items. The BADS total score for the full-scale ranges from 0-150 and is calculated by assigning scores to the response categories [0=Very untrue, 1=Untrue, 2=Somewhat untrue, 3=Neutral, 4=Somewhat true, 5=True, 6=True] and summing the score for each of the items. Although most of the evidence comes from high income countries, several BA trials with adolescents have found an association between BADS and treatment effect (Chu et al., 2016; McCauley et al., 2016; Takagaki et al., 2016). Participants will be asked to complete the full-scale (25-items) at baseline (week 0) and end of intervention (week 11), the activation and social impairment subscales at mid-intervention (week 5), and the activation subscale at follow-up (week 24).

Cognition outcomes

Computerised cognitive tasks include computerised paradigms of affective control, social cognition, risk-taking and executive function. Participants will be asked to complete these tasks at baseline (week 0) and end of intervention (week 11).

Affective Set-Shifting: set-shifting was assessed with a modified version of the Wisconsin Card Sorting Task. In this version participants assign cards to one of four decks based on three sorting criteria: (i) the number of items on the card, (ii) the colour of the card, (iii) or the shape of the items (neutral version) or the emotional expression of the faces (affective version). Participants are not told the sorting rule but instead rely on the computerised feedback they receive to determine the correct rule. The sorting rules change suddenly, and participants need to adapt their sorting criterion (Schweizer et al., 2019).

Affective Backwards Digit Span: the backward digit span is an updating task in which participants are asked to recall digits that are serially presented, superimposed over neutral (e.g., scrambled face) or negative (e.g., sad face) images, in reverse order. The task assesses how many digits participants can remember in sequence and was adapted for computerised presentation (Jahanshahi et al., 2008).

Balloon Analogue Risk Task (BART): in this incentivised task, participants earn monetary rewards for inflating a balloon and choose when to stop and claim their reward. With each pump, the potential payoff increases, however if the balloon explodes no reward is given. This task assesses risk-seeking preferences under uncertainty (White et al., 2008).

Emotion recognition task: participants are shown images of faces showing different types of emotional expressions and are asked to classify the emotion that is expressed. They can choose

For peer review only - http://bmjopen.bmj.com/site/about/guidelines.xhtml

one out of four answer options: happy, sad, angry, neutral. Facial expressions are displayed at different intensities to vary task difficulty. Binary variable equal to 1 if facial expression was labelled correctly (Fuhrmann et al., 2016).

Matrix Reasoning Item Bank (MaRs-IB): participants complete a measure of abstract reasoning, where they must select one of several potential objects to complete the pattern of a matrix. The task requires participants to identify the missing element that completes a pattern and is frequently used to test fluid intelligence (Chierchia et al., 2019).

Risky behaviour and peer pressure outcomes

Participants will be asked to complete the risky behaviour outcomes at baseline (week 0) and end of intervention (week 11) using Audio Computer-Assisted Self-Interviewing (ACASI) software. We will measure 4 types of risky behaviours, and construct the following variables:

Risky sexual behaviour: we will create variables for the following measures: (1) *Coital debut before age of 15:* Binary variable equal to 1 if had sex before age 15; (2) *Pregnancy:* Binary variable equal to 1 if ever pregnant/gotten a woman pregnant; Discrete variable equal to the number of times pregnant/gotten a woman pregnant; Binary variable equal to 1 if unintended pregnancy; (3) *Unsafe sex:* Binary variable equal to 1 if unsafe sex; (4) *Multiple partners:* Binary variable equal to 1 if multiple partners in the past 12 months; (5) *Risky sex:* Binary variable equal to 1 if sex while high on alcohol/drugs; (6) *Transactional sex:* Binary variable equal to 1 if had sex in exchange for money/gifts

Substance use: the frequency of substance use will be assessed using the Alcohol, Smoking and Substance Involvement Screening Test for Youth (ASSIST-Y). The ASSIST-Y consists of six questions. For each substance listed, sum the scores for questions 2–6. More information about scoring and cut-offs can be found in (Humeniuk et al., 2016).

Delinquency: Binary variable equal to 1 if, in the past 30 days, the participant has hit, slapped, or physically hurt someone; or broken into a house, school, shop or other building without permission; or caused serious damage to property that did not belong to them, or stolen something.

Gambling: We will create (1) a binary variable equal to 1 if participant won/lost money from gambling or betting in the past 30 days; and (2) a continuous variable equal to the amount won/lost from gambling or betting

Peer pressure: We will create several categorical variables indicating how much pressure does the participant get from his/her friends to: (1) have sex; (2) smoke; (3) drink alcohol; (4) take drugs, where 0=no pressure, to 3=a lot of pressure.

Socioeconomic outcomes

We will measure economic preferences (time preference, risk preference, and loss aversion) using three incentivised tasks (Andersen et al., 2006; Kahneman and Tversky, 2018). In addition, we

For peer review only - http://bmjopen.bmj.com/site/about/guidelines.xhtml

will measure a range of socioeconomic outcomes, including measures of time use, human capital investment, and spending. Participants will be asked to complete these outcomes at baseline (week 0) and end of intervention (week 11). The description of these measures as well as details of this analysis will be documented in a separate analysis plan.

Covariates

Participants and participants' caregivers (if adolescent<18) were asked to complete a brief sociodemographic questionnaire about the household at baseline (week 0). We will also ask participants about trauma (also at baseline only). We will construct the following variables:

Assets. We will measure household assets using two measures, both of which will be assessed only at baseline. Both measures will be used as a control and for heterogeneity analysis: (1) The *Simple Poverty Scorecard Poverty-Assessment Tool for South Africa* (Schreiner, 2017). The questionnaire consists of 12 items. All points in the scorecard are non-negative integers, and total scores range from 0 (most likely below a poverty line) to 100 (least likely below a poverty line); and (2) *Large assets:* Participant's caregivers will be asked whether the household owns different items (e.g., house, motorcycle, jewellery, land, large or small livestock, poultry). We will sum the total number of items the household owns.

Food security. We assessed food insecurity using the six-item Short Form of the Household Food Security Scale (Economic Research Service USDA, 2012). Total score ranges from 0 (=high or marginal food security), to 6 (=very low food security).

Cost for children. Total spent on school-related expenses, clothes, and health care for all children in the household.

Transfers. Total household received in pensions, insurances, scholarships, government grants or lottery in the past 30 days.

Demographic measures: In addition to these household characteristics, we also collected the following demographic indicators: Age; Marital status; Country of origin; Total number of children born alive; Orphan status (2=double orphan, 1=single orphan, 0=not an orphan); Caregiver is not the parent; Living arrangements (binary variable equal to 1 if participant lives in the household without parents; and Education (Grade enrolled in; Highest education level; Missed school days: binary variable equal to 1 if participant missed more than one whole week of school at any one time during the past 12 months).

Trauma. Four brief questions about trauma were included as part of the survey. These questions were initially developed for use in a study of digitally delivered Cognitive Behavioural Therapy, supported by peer mentors, at the University of California Los Angeles (UCLA). The questions ask whether the participant has experienced physical, sexual, or emotional violence currently or in the past. We will construct a binary variable equal to 1 if the participant experienced any type of trauma.

For peer review only - http://bmjopen.bmj.com/site/about/guidelines.xhtml

6. General issues for statistical analysis

6.1 Post randomisation exclusions

The numbers (with percentages of the randomised population) of post-randomisation exclusions will be reported by trial arm and reasons summarised. The following participants will be excluded from the baseline table and the analysis of all outcomes:

- Adolescents who withdrew consent to use their data
- Adolescents for whom an entire record of fraudulent data was detected.

6.2 Population definitions

The analysis will be on the intention to treat (ITT) population; participants will be categorised in the arm they were randomised to, despite the allocation received, excluding the post-randomisation exclusions listed in section 6.1.

- ***Descriptive analysis population:*** Baseline demographic and clinical characteristics will be reported for all participants randomised minus post-randomisation exclusions (Table 2A).
- ***Comparative analysis population*:** Participants' outcomes will be reported for all participants randomised minus post-randomisation exclusions (Table 2B-2E).
- ***Safety population:*** Safety data will be reported for all participants randomised (Table 2F).

6.3 Missing data

Patterns in missing data will be explored for all analyses. Complete case analyses will be reported in all instances, but in the event of data which appear to be missing at random, we will also consider the use of imputation methods as appropriate. Multiple imputation by chained equations (MICE) is a multiple imputation method used to replace missing data values in a data set under certain assumptions about the data missingness. MICE operates under the assumption that given the variables used in the imputation procedure, the missing data are Missing At Random (MAR), which means that the probability that a value is missing depends only on observed values and not on unobserved values (Schafer and Graham, 2002).

7. Descriptive Analyses

7.1 Baseline characteristics of randomised groups

The following participant and household characteristics at trial entry will be described separately for the two randomised groups:

- *Individual characteristics:* age, sex, marital status, orphan status, number of children, highest educational level.
- *Household characteristics:* household asset index, large assets module, and food security
- *Outcomes at baseline:* mental health outcomes (PHQ-A score, GAD-7 score, CD-RISC score, RRS score, WEMWBS score, BADS score), cognition outcomes (all 5 tasks), risky behaviours and peer pressure, economic preferences, socioeconomic outcomes (employment, earnings, savings, consumption, aspirations).

For peer review only - http://bmjopen.bmj.com/site/about/guidelines.xhtml

Numbers (with percentages) for binary and categorical variables and means (and standard deviations), or medians (with lower and upper quartiles) for continuous variables will be presented.

7.2 Attrition

The number and percentage of losses to follow up among participants will be reported for the two trial arms, and the reasons will be recorded. All deaths of the participants will be reported separately.

We will compare the attrition rate by assigned treatment status to test whether the probability of attrition differs by treatment type. We will analyse:

$$Y_i = Treatment * \beta_1 + \boldsymbol{X_i \lambda} + \boldsymbol{\varepsilon_1}$$

'i' indexes individuals, Y_i is an indicator variable for whether the participant attrited from the round, $Treatment_I$ is an indicator variable for a person assigned to the treatment and X_i is a vector of prespecified covariates, including individual and household characteristics (age, sex, marital status, highest educational level, household asset index, and food security) and mental health outcomes at baseline. We will display this analysis for each round of data collection.

8. Comparative Analyses

Quantitative data will be analysed using Stata Version 17.0 and/or other appropriate statistical analysis packages under the direction of the trial statistician (StataCorp, 2021).

Participants will be analysed in the groups to which they are randomly assigned, regardless of deviation from the protocol or treatment received (ITT population) and the data analyst will be blind to arm allocation. We will use descriptives to explore patterns in the data followed by inferential statistics involving univariate and multivariable models. A two-tailed p-value <0.05 will be considered as statistically significant in the inferential analysis.

8.1 Primary analysis

(1) Feasibility and acceptability of the intervention. Our first co-primary objective (feasibility and acceptability) will be assessed using a mixed-methods approach. We will compute appropriate summary statistics (e.g., proportions, means, SDs) for each quantitative outcome and evaluate the feasibility of the trial based on the pre-defined progression criteria discussed earlier.

The number and percentage of participants screened, assessed for eligibility, randomised in the two arms, and retention rates (including completion rates) will be provided, as well as the number and reason for participants excluded from the study. This will be illustrated with a flowchart, summarising the flow of participants from screening and recruitment to the end of intervention assessment (11 week). See Figure 1.

Treatment adherence rates, acceptability, and fidelity of delivery of the intervention will be reported as shown in Table 2B-2D.

Qualitative interviews will be audio-recorded, transcribed verbatim and translated into English. Transcripts will be analysed using thematic analysis. We will follow Braun and Clarke's six phases of analysis (i.e. becoming familiar with the data, generating initial codes, searching for themes, reviewing themes, defining and naming themes, and producing the report) (Braun and Clarke,

For peer review only

2006). Coding will be done by two independent researchers. NVivo10, a computer program that aids in the sorting and management of qualitative data, will be used to facilitate the analysis.

(2) Initial efficacy on depressive symptoms

Our second co-primary objective (initial efficacy on depressive symptoms) will be based on the PHQ-A score (continuous) at 11 weeks. Outcomes will be compared between intervention and control groups using a multiple linear regression model and adjusting for covariates.
The primary inference will be estimated as per the following form:

$$\text{PHQscore}_i = \alpha + \beta \text{Treated}_i + \delta^T X_i + \varepsilon_i$$

where 'i' stands for individuals, PHQscore_i denotes the PHQ-A score at the end-of-intervention visit (week 11), Treated_i is an indicator variable equal to one for individuals assigned to receive the treatment and zero if the individual is assigned to the control group, and X_i is a vector of covariates that might affect depressive symptoms based on past literature and data collected from a previous study among adolescents in the study setting (Pettifor et al., 2016). These covariates will include: PHQ score measured at baseline, sex, age, and household asset index
We will also conduct the analysis only including PHQ-A score at baseline and with no further adjustements.

We will test the hypothesis that assignment to the treatment group has no effect on the outcome relative to the control group (β_1=0). We will use robust standard errors to allow for the presence of heteroskedasticity. Results from this analysis will be reported as per Table 2E.

As a secondary analysis, we will make use of the repeated measurements of the PHQ-A throughout the trial (up to 6 times per individual) to evaluate the treatment effects across time. We will rely on statistically advanced methods to compute this (mixed-effect model for repeated measures) and adjust for baseline scores. We will use generalized estimating equations (GEE) based approach which assumes nonvarying (or average) coefficient in the presence of clustering multivariable models with PHQ-A over time as the main outcome. Additional to these we will also run multilevel (hierarchical) models focus on estimating the aspects of the model that vary by group. We will explore various covariance structures forms to model the PHQs over time such as first order auto-regressive, structured and unstructured.

Further exploratory analysis might include choosing additional covariates as part of the primary inference by regressing outcome variables for part of the endline sample on various combinations of baseline covariates and selecting a combination of covariates that explains a large portion of the outcome variation. The choice of covariates is designed to improve the precision of treatment effect estimates by absorbing outcome variation. This exercise will be performed in the control group before endline data is available for the full sample.

For peer review only - http://bmjopen.bmj.com/site/about/guidelines.xhtml

Trial progression criteria

Progression criteria for a definitive RCT will be based on a traffic light system of green (continue to RCT), amber (make modifications in discussion with TSC; proceed with caution), and red (discussion with TSC to not proceed to the RCT) (Avery et al., 2017).

We will evaluate three criteria as follows:

**Criteria**	**Green**	**Amber**	**Red**
Enrolment (recruitment) rate of eligible participants	≥60%	<60%, ≥40%	<40%
Retention to trial at 11 weeks	≥90%	<90%, ≥50%	<50%
Share of participants that open at least 4 out of 6 of the app episodes	≥70%	<70%, ≥50%	<50%
Share of participants that have 3 out of 6 phone calls with the peer mentor	≥70%	<70%, ≥50%	<50%

8.2 Secondary analysis

As part of the secondary analyses, we will pilot locally adapted outcome measures of mental health, cognitive tasks, risky behaviours, socioeconomic measures, and collect descriptive data to inform the development of a further larger RCT.

For the cognition tasks, we will calculate overall performance and distributions, the correlation between tasks, and convergent and external validity of risk related-measures. For the mental health outcomes, we will perform standard psychometric tests to examine whether the instruments perform well in the study context and calculate bivariate correlations across variables of interest. Specifically, we will assess the measure's reliability (internal consistency and corrected item-total correlations), validity (construct validity, confirmatory factor analysis, and psychometric invariance), and acceptability (descriptive statistics, endorsement rates, missing data, and response-style bias).

We will also analyse treatment effects, but the analysis is exploratory, to inform future work, rather than confirmatory. We will focus on the direction and magnitude of effects. We will not correct p-values for multiple hypothesis testing as drawing conclusions about the statistical significance of effects is less of a focus of this analysis. We anticipate that some secondary analysis will be included in companion papers rather than the main paper.

8.3 Exploring possible mechanisms

Exploratory analyses will be conducted to identify mediators and moderators of the primary outcome at the end-of-intervention visit (week 11). Potential mediators will include executive function and social cognition, as well as resilience, rumination, and functioning. We will decompose the total treatment effect on the primary outcome into (i) an indirect effect through one or more mediators, and (ii) the direct effect of the intervention not captured by these mediator(s) following Acharya et al. (2016). Moderators will include age, education, household asset index, suicide risk, and severity of depressive symptoms at screening.

For peer review only - http://bmjopen.bmj.com/site/about/guidelines.xhtml

For peer review only

8.4 Sensitivity analysis

A multiple imputation analysis will be performed for the primary outcome if attrition exceeds 5%. The multiple imputation model will include baseline characteristics and outcome measures collected prior to the missing assessment, which are associated with missing status (Enders and London, 2010; Xu et al., 2020).

We might also consider running the primary analysis using the per-protocol population, which consists of the ITT population but excludes any participants defined as having a major protocol deviation (see section 10.1).

9. Safety data analysis

Serious adverse events (SAEs) and whether they were 'related' to research procedures or 'unexpected' will be listed by trial arm. As the trial intervention involves increased assessments and contact, the number of safety events detected and reported is likely to be higher in the intervention arm.

10. Protocol non-compliances

A protocol non-compliance is defined as a failure to adhere to the protocol such as the wrong intervention being administered, incorrect data being collected and documented, errors in applying inclusion/exclusion criteria or missed follow-up visits due to error. The overall number of participants experiencing a protocol non-compliance and the total number of non-compliances will be reported. Treatment group will be cross tabulated with type of major and minor deviation.

10.1 Major

A major protocol non-compliance is any failure to comply with the final study protocol as approved by ethics committees, resulting from error, fraud or misconduct and results in the exclusion of a patient from the study. Fraudulent data will be defined as a major protocol non-compliance in this pilot trial.

10.2 Minor

The following will be defined as minor protocol non-compliances:

- *Participants randomised in error.* These include adolescents: (1) Whose informed consent is not fully documented; (2) Aged below 15 or above 19 years; (3) Whose PHQ-A score <5 points at screening; (4) Whose PHQ-A score >19 at screening; (5) With severe psychiatric illness, or a life-threatening or other serious physical illness; (6) With suicidal ideation/ thoughts with specific plans and means identified; (7) Who are planning to move away from the study area in the next 12 weeks; (8) Who do not speak and read sufficiently in Xitsonga
- *Participants who do not receive the allocated intervention*. These include adolescents: (1) In the intervention arm who did not receive the intervention as planned in the protocol; (2) In the control group who received some or all the intervention.
- *The assessor becomes unblinded prior to or during an assessment.*

For peer review only - http://bmjopen.bmj.com/site/about/guidelines.xhtml

11. References

Acharya, A., Blackwell, M., Sen, M., 2016. Explaining Causal Findings Without Bias: Detecting and Assessing Direct Effects. Am. Polit. Sci. Rev. 110, 512–529. https://doi.org/10.1017/S0003055416000216

Andersen, S., Harrison, G.W., Lau, M.I., Rutström, E.E., 2006. Elicitation using multiple price list formats. Exp. Econ. 9, 383–405. https://doi.org/10.1007/s10683-006-7055-6

Arjadi, R., Nauta, M.H., Scholte, W.F., Hollon, S.D., Chowdhary, N., Suryani, A.O., Uiterwaal, C.S.P.M., Bockting, C.L.H., 2018. Internet-based behavioural activation with lay counsellor support versus online minimal psychoeducation without support for treatment of depression: a randomised controlled trial in Indonesia. The Lancet Psychiatry 5, 707–716. https://doi.org/10.1016/S2215-0366(18)30223-2

Asante, K.O., Meyer-Weitz, A., 2014. Measuring resilience among homeless youth: psychometric assessment of the Connor-Davidson Resilience Scale in Ghana. J. Psychol. Africa 24, 321–326. https://doi.org/10.1080/14330237.2014.980620

Avery, K.N.L., Williamson, P.R., Gamble, C., Francischetto, E.O.C., Metcalfe, C., Davidson, P., Williams, H., Blazeby, J.M., Blencowe, N., Bugge, C., Campbell, M., Collinson, M., Cooper, C., Darbyshire, J., Dimairo, M., Doré, C., Eldridge, S., Farrin, A., Foster, N., Gilbody, S., Goodacre, S., Hampson, L., Kolias, A.G., Lamb, S., Lane, A., Maguire, L., Norrie, J., Pickering, R., Shorter, G., Treweek, S., 2017. Informing efficient randomised controlled trials: exploration of challenges in developing progression criteria for internal pilot studies. BMJ Open 7, e013537. https://doi.org/10.1136/BMJOPEN-2016-013537

Bhana, A., Rathod, S.D., Selohilwe, O., Kathree, T., Petersen, I., 2015. The validity of the Patient Health Questionnaire for screening depression in chronic care patients in primary health care in South Africa. BMC Psychiatry 15, 118. https://doi.org/10.1186/s12888-015-0503-0

Braun, V., Clarke, V., 2006. Using thematic analysis in psychology. Qual. Res. Psychol. 3, 77–101. https://doi.org/10.1191/1478088706qp063oa

Chan, A.W., Tetzlaff, J.M., Altman, D.G., Laupacis, A., Gøtzsche, P.C., Krleža-Jerić, K., Hróbjartsson, A., Mann, H., Dickersin, K., Berlin, J.A., Doré, C.J., Parulekar, W.R., Summerskill, W.S.M., Groves, T., Schulz, K.F., Sox, H.C., Rockhold, F.W., Rennie, D., Moher, D., 2013. SPIRIT 2013 statement: Defining standard protocol items for clinical trials. Ann. Intern. Med. 158, 200–207. https://doi.org/10.7326/0003-4819-158-3-201302050-00583

Chibanda, D., Verhey, R., Gibson, L.J., Munetsi, E., Machando, D., Rusakaniko, S., Munjoma, R., Araya, R., Weiss, H.A., Abas, M., 2016. Validation of screening tools for depression and anxiety disorders in a primary care population with high HIV prevalence in Zimbabwe. J. Affect. Disord. 198, 50–55. https://doi.org/10.1016/j.jad.2016.03.006

Chierchia, G., Fuhrmann, D., Knoll, L.J., Pi-Sunyer, B.P., Sakhardande, A.L., Blakemore, S.-J., 2019. The matrix reasoning item bank (MaRs-IB): novel, open-access abstract reasoning items for adolescents and adults. R. Soc. Open Sci. 6, 190232. https://doi.org/10.1098/rsos.190232

Cholera, R., Gaynes, B.N., Pence, B.W., Bassett, J., Qangule, N., Macphail, C., Bernhardt, S., Pettifor, A., Miller, W.C., 2014. Validity of the patient health questionnaire-9 to screen for depression in a high-HIV burden primary healthcare clinic in Johannesburg, South Africa. J. Affect. Disord. 167, 160–166. https://doi.org/10.1016/j.jad.2014.06.003

For peer review only - http://bmjopen.bmj.com/site/about/guidelines.xhtml

Chu, B.C., Crocco, S.T., Esseling, P., Areizaga, M.J., Lindner, A.M., Skriner, L.C., 2016. Transdiagnostic group behavioral activation and exposure therapy for youth anxiety and depression: Initial randomized controlled trial. Behav. Res. Ther. 76, 65–75. https://doi.org/10.1016/j.brat.2015.11.005

Economic Research Service USDA, 2012. Food Security Survey Module: Six-Item Short Form.

Enders, C.K., London, N.Y., 2010. Applied missing data analysis.

Faul, F., Erdfelder, E., Lang, A.G., Buchner, A., 2007. G*Power 3: A flexible statistical power analysis program for the social, behavioral, and biomedical sciences. Behav. Res. Methods 2007 392 39, 175–191. https://doi.org/10.3758/BF03193146

Fu, Z., Burger, H., Arjadi, R., Bockting, C.L.H., 2020. Effectiveness of digital psychological interventions for mental health problems in low-income and middle-income countries: a systematic review and meta-analysis. The Lancet Psychiatry 7, 851–864. https://doi.org/10.1016/S2215-0366(20)30256-X

Fuhrmann, D., Knoll, L.J., Sakhardande, A.L., Speekenbrink, M., Kadosh, K.C., Blakemore, S.-J., 2016. Perception and recognition of faces in adolescence. Sci. Rep. 6, 33497. https://doi.org/10.1038/srep33497

Goin, D.E., Pearson, R.M., Craske, M.G., Stein, A., Pettifor, A., Lippman, S.A., Kahn, K., Neilands, T.B., Hamilton, E.L., Selin, A., MacPhail, C., Wagner, R.G., Gomez-Olive, F.X., Twine, R., Hughes, J.P., Agyei, Y., Laeyendecker, O., Tollman, S., Ahern, J., 2019. Depression and incident HIV in adolescent girls and young women in HPTN 068: Targets for prevention and mediating factors. Am. J. Epidemiol. https://doi.org/10.1093/aje/kwz238

Humeniuk, R., Holmwood, C., Beshara, M., Kambala, A., 2016. ASSIST-Y V1.0: First-Stage Development of the WHO Alcohol, Smoking and Substance Involvement Screening Test (ASSIST) and Linked Brief Intervention for Young People. J. Child Adolesc. Subst. Abuse 25, 384–390. https://doi.org/10.1080/1067828X.2015.1049395

Jacobson, N.S., Martell, C.R., Dimidjian, S., 2006. Behavioral Activation Treatment for Depression: Returning to Contextual Roots. Clin. Psychol. Sci. Pract. 8, 255–270. https://doi.org/10.1093/clipsy.8.3.255

Jahanshahi, M., Saleem, T., Ho, A.K., Fuller, R., Dirnberger, G., 2008. A preliminary investigation of the running digit span as a test of working memory. Behav. Neurol. 20, 17–25. https://doi.org/10.3233/BEN-2008-0212

Jørgensen, I.E., Seedat, S., 2008. Factor structure of the Connor-Davidson Resilience Scale in South African adolescents. Int. J. Adolesc. Med. Health 20, 23–32. https://doi.org/10.1515/IJAMH.2008.20.1.23

Kahn, K., Collinson, M.A., Gómez-Olivé, F.X., Mokoena, O., Twine, R., Mee, P., Afolabi, S.A., Clark, B.D., Kabudula, C.W., Khosa, A., Khoza, S., Shabangu, M.G., Silaule, B., Tibane, J.B., Wagner, R.G., Garenne, M.L., Clark, S.J., Tollman, S.M., 2012. Profile: Agincourt health and socio-demographic surveillance system. Int. J. Epidemiol. 41, 988–1001. https://doi.org/10.1093/ije/dys115

Kahneman, D., Tversky, A., 2018. Prospect theory: An analysis of decision under risk, in: Experiments in Environmental Economics. Taylor and Francis Inc., pp. 143–172. https://doi.org/10.2307/1914185

Kessler, R.C., Berglund, P., Demler, O., Jin, R., Merikangas, K.R., Walters, E.E., 2005. Lifetime prevalence and age-of-onset distributions of DSM-IV disorders in the national comorbidity survey replication. Arch. Gen. Psychiatry. https://doi.org/10.1001/archpsyc.62.6.593

Lehtimaki, S., Martic, J., Wahl, B., Foster, K.T., Schwalbe, N., 2021. Evidence on Digital

For peer review only - http://bmjopen.bmj.com/site/about/guidelines.xhtml

Mental Health Interventions for Adolescents and Young People: Systematic Overview. JMIR Ment Heal. 8, e25847. https://doi.org/10.2196/25847
McCauley, E., Gudmundsen, G., Schloredt, K., Martell, C., Rhew, I., Hubley, S., Dimidjian, S., 2016. The Adolescent Behavioral Activation Program: Adapting Behavioral Activation as a Treatment for Depression in Adolescence. J. Clin. Child Adolesc. Psychol. 45, 291–304. https://doi.org/10.1080/15374416.2014.979933
Olaseni, J.T., 2018. Rumination and Academic Hardiness as Predicators of Suicidal Ideation among Nigerian Adolescents. Abnorm. Behav. Psychol. 04. https://doi.org/10.4172/2472-0496.1000133
Osborn, T.L., Venturo-Conerly, K.E., Wasil, A.R., Schleider, J.L., Weisz, J.R., 2019. Depression and Anxiety Symptoms, Social Support, and Demographic Factors Among Kenyan High School Students. J. Child Fam. Stud. 1–12. https://doi.org/10.1007/s10826-019-01646-8
Pettifor, A., MacPhail, C., Selin, A., Gómez-Olivé, F.X., Rosenberg, M., Wagner, R.G., Mabuza, W., Hughes, J.P., Suchindran, C., Piwowar-Manning, E., Wang, J., Twine, R., Daniel, T., Andrew, P., Laeyendecker, O., Agyei, Y., Tollman, S., Kahn, K., The HPTN 068 protocol team, 2016. HPTN 068: A Randomized Control Trial of a Conditional Cash Transfer to Reduce HIV Infection in Young Women in South Africa—Study Design and Baseline Results. AIDS Behav. 20, 1863–1882. https://doi.org/10.1007/S10461-015-1270-0/FIGURES/3
Schafer, J.L., Graham, J.W., 2002. Missing data: Our view of the state of the art. Psychol. Methods 7, 147–177. https://doi.org/10.1037/1082-989X.7.2.147
Schreiner, M., 2017. Simple Poverty Scorecard Poverty-Assessment Tool South Africa.
Schweizer, S., Parker, J., Leung, J.T., Griffin, C., Blakemore, S.-J., 2019. Age-related differences in affective control and its association with mental health difficulties. Dev. Psychopathol. 1–13. https://doi.org/10.1017/S0954579419000099
StataCorp, 2021. Stata Statistical Software: Release 17.
Takagaki, K., Okamoto, Yasumasa, Jinnin, R., Mori, A., Nishiyama, Y., Yamamura, T., Yokoyama, S., Shiota, S., Okamoto, Yuri, Miyake, Y., Ogata, A., Kunisato, Y., Shimoda, H., Kawakami, N., Furukawa, T.A., Yamawaki, S., 2016. Behavioral activation for late adolescents with subthreshold depression: a randomized controlled trial. Eur. Child Adolesc. Psychiatry 25, 1171–1182. https://doi.org/10.1007/s00787-016-0842-5
Thapar, A., Collishaw, S., Pine, D.S., Thapar, A.K., 2012. Depression in adolescence. Lancet. https://doi.org/10.1016/S0140-6736(11)60871-4
Weiner, B.J., Lewis, C.C., Stanick, C., Powell, B.J., Dorsey, C.N., Clary, A.S., Boynton, M.H., Halko, H., 2017. Psychometric assessment of three newly developed implementation outcome measures. Implement. Sci. 12, 1–12. https://doi.org/10.1186/S13012-017-0635-3/TABLES/3
White, T.L., Lejuez, C.W., de Wit, H., 2008. Test-Retest Characteristics of the Balloon Analogue Risk Task (BART). Exp. Clin. Psychopharmacol. 16, 565–570. https://doi.org/10.1037/a0014083
Xu, X., Xia, L., Zhang, Q., Wu, S., Wu, M., Liu, H., 2020. The ability of different imputation methods for missing values in mental measurement questionnaires. BMC Med. Res. Methodol. 20, 1–9. https://doi.org/10.1186/S12874-020-00932-0/TABLES/3

For peer review only - http://bmjopen.bmj.com/site/about/guidelines.xhtml

12. Table and Figures

Table 1. DoBAt's time schedule of enrolment, interventions, assessments, and visits for participants

	STUDY PERIOD							
Assessment	Enrolment	Allocation	Post-allocation					Close-out
**TIME POINT (in weeks)**	$-t_1$	0	1	2.5	5	7.5	11	24 WKS
**ENROLMENT**								
Eligibility screen (school survey)	X							
Informed consent	X							
Allocation		X						
Phone delivery		X						
**INTERVENTION**								
Intervention arm: Kuamsha app & Peer Mentor calls								
Control arm: Kuchunguza app								
**ASSESSMENTS**								
School survey: PHQ-A, sociodemographic questions & eligibility checklist	X							
Baseline assessment: demographic questionnaire, GAD-7, CD-RISC, RRS, WEMWBS, BADS, trauma, five social-affective and cognition tasks, risky behaviours, and socioeconomic outcomes	X							
Symptom monitoring: PHQ-A				X		X		
Mid-intervention assessment: PHQ-A, GAD-7, RRS, WEMWBS short form, BADS (activation & social impairment subscales)					X			
Post-intervention assessment: same as baseline + PHQA+ acceptability questionnaire & IDIs with subsample							X	
Follow-up assessment: PHQ-A, GAD-7, RRS, BADS activation subscale, WEMWBS short form								X

Table 2. Dummy tables

TABLE 2A. Baseline comparability of randomised groups		
	**Treatment group** (N=XX)	**Control group** (N=XX)
**Individual characteristics**<br>• Age<br>• Sex<br>• Marital status<br>• Orphan status<br>• Number of children<br>• Highest educational level<br>• Employment status		
**Household characteristics**<br>• Household asset index<br>• Food security<br>• Maternal/paternal education level		
**Outcomes at baseline**<br>• PHQ-A score<br>• GAD-7 score<br>• CD-RISC score<br>• RRS score<br>• WEMWBS score<br>• BADS score<br>• Affective Set-Shifting		

review only

For peer review only - http://bmjopen.bmj.com/site/about/guidelines.xhtml

• Affective Backwards Digit Span<br>• Balloon Analogue Risk Task (BART)<br>• Emotion recognition task<br>• Matrix Reasoning Item Bank (MaRs-IB)<br>• Risky behaviours<br>• Peer pressure<br>• Economic preferences<br>• Employment<br>• Earnings<br>• Savings<br>• Consumption<br>• Aspirations		

Comparative analysis:

TABLE 2B. FEASIBILITY	
	**Treatment group** ***(Kuamsha app)*** (N=XX)
**Number of participants with data**	
**Recruitment and retention:**<br>• Enrolment rate of eligible participants<br>• Retention at the end of the intervention (11 weeks)	
**Treatment adherence rate:**<br>• N (%) opened ≥4 modules/episodes in the app<br>• N (%) had ≥3 phone calls with the peer mentors (excluding introductory call)	
**Kuamsha app engagement metrics**<br>• Number of logins<br>• Number of episodes opened<br>• Number of episodes completed<br>• Total time spent on app<br>• Number of set up weekly activities<br>• Number of completed weekly activities	
	**Control group** ***(Kuchunguza app)*** (N=XX)
**Number of participants with data**	
**Kuchunguza app engagement metrics**<br>• Number of videos opened<br>• Number of videos completed<br>• Total time spent on app	

TABLE 2C. ACCEPTABILITY		
	**Treatment group** (N=XX)	**Control group** (N=XX)

For peer review only - http://bmjopen.bmj.com/site/about/guidelines.xhtml

Acceptability of using the app<br>• AIM score<br>• IAM score<br>• FIM score	Median {IQR} [Range]	Median {IQR} [Range]
Acceptability of the peer mentor programme (only treatment group)<br>• AIM score<br>• IAM score<br>• FIM score	Median {IQR} [Range]	N. A

TABLE 2D. FIDELITY OF DELIVERY OF THE INTERVENTION	
	**Treatment group** (N of peer mentors=XX)
**Adherence and competence of peer mentors:**<br>• N (%) sessions that meet ≥90% criteria for adherence<br>• Competency Assessment score	

TABLE 2E. INITIAL EFFICACY			
	**Treatment group** (N=XX)	**Control group** (N=XX)	**Mean difference (95% CI)**
Primary inference:<br>PHQ-A score at end of intervention (11 weeks)<br>• Mean (SD)<br>• Unknown			

Safety population:

TABLE 2F. ADVERSE EVENTS						
**AE number**	**Treatment allocation**	**Description**	**Severity**	**Related**	**Action taken**	**Outcome**

For peer review only

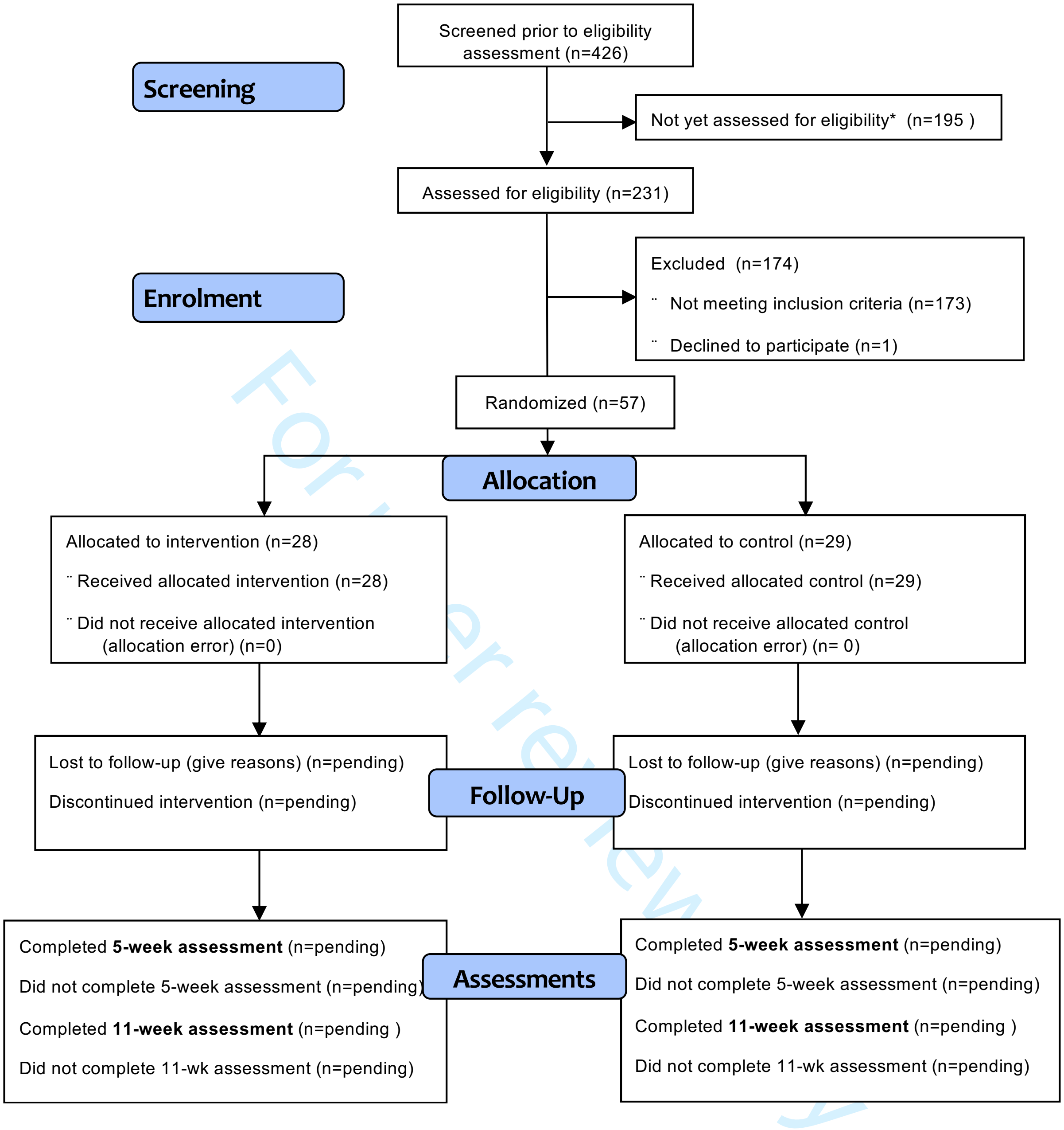

Figure 1. CONSORT flow diagram for DoBAt study

13. Approval

For peer review only - http://bmjopen.bmj.com/site/about/guidelines.xhtml

Lead Medical Statistician	Name: Professor Eustasius Musenge	
	Signature	Date: 03/11/22
Chief Co-Investigators	Name: Professor Kathleen Kahn	
	Signature	Date: 03/11/22
	Name: Professor Alan Stein	
	Signature	Date: 03/11/22
Chair of DSMB	Name: Professor Bonga Chiliza	
	Signature	Date 04/11/2022
Chair of Trial Steering Committee	Name: Professor Roz Shafran	
	Signature	Date 04.11.22

For peer review only - http://bmjopen.bmj.com/site/about/guidelines.xhtml

SPIRIT
STANDARD PROTOCOL ITEMS: RECOMMENDATIONS FOR INTERVENTIONAL TRIALS

SPIRIT 2013 Checklist: Recommended items to address in a clinical trial protocol and related documents*

Section/item	Item No	Description	Page
**Administrative information**			
Title	1	Descriptive title identifying the study design, population, interventions, and, if applicable, trial acronym	Page 1
Trial registration	2a	Trial identifier and registry name. If not yet registered, name of intended registry	Page 2
	2b	All items from the World Health Organization Trial Registration Data Set	Sup. Material (Table 5)
Protocol version	3	Date and version identifier	Sup. Material (Table 5)
Funding	4	Sources and types of financial, material, and other support	Page 14
Roles and responsibilities	5a	Names, affiliations, and roles of protocol contributors	Pages 1 & 14
	5b	Name and contact information for the trial sponsor	Sup. Material (Table 5)
	5c	Role of study sponsor and funders, if any, in study design; collection, management, analysis, and interpretation of data; writing of the report; and the decision to submit the report for publication, including whether they will have ultimate authority over any of these activities	Page 14
	5d	Composition, roles, and responsibilities of the coordinating centre, steering committee, endpoint adjudication committee, data management team, and other individuals or groups overseeing the trial, if applicable (see Item 21a for data monitoring committee)	Sup. Material (Table 1)

For peer review only

Introduction			
Background and rationale	6a	Description of research question and justification for undertaking the trial, including summary of relevant studies (published and unpublished) examining benefits and harms for each intervention	Pages 3-4
	6b	Explanation for choice of comparators	Page 6
Objectives	7	Specific objectives or hypotheses	Page 4
Trial design	8	Description of trial design including type of trial (eg, parallel group, crossover, factorial, single group), allocation ratio, and framework (eg, superiority, equivalence, noninferiority, exploratory)	Page 5
**Methods: Participants, interventions, and outcomes**			
Study setting	9	Description of study settings (eg, community clinic, academic hospital) and list of countries where data will be collected. Reference to where list of study sites can be obtained	Page 5
Eligibility criteria	10	Inclusion and exclusion criteria for participants. If applicable, eligibility criteria for study centres and individuals who will perform the interventions (eg, surgeons, psychotherapists)	Pages 5-6
Interventions	11a	Interventions for each group with sufficient detail to allow replication, including how and when they will be administered	Pages 6-7 & Sup. Material (Tables 2-4)
	11b	Criteria for discontinuing or modifying allocated interventions for a given trial participant (eg, drug dose change in response to harms, participant request, or improving/worsening disease)	n/a
	11c	Strategies to improve adherence to intervention protocols, and any procedures for monitoring adherence (eg, drug tablet return, laboratory tests)	Pages 6-8
	11d	Relevant concomitant care and interventions that are permitted or prohibited during the trial	Page 6

For peer review only - http://bmjopen.bmj.com/site/about/guidelines.xhtml

Outcomes	12	Primary, secondary, and other outcomes, including the specific measurement variable (eg, systolic blood pressure), analysis metric (eg, change from baseline, final value, time to event), method of aggregation (eg, median, proportion), and time point for each outcome. Explanation of the clinical relevance of chosen efficacy and harm outcomes is strongly recommended	Pages 7-9
Participant timeline	13	Time schedule of enrolment, interventions (including any run-ins and washouts), assessments, and visits for participants. A schematic diagram is highly recommended (see Figure)	Page 10 (Table 2, Figure 1)
Sample size	14	Estimated number of participants needed to achieve study objectives and how it was determined, including clinical and statistical assumptions supporting any sample size calculations	Page 12
Recruitment	15	Strategies for achieving adequate participant enrolment to reach target sample size	Page 10
**Methods: Assignment of interventions (for controlled trials)**			
Allocation:			
Sequence generation	16a	Method of generating the allocation sequence (eg, computer-generated random numbers), and list of any factors for stratification. To reduce predictability of a random sequence, details of any planned restriction (eg, blocking) should be provided in a separate document that is unavailable to those who enrol participants or assign interventions	Page 11
Allocation concealment mechanism	16b	Mechanism of implementing the allocation sequence (eg, central telephone; sequentially numbered, opaque, sealed envelopes), describing any steps to conceal the sequence until interventions are assigned	Page 11
Implementation	16c	Who will generate the allocation sequence, who will enrol participants, and who will assign participants to interventions	Page 11
Blinding (masking)	17a	Who will be blinded after assignment to interventions (eg, trial participants, care providers, outcome assessors, data analysts), and how	Page 11
	17b	If blinded, circumstances under which unblinding is permissible, and procedure for revealing a participant's allocated intervention during the trial	Page 11
**Methods: Data collection, management, and analysis**			

Data collection methods	18a	Plans for assessment and collection of outcome, baseline, and other trial data, including any related processes to promote data quality (eg, duplicate measurements, training of assessors) and a description of study instruments (eg, questionnaires, laboratory tests) along with their reliability and validity, if known. Reference to where data collection forms can be found, if not in the protocol	Pages 7-11
	18b	Plans to promote participant retention and complete follow-up, including list of any outcome data to be collected for participants who discontinue or deviate from intervention protocols	Sup. Material (SAP)
Data management	19	Plans for data entry, coding, security, and storage, including any related processes to promote data quality (eg, double data entry; range checks for data values). Reference to where details of data management procedures can be found, if not in the protocol	Sup. Material (SAP)
Statistical methods	20a	Statistical methods for analysing primary and secondary outcomes. Reference to where other details of the statistical analysis plan can be found, if not in the protocol	Pages 11-12 & Sup. Material (SAP)
	20b	Methods for any additional analyses (eg, subgroup and adjusted analyses)	Pages 11-12 & Sup. Material (SAP)
	20c	Definition of analysis population relating to protocol non-adherence (eg, as randomised analysis), and any statistical methods to handle missing data (eg, multiple imputation)	Pages 11-12 & Sup. Material (SAP)
**Methods: Monitoring**			
Data monitoring	21a	Composition of data monitoring committee (DMC); summary of its role and reporting structure; statement of whether it is independent from the sponsor and competing interests; and reference to where further details about its charter can be found, if not in the protocol. Alternatively, an explanation of why a DMC is not needed	Sup. Material (Table 1)
	21b	Description of any interim analyses and stopping guidelines, including who will have access to these interim results and make the final decision to terminate the trial	Sup. Material (SAP)

For peer review only - http://bmjopen.bmj.com/site/about/guidelines.xhtml

Harms	22	Plans for collecting, assessing, reporting, and managing solicited and spontaneously reported adverse events and other unintended effects of trial interventions or trial conduct	Page 13
Auditing	23	Frequency and procedures for auditing trial conduct, if any, and whether the process will be independent from investigators and the sponsor	N/A
**Ethics and dissemination**			
Research ethics approval	24	Plans for seeking research ethics committee/institutional review board (REC/IRB) approval	Page 13
Protocol amendments	25	Plans for communicating important protocol modifications (eg, changes to eligibility criteria, outcomes, analyses) to relevant parties (eg, investigators, REC/IRBs, trial participants, trial registries, journals, regulators)	Sup. Material (Table 5)
Consent or assent	26a	Who will obtain informed consent or assent from potential trial participants or authorised surrogates, and how (see Item 32)	Page 13
	26b	Additional consent provisions for collection and use of participant data and biological specimens in ancillary studies, if applicable	N/A
Confidentiality	27	How personal information about potential and enrolled participants will be collected, shared, and maintained in order to protect confidentiality before, during, and after the trial	Page 13
Declaration of interests	28	Financial and other competing interests for principal investigators for the overall trial and each study site	Page 14
Access to data	29	Statement of who will have access to the final trial dataset, and disclosure of contractual agreements that limit such access for investigators	Sup. Material (SAP)
Ancillary and post-trial care	30	Provisions, if any, for ancillary and post-trial care, and for compensation to those who suffer harm from trial participation	N/A
Dissemination policy	31a	Plans for investigators and sponsor to communicate trial results to participants, healthcare professionals, the public, and other relevant groups (eg, via publication, reporting in results databases, or other data sharing arrangements), including any publication restrictions	Page 14
	31b	Authorship eligibility guidelines and any intended use of professional writers	Page 14

For peer review only - http://bmjopen.bmj.com/site/about/guidelines.xhtml

	31c	Plans, if any, for granting public access to the full protocol, participant-level dataset, and statistical code	Sup. Material (Table 5)
Appendices			
Informed consent materials	32	Model consent form and other related documentation given to participants and authorised surrogates	Sup. Material
Biological specimens	33	Plans for collection, laboratory evaluation, and storage of biological specimens for genetic or molecular analysis in the current trial and for future use in ancillary studies, if applicable	N/A

*It is strongly recommended that this checklist be read in conjunction with the SPIRIT 2013 Explanation & Elaboration for important clarification on the items. Amendments to the protocol should be tracked and dated. The SPIRIT checklist is copyrighted by the SPIRIT Group under the Creative Commons "Attribution-NonCommercial-NoDerivs 3.0 Unported" license.

For peer review only
